# Proteome‐scale mapping of binding sites in the unstructured regions of the human proteome

**DOI:** 10.15252/msb.202110584

**Published:** 2022-01-19

**Authors:** Caroline Benz, Muhammad Ali, Izabella Krystkowiak, Leandro Simonetti, Ahmed Sayadi, Filip Mihalic, Johanna Kliche, Eva Andersson, Per Jemth, Norman E Davey, Ylva Ivarsson

**Affiliations:** ^1^ Department of Chemistry ‐ BMC Uppsala University Uppsala Sweden; ^2^ Division of Cancer Biology The Institute of Cancer Research London UK; ^3^ Department of Medical Biochemistry and Microbiology Uppsala University Uppsala Sweden

**Keywords:** intrinsically disordered regions, peptides, phage display, protein–protein interactions, short linear motifs, Methods & Resources, Proteomics

## Abstract

Specific protein–protein interactions are central to all processes that underlie cell physiology. Numerous studies have together identified hundreds of thousands of human protein–protein interactions. However, many interactions remain to be discovered, and low affinity, conditional, and cell type‐specific interactions are likely to be disproportionately underrepresented. Here, we describe an optimized proteomic peptide‐phage display library that tiles all disordered regions of the human proteome and allows the screening of ~ 1,000,000 overlapping peptides in a single binding assay. We define guidelines for processing, filtering, and ranking the results and provide PepTools, a toolkit to annotate the identified hits. We uncovered >2,000 interaction pairs for 35 known short linear motif (SLiM)‐binding domains and confirmed the quality of the produced data by complementary biophysical or cell‐based assays. Finally, we show how the amino acid resolution‐binding site information can be used to pinpoint functionally important disease mutations and phosphorylation events in intrinsically disordered regions of the proteome. The optimized human disorderome library paired with PepTools represents a powerful pipeline for unbiased proteome‐wide discovery of SLiM‐based interactions.

## Graphical abstract

An optimized phage peptidome that tiles the disordered regions of the human proteome is presented, allowing the field of motif‐based interactions to transition into high‐throughput. Guidelines and tools for data analysis are provided.

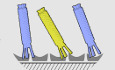

## Introduction

System‐wide insights into protein–protein interactions (PPIs) are crucial for a comprehensive description of cellular function and organization, and a molecular understanding of genotype‐to‐phenotype relationships. Impressive advances are being made toward illuminating the human interactome. For example, Luck *et al* (2020) recently provided the human reference interactome (HuRI), a map of about 53,000 human PPIs generated by all‐by‐all yeast‐two‐hybrid (Y2H) screening. Moreover, Huttlin *et al* (2021) released BioPlex 3.0, a dataset generated through affinity‐purification coupled to mass spectrometry (AP‐MS) that contains close to 120,000 interactions. However, a hidden interactome of low affinity, transient, and conditional interactions remains undiscovered. A significant portion of these unknown interactions are likely mediated by short linear motifs (SLiMs) found in the intrinsically disordered regions (IDRs) of the human proteome (Tompa *et al*, 2014). Given that IDRs are predicted to constitute up to 40% of the residues in higher eukaryotic proteomes (Pancsa & Tompa, 2012; Xue *et al*, 2012), the consensus is that tens of thousands of human motif‐based interactions remain undiscovered.

Here, we focus on proteome‐wide screening of SLiM‐based interactions involving a folded domain in one protein and a short peptide present in an IDR in another protein. On average, a SLiM interface buries only 3–4 residues in the binding pocket of the folded binding partner and the interactions are often of low‐to‐mid micromolar affinities (Van Roey *et al*, 2014; Ivarsson & Jemth, 2019). SLiM‐based interactions are prevalent and crucial for dynamic processes such as cell signaling and regulation. They commonly direct the transient complex association, scaffolding, modification state, half‐life, and localization of a protein. The Eukaryotic Linear Motif (ELM) database, which is the most comprehensive, manually curated database of SLiMs, currently holds 2,092 experimentally validated human SLiM instances (Kumar *et al*, 2020). Most of these interactions have been characterized through low‐throughput experiments, as the properties that make SLiM‐based interactions suited for their physiological function make them difficult to capture experimentally by classical large‐scale PPI discovery methods. For example, the stringent washing steps in large‐scale AP‐MS protocols bias selections toward stronger binders. In contrast, the resolution of modern variants of Y2H is ~ 20 μM (Cluet *et al*, 2020), which overlaps with the affinity range of most motif classes. However, Y2H is limited to proteins that can translocate to the nucleus, are not toxic in yeast, and do not cause autoactivation (Dreze *et al*, 2010). Many SLiM‐based PPIs rely on additional binding sites present in the interacting proteins, which further complicates their identification (Ivarsson & Jemth, 2019; Bugge *et al*, 2020). Consequently, it is likely that the majority of SLiMs remain to be discovered (Tompa *et al*, 2014).

**Figure 1:**
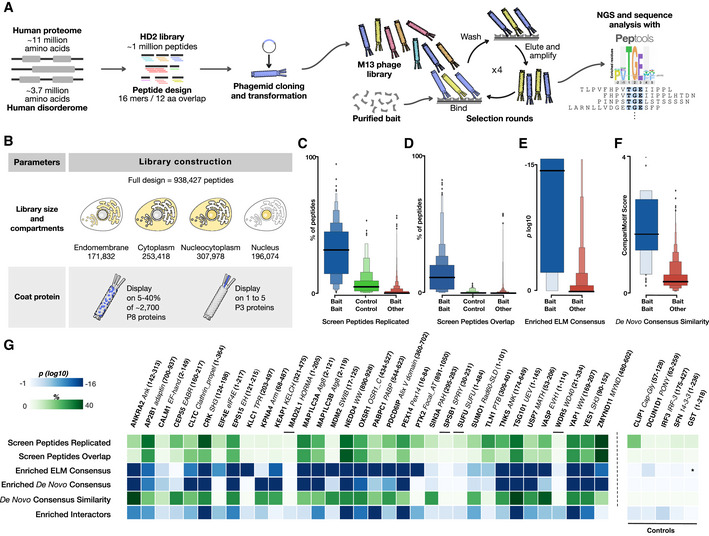
ProP‐PD workflow, library design and quality, and initial evaluation of selection results Schematic visualization of library design, cloning process, phage selection, and data analysis.Two main library parameters were explored: (i) comparing selection results from the whole HD2 library versus sublibraries grouped by subcellular localization, and (ii) the display of the HD2 peptide library design on phage proteins P8 (multivalent, HD2 P8) and P3 (monovalent, HD2 P3), respectively.Comparison of the percentage of peptides that are reproduced in pairwise comparisons between replicate selections for the same bait (blue), for the same control bait (green) and for different bait proteins (red).Comparison of the percentage of selected peptides that are overlapping in pairwise comparisons between replicate selections for the same bait (blue), for the same control bait (green), and for different bait proteins (red).Comparison of the log_10_ enrichment probability of the ELM defined motif consensus in peptides selected for the correct consensus‐binding bait (blue) and all other baits (red).Comparison of the CompariMotif similarity of the *de novo* SLiMFinder‐defined enriched motif in the overlapping and replicated peptides against the established ELM consensus for the bait (blue) and against all other ELM classes (red).Selection quality metrics split per bait. Data include metrics from panels (C) through (F). Enriched *de novo* consensus shows the *P*‐value of the SLiMFinder‐discovered enriched motif, and Enriched Interactors show the probability the selection returning the observed number of previously validated interactors for the bait by chance. Asterisk denotes no motif defined for the bait. Data for the panel are available in Dataset EV4. Data information: Boxen plots (C–F) are used to more accurately visualize the distribution of values. The central section has two blocks each containing 25% of the data split by the median (denoted by a dark black bar) and each additional block represents 50% of the data of the previous block. Sample sizes are (C) and (D)*:* n^bait‐bait^ = 358, n^control‐control^ = 156 and n^bait‐other^ = 23,276, (E): n^bait‐bait^ = 61 and n^bait‐other^ = 7,633, (F): n^bait‐bait^ = 40 and n^bait‐other^ = 1,560.

Proteomic peptide‐phage display (ProP‐PD) offers a large‐scale approach to simultaneously identify novel SLiM‐based PPIs and the binding motifs (Fig 1A) (Ivarsson *et al*, 2014; Davey *et al*, 2017). In ProP‐PD, a phage‐encoded peptide library is computationally designed to display the disordered regions of a target proteome. The designed peptides are displayed on the M13 phage that has a circular single‐stranded DNA (ssDNA) genome that is encapsulated by five coat proteins (Huang *et al*, 2012; Marvin *et al*, 2014). Approximately 2,700 copies of the major coat protein P8 cover the length of the phage, and five copies of the minor coat protein P3, which is necessary for infection, are presented at one end of the phage (Fig 1B). The approach is similar to combinatorial peptide‐phage display that has been extensively applied to identify SLiM specificity determinants (Teyra *et al*, 2020), but displaying designed sequences instead of randomized sequences. We have previously constructed a first‐generation human disorderome (HD1) (Davey *et al*, 2017) displayed on the major coat protein P8 and used it to identify interactors and binding sites for several proteins, including the docking interactions of the phosphatases PP2A (Wu *et al*, 2017), PP4 (Ueki *et al*, 2019), and calcineurin (Wigington *et al*, 2020). However, the HD1 library suffers from limitations that have hampered the exploitation of the full power of the approach, with a main limitation being a low coverage of the library design in the constructed phage library due to low quality of the oligonucleotide pool obtained from the commercial provider and suboptimal tiling of the IDRs (Davey *et al*, 2017). The field has also been limited by a lack of guidelines on how to design ProP‐PD experiments, postprocess the results, and attribute confidence to the selected peptides.

In this study, we present a novel resource for the interactomics community. We describe an optimized human disorderome library (HD2), an online toolkit for annotation and analysis of selected peptide ligands termed PepTools (http://slim.icr.ac.uk/tools/peptools/), and general guidelines on how to analyze the results. We evaluated the HD2 ProP‐PD library by using it in selections against a benchmarking set of 34 bait protein domains representing 30 distinct domain families with known motif‐mediated interaction partners listed in the ELM database (Kumar *et al*, 2020). We also screened against the HEAT repeat of importin subunit beta‐1 (KPNB1 HEAT), which is a challenging test case due to its typically low affinity for individual peptide ligands (Milles *et al*, 2015). Selections against the novel HD2 library captured 65 (19.3%) of the 337 known SLiM‐mediated interactions for the screened protein domains, which is twice the recall of SLiM‐based interactions as compared to the recall of Y2H and MS based screens. We uncovered 2,161 potential SLiM‐mediated interactions and defined the binding sites of these interactions at amino acid resolution. Biophysical characterization demonstrated that the selections capture interactions in a broad affinity span, ranging from low nanomolar to millimolar range. Using importin subunit alpha‐3 (KPNA4) we validated the functional relevance of novel interactions. We further systematically tested parameters to define the optimal analysis setup by examining the use of cell compartment‐specific sublibraries, and the display on the minor coat protein P3 instead of the major coat protein P8. Finally, we explored the effects of phosphorylation or disease‐related mutations on the interactions, thus highlighting the advantage of simultaneous PPI screening and binding site identification. The approach outlined here is generally applicable and will be of great value when exploring interactions involving the IDRs of the human proteome.

## Results

### ProP‐PD library design, construction, and quality control

We designed a phage‐encoded library of peptides representing the IDRs of the intracellular human proteome (Fig 1A, Dataset EV1). These disordered regions were tiled as 16‐amino acid‐long peptides that are overlapped by 12 amino acids. The library contains 938,427 peptides from 16,969 proteins and covers approximately one‐third of the proteome tiled with overlapping peptides. An interactive website to explore the full library design is available at http://slim.icr.ac.uk/phage_libraries/human/proteins.html. The library was subdivided into different, partially overlapping, pools based on the cellular localization of the peptide‐containing proteins (cytoplasmic, endomembrane, cytoplasmic and nuclear, and nuclear based on localization annotation; Fig 1B) to allow for compartment‐specific sampling of the interaction space. The point of subdividing the library into pools based on subcellular localization is to reduce the number of competing interactions.

The sequences were displayed using an M13 phage system where fusion proteins of the designed peptides and a coat protein are encoded by a phagemid, and a M13KO7 helper phage provides all genes necessary for phage infection, replication, assembly, and budding (Ledsgaard *et al*, 2018). Fusion of the peptides to the P8 protein results in the display of peptides on 5–40% of the ~ 2,700 copies of the P8 protein on each phage (Fig 1B) (Malik *et al*, 1996). We also generated a version of the HD2 library displayed on the minor coat protein P3 (HD2 P3; Fig 1B), which results in monovalent display. Next‐generation sequencing (NGS) of the phage libraries confirmed that ~ 90% of the designed peptide sequences were present in the constructed libraries, and the extrapolated library coverage percentage surpassed 95% (Dataset EV1, Appendix Fig S1). As each amino acid of the IDRs is covered by at least two overlapping peptides, this design ensures full coverage of the human IDRs by the library. We thus confirmed that the constructed phage libraries have high coverage and are of high quality.

### Phage selections and initial evaluation of selection results

We established a benchmarking set of 34 SLiM‐binding domains from 30 domain families (Table 1, Dataset EV2). The selected bait domains were chosen to represent the diversity of motif types recognized by motif‐binding pockets (Table 1, Appendix Fig S2, Dataset EV3, http://slim.icr.ac.uk/data/proppd_hd2_pilot). In addition, we included the HEAT domain of KPNB1 as a challenging test case based on its typically low ligand affinity (Milles *et al*, 2015). A set of protein domains not expected to bind to the library peptides were chosen as negative controls, namely the phospho‐peptide‐binding proteins 14‐3‐3 protein sigma (SFN 14‐3‐3) (Yaffe *et al*, 1997) and interferon regulatory factor 3 (IRF3 IRF‐3) (Liu *et al*, 2015), the *N*‐acetyl‐peptide‐binding PONY domain of the DCN1‐like protein 1 (DCUN1D1 PONY) (Scott *et al*, 2011), and the C‐terminal‐binding Cap‐Gly domain of CAP‐Gly domain‐containing linker protein 1 (CLIP1 Cap‐Gly) (Kumar *et al*, 2020). As the libraries described here do not display free N‐terminal or C‐terminal residues, and no post‐translational modifications are introduced, these domains should represent valid negative controls. GST was used as an additional negative control as all bait proteins were GST‐tagged.

**Table 1:** Overview of the baits and the outcome of the ProP‐PD selections

**Gene**	**Domain**	**Motifs found**	**Motifs in library**	**Observed motif**	**Expected motif**
ANKRA2	Ank	2	4	[LMP]xLPx[FIL]	**PxLPx[IL]** x{1,3}[VLF]
AP2B1	Adaptin	2	8	[FW]xx[AFLP]	[DE]x{1,2} **Fxx[FL]** xxxR
CALM1	EF‐hand	0	19	WxxL	[ACLIVTM]xx[ILVMFCT]Qxxx[RK]
CEP55	EABR	1	3	PPxxxY	AxG **PPx{2,3}Y**
CLTC	Clathrin‐propeller	0	9	LIx[FW]	**L[IVLMF]x[IVLMF]** [DE]
CRK	SH3	2	11	Px[LV]Px[KR]	**PxxPx[KR]**
EIF4E	eIF4E	2	6	–	YxxxxL[VILMF]
EPS15	EH	10	37	NPF	**NPF**
KEAP1	KELCH	1	7	TGE	[DNS]x[DES] **[TNS]GE**
KLC1	TPR	0	8	–	[LMTAFSRI]xW[DE]
KPNA4	Arm	0	18	KRxxx[DES]	Polybasic
KPNB1	HEAT	0	2	[AILPV][FY]xF	**FxF** G
MAD2L1	HORMA	0	2	–	[KR][IV][LV]xxxxxP
MAP1LC3A	Atg8	5	14	[FWY]xx[ILV]	[EDST]x{0,2} **[WFY]xx[ILV]**
MAP1LC3B	Atg8	3	15	[FHWY]xx[ILV]	[EDST]x{0,2} **[WFYxx[ILV]**
MDM2	SWIB	3	5	FxxxWxxL	**FxxxW** xxx[VIL]
NEDD4	WW4	2	8	[LP]PxY	**PPxY**
OXSR1	OSR1‐C	4	6	RFx[IV]	**RFx[IV]**
PABPC1	PABP	10	19	AxxF[VY]P	[LFP][NS][PIVTAFL]x **Axx[FY]x[PYLF]**
PDCD6IP	Alix‐V‐domain	0	0	YPxL	[LM] **YPx[LI]**
PEX14	Pex14	0	9	[FLM]xxxW	**Fxxx[WF]**
PTK2	Focal‐AT	2	5	–	[LV] [DE] x [LM] [LM]xxL
SIN3A	PAH	1	6	[FILMVW]xxL[LV]	[FHYM]xA[AV]x[VAC]L[MV]x[MI]
SPSB1	SPRY	0	1	–	[ED][LIV]NNN
SUFU	SUFU	0	2	–	[SV][CY]GH[LIF][LAST][GAIV].
SUMO1	Rad60‐SLD	6	29	[IV]DLxxD	[VILPTM] **[VIL][DESTVILMA][VIL]**
TLN1	PTB	0	13	Wxx[NS]x[IL]	NPx[FY]
TNKS	Ank	2	16	Rxx[AP]xG	**R** xx **[PGAV]** [DEIP] **G**
TSG101	UEV	1	10	[AP][ST]AP	**P[TS]AP**
USP7	MATH	1	9	[AP][GS]xS	**[PA]xxS**
VASP	EVH1	2	11	[FW]PxP[LP]	**[FYWL]PxPP**
WDR5	WD40	0	11	–	[SCA]AR[STCA]
YAP1	WW1	4	9	[LP]PxY	**PPxY**
YES1	SH3	0	5	RxLPxxP	**[RKY]xxPxxP**
ZMYND11	MYND	0	2	[MP]Px[LY]	**PxL** xP
GST	GST	–	–	–	–
DCUN1D1	PONY	0	2	–	^M[MIL]x[MIL]
SFN	14‐3‐3	0	58	–	LxIS
IRF3	FHA	0	3	–	Rxx[ST]xP
CLIP1	Cap‐Gly	0	4	–	xW[RK][DE]GCY$;[ED]x{0,2}[ED]x{0,2}[EDQ]x{0,1}[YF]$

Overview of the bait constructs screen in the current study, the number of validated motifs discovered in selection for each bait, the number of validated motifs present in the HD2 library, the enriched motif consensus in the peptides selected for each bait, and the expected consensus for each bait. Gray shaded area indicates baits used as negative controls. The bold and underlined characters indicate matches between the motifs reported in ELM and the motif generated based on ProP‐PD results. Sequence logos of the observed and expected motifs are available for comparison at http://slim.icr.ac.uk/data/proppd_hd2_pilot.

The HD2 libraries, the HD1 library (displayed on P8), and a combinatorial peptide phage display library with high complexity (displayed on P8, estimated 10^10^ diversity) (Ilari *et al*, 2015) were used in triplicate selections against the immobilized bait proteins for four rounds of phage selections. The peptide‐coding regions of the binding‐enriched phage pools were barcoded and analyzed by NGS (Appendix Fig S3). The peptide sequences were mapped to the human proteome with PepTools (http://slim.icr.ac.uk/tools/peptools/), our novel web‐based tool developed for the annotation of protein regions built on the annotation framework of the PSSMSearch tool (Krystkowiak *et al*, 2018) (Dataset EV4: http://slim.icr.ac.uk/data/proppd_hd2_pilot). Next, we analyzed the selected peptides for each bait to understand the ability of the ProP‐PD approach to specifically and reproducibly enrich for binders.

We found an enrichment of replicated peptides in selections against the same bait proteins, as expected for successful selections (Fig 1C). Overlapping peptides were more frequently found in selections for the same bait as compared to unrelated screens (Fig 1D). Moreover, the expected ELM consensus for a bait was often enriched in identified peptides selected for that bait (Fig 1E), and the consensus motif discovered *de novo* based on the identified peptides matched the key residues of the expected ELM consensus for the bait (Fig 1F and Dataset EV3, http://slim.icr.ac.uk/data/proppd_hd2_pilot). Replicated peptides, overlapping peptides, and enriched binding determinants are hence strong indicators of a successful selection. We further analyzed the results on the bait protein level (Fig 1G), and found that only four of the bait proteins from the benchmarking set had selection quality statistics that were similar to the negative controls, indicating little or no enrichment for specific binders (MAD2L1, SPSB1, SUFU, and WDR5). The low enrichment of ligands observed for these domains with well‐characterized motif‐binding preferences might relate to protein quality issues (including for example incompatibility with the immobilization method) (Kumar *et al*, 2020).

### Benchmarking of metrics for ranking of ProP‐PD results

**Figure 2:**
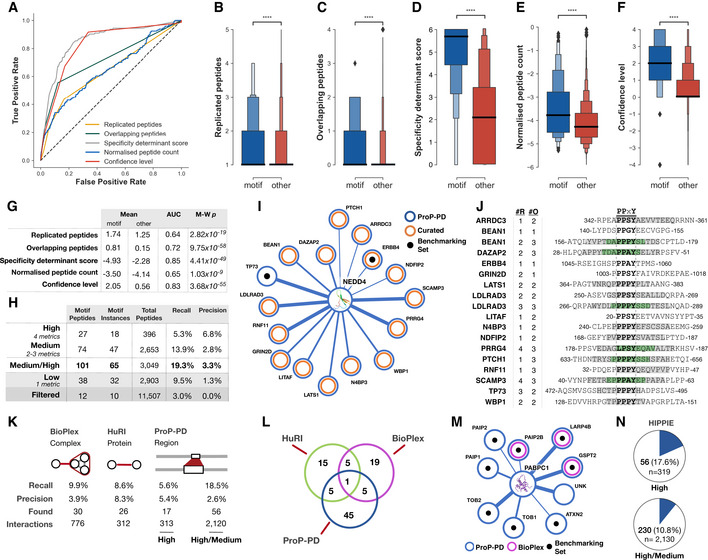
Benchmarking of metrics for ranking of ProP‐PD results, evaluation of motif rediscovery, and interactome to interactome comparisons of results ROC curves of the metrics used to assign confidence levels.Boxen plot of the number of replicated peptides for motif‐containing peptides from the benchmarking datasets (blue) compared to all other selected peptides (red).As panel (B), showing overlapping peptides.As panel (B), showing the PSSM‐derived specificity determinant score defining the similarity of the selected peptides to the SLiMFinder‐discovered enriched motif. Score is log_10_ of the PSSMSearch PSSM probability.As panel (B), showing log_10_ of the normalized peptide count.As panel (B), showing the consensus confidence level defined based on the replicated peptides, overlapping peptides, specificity determinant match, and normalized peptide count.The predictive power, defined by the area under the ROC curve (AUC) and Mann–Whitney–Wilcoxon two‐sided test with Bonferroni correction *P*‐value (M‐W p), of the four confidence metrics and the consensus confidence level metric.Benchmarking statistics of the four consensus confidence levels and the high/medium confidence levels grouped. Recall calculated on motif instances against the benchmarking dataset of 337 motif instances. Precision calculated as the number of motif‐containing peptides over the number of peptides at given confidence level.Partial network of ProP‐PD‐derived high/medium interactors of the NEDD4 WW4. Shown interactions are annotated as WW domain ligands in the ELM resource (black) or curated from the literature (orange). Line thickness indicates the number of quality metrics fulfilled by the hit (4, 3, or 2).Peptides matching previously validated NEDD4 binding peptides from panel (I) annotated with the number of replicates (#R) and the overlapping peptides (#O; gray denotes two overlapping peptides for the region and green denotes three overlapping peptides).Interaction‐centric benchmarking metrics of the ProP‐PD, BioPlex, and HuRI based on the 302 unique motif‐mediated interactions for the 337 motif instances from the motif benchmarking dataset. Found is the number of motif‐mediated interactions from the benchmarking dataset that were rediscovered by each method, interactions are the total number of interactions returned by each method for the baits in the motif benchmarking dataset.Overlap of previously validated motif‐based PPIs (*N* = 302) in the ProP‐PD benchmarking dataset rediscovered by ProP‐PD, BioPlex, and HuRI.PABPC1 PPI network for proteins containing high/medium confidence peptides and annotated with BioPlex (magenta) interaction data. Edge width represents ProP‐PD confidence level. Black dots represent peptides that overlap with a known ELM instance. HuRI did not return any of these interactions.Overlap between the ProP‐PD interactions and interactions in the HIPPIE database. Data information: Boxen plots (B‐F) are used to more accurately visualize the distribution of values. The central section has two blocks each containing 25% of the data split by the median (denoted by a dark black bar) and each additional block represents 50% of the data of the previous block. Asterisks denote the likelihood of the null hypothesis that the distribution underlying each sample is the same using a Mann–Whitney *U* test (****P‐value = < 1.0 × 10^−4^). Sample sizes are n^motif^ = 144 and n^other^ = 18,679.

Next, we benchmarked the discriminatory power of several criteria for filtering and prioritization of the selected peptides to establish a robust protocol for data analysis. The data returned from successful ProP‐PD selections contain enriched bait‐binding peptides and noise introduced by spurious peptides identified because of the depth of the sequencing. We used four metrics to define peptide quality: (i) reproducible occurrence in replicate selections, (ii) identification of a region with overlapping peptide hits, (iii) the presence of a shared consensus motif, and (iv) strong enrichment as indicated by high NGS read counts (Fig 2A). We evaluated the discriminatory power of each of the metrics using a *ProP‐PD motif benchmarking dataset* (Dataset EV5; http://slim.icr.ac.uk/data/proppd_hd2_pilot) compiled from the ELM database and structures of SLiM‐domain complexes available in the Protein Data Bank (PDB). The benchmarking dataset contains 337 motif instances that have previously been reported to bind to the 34 benchmarking bait proteins and that are represented in the HD2 P8 library. We found, as expected, that peptides that were discovered through the HD2 P8 selections and overlapped with the benchmarking dataset were more frequently found in replicate selections (*P* = 2.82 × 10^−19^), identified with overlapping peptides (*P* = 9.75 × 10^−58^) and contained the *de novo* consensus established for the ProP‐PD‐derived peptides using SLiMFinder (*P* = 4.41 × 10^−49^; Fig 2B–D). Previously validated motif instances also had higher than average normalized peptide counts (*P* = 3.68 × 10^−9^; where normalization is based on the NGS counts observed for each peptide in a replicate selection against a given bait to the total NGS counts for the bait selection; Fig 2E, see also Appendix Fig S4A). The results support that the four metrics have predictive power in terms of discriminating genuine binding peptides from the non‐specific background binding events (Fig 2G). Cut‐off values were determined for each of the four metrics through receiver operating characteristic (ROC) curve analysis (Fig 2A). The resulting binary confidence criteria obtained for the individual metrics were combined for each peptide to create a single score termed “Confidence level” (Fig 2F). Peptides were classified into four categories based on their confidence level (“High” for a confidence level of 4, “Medium” for a confidence level of 2 or 3, “Low” for a confidence level of 1, and “Filtered” for all other peptides). As expected, we identified no or few medium/high confidence peptides for the negative control baits. One notable exception was the overlapping and replicated _1836‐_PSWLADIPPWVPKDRP_‐1851_ peptide from microtubule‐associated protein 1A (MAP1A) selected by the SFN 14‐3‐3. The aspartate side chain of the MAP1A1836–1851 peptide may mimic the negative charge of a phospho‐serine, as previously shown for other unphosphorylated 14‐3‐3‐binding peptides (Petosa *et al*, 1998; Wang *et al*, 1999; Ottmann *et al*, 2007; Teyra *et al*, 2020). We validated the interaction through fluorescence‐polarization (FP)‐based affinity determination (Appendix Fig S4B) and showed it binds with low affinity (*K*_I_ 355 μM; Dataset EV6). In a cellular setting, the low affinity SFN 14‐3‐3–MAP1A interaction is likely outcompeted by the large number of phosphorylated 14‐3‐3 ligands available in the cell (Gogl *et al*, 2021).

### ProP‐PD selections rediscover one‐fifth of known motifs as medium/high confidence ligands

We benchmarked the HD2 P8 results in terms of motif rediscovery using *recall* (the proportion of previously validated motifs found through phage selections against the bait) and *precision* (the proportion of peptides found that contain a motif previously reported to bind the bait). Peptides that met all four of the metrics represent the high confidence set (396 peptides), when compared to other confidence levels these peptides have lower recall of previously validated ligands but higher precision (Fig 2H). Hits fulfilling two or three of the metrics (2,653 peptides) represent a medium confidence set with higher recall but lower precision. The peptides that fulfilled only one of the metrics (2,903 peptides) were considered of low confidence. Finally, a large set of peptides (11,507) that fulfilled none of the criteria contained very few previously validated ligands (10). For a stringent analysis, we focus on the 3,049 peptides in the medium/high confidence bins (Dataset EV4: http://slim.icr.ac.uk/data/proppd_hd2_pilot). In total, 65 (19.3%) of the 337 previously validated motifs in the benchmarking set were found in the medium/high confidence dataset. Importantly, all peptides in the high confidence bin contain motifs that match the SLiMFinder motifs generated based on the ProP‐PD data (Dataset EV3; http://slim.icr.ac.uk/data/proppd_hd2_pilot), as motif match is one of the four criteria used for the binning. In the medium confidence set, the majority have a consensus motif (peptides matching ELM consensus 1,420; peptides matching SLiMFinder motif 2,112). Performing the same analysis of the HD1 P8 data generated for the same bait proteins resulted in only 34 previously validated motif instances among 1,944 peptides, supporting that the HD2 P8 library is an improved resource for discovery of motif‐based interactions.

### HD2 P8 selections generate large‐scale data with similar quality to other interactomics studies

On the protein level, the median number of PPIs identified by ProP‐PD per bait is 27, spanning from baits such as USP7 MATH and YAP1 WW1 domains for which more than 200 PPIs were found, to baits such as PABC1 PABP and WDR5 WD domain for which less than 10 PPIs were found. We assessed the ability of HD2 P8 phage selections to identify SLiM‐based interactions by comparing the PPI data with large‐scale interactomics datasets, namely HuRI (Luck *et al*, 2020) and BioPlex (Huttlin *et al*, 2021) (Fig 2K–N). As a reference set, we used the *ProP‐PD motif benchmarking dataset* (Dataset EV5) where 302 PPIs were annotated for the 337 motif instances. The medium/high confidence HD2 P8 data have twice the recall (the proportion of PPIs that have been rediscovered) of BioPlex and HuRI on the motif‐based interactions set, thus demonstrating the efficiency of ProP‐PD in finding SLiM‐based interactions over the compared methods. Conversely, the precision of the medium/high confidence ProP‐PD dataset (the proportion of rediscovered PPIs among all PPIs found) is the lowest of the three studies. However, the large number of peptides that contain the correct motif consensus for the bait domain (Dataset EV4) suggests that the lower precision of the HD2 P8 data may reflect the discovery of a large number of additional SLiM‐based interactions. Notably, the intersections between the three methods were low (Fig 2L) suggesting that they sample different parts of the interactome, as showcased for the poly(A)‐binding protein (PABP) domain of polyadenylate‐binding protein 1 (PABPC1; Fig 2M). Finally, many of the interactions discovered by HD2 P8 selections have support from other studies based on the information listed in the HIPPIE database (Fig 2N), which integrates and scores information on human PPIs from 10 source databases including BioGRID, MINT, HPRD, and IntAct (Alanis‐Lobato *et al*, 2017). In conclusion, we find that HD2 ProP‐PD selections generate large‐scale data on motif‐mediated interactions with similar quality to other large‐scale studies while also providing amino acid resolution of the binding sites.

### Gene Ontology enrichment analysis of the ProP‐PD‐based interactome

A classical Gene Ontology (GO) term enrichment analysis was performed on the complete high/medium confidence interactome of each bait (Dataset EV7). In several cases, the GO term corresponding to the expected function or localization of the bait was significantly enriched in the interacting peptide‐containing proteins. For example, the localization of the EPS15 (clathrin‐coated pit *P* = 1.56 × 10^−8^), MAP1LC3A (autophagosome *P* = 1.57 × 10^−5^), and PABPC1 (polysome *P* = 5.85 × 10^−6^) was correctly identified as the most significant GO localization terms. We saw similar examples for the functional GO terms of ANKRA2 (histone deacetylase activity [H3‐K14 specific] *P* = 4.16 × 10^−6^), CRK (SH3 domain binding *P* = 5.09 × 10^−10^), SUMO1 (SUMO transferase activity *P* = 1.93 × 10^−10^), and the biological processes of EIF4E (regulation of translation *P* = 4.20 × 10^−8^), KPNB1 (protein import into nucleus *P* = 6.40 × 10^−7^) and PABPC1 (regulation of translation *P* = 4.28 × 1^−10^). Next, we performed a similar enrichment analysis directly on the ProP‐PD interactomes by comparing them with the aggregated human interaction data from HIPPIE to discover interactomes that had significant overlap (Dataset EV7). We observed that the ProP‐PD‐derived interactomes for 19 bait proteins had significant overlaps with the previously discovered interactions for the baits. Of these, the six most significantly overlapping interactomes were found for CRK (*P* = 1.71 × 10^−20^), EIF4E (*P* = 7.4 × 10^−9^), EPS15 (*P* = 2.77 × 10^−13^), PABPC1 (*P* = 1.01 × 10^−12^), PTK2 (*P* = 8.86 × 10^−5^), and TNKS (*P* = 7.08 × 10^−8^).

Finally, we performed a shared GO terms analysis on the ProP‐PD interactomes (Dataset EV7). The analysis compared the overlapping GO annotations of the bait protein with the peptide‐containing protein and calculated the likelihood that each GO term was shared by chance. The most significant terms for each peptide in all three GO classes were used to benchmark the approach and revealed a significant enrichment of shared GO terms for validated motifs (Appendix Fig S5). In total, the analysis revealed that 286 of the interactions with a high/medium confidence shared significant GO terms with their bait (*P* < 1 × 10^−4^): 95 for Localization; 206 for Biological Process, and 97 for Molecular Function terms (Dataset EV4). These data, when considered with the experimental metrics, provide valuable information for peptide prioritization for further validation.

### ProP‐PD results range from extensive rediscovery of known binding peptides to discovery of alternative motifs depending on the bait proteins

For some bait proteins, there was an extensive overlap between the HD2 P8 data and previously reported binding motifs. For example, for the PABP domain of PABPC1 (Fig 2M) 18 medium/high confidence peptides were identified of which all but two overlapped with previously validated motifs. This represented 10 (53%) of the 19 PABP‐binding peptides in the ProP‐PD motif benchmarking dataset (Dataset EV5). The remaining two peptides mapped to an overlapping 12 residue stretch found in the RING finger protein unkempt homolog (UNK; _496‐_GM**NA**N**A**LP**F**Y**P**T_‐507_; bold residue denote residues matching the expected motif consensus for this bait), which may represent a novel PABPC1 ligand. Alternatively, it may be a ligand for the homologous PABP domain from HECT E3 ubiquitin‐protein ligases UBR5, which recognizes similar motifs and is functionally more closely related to UNK (Lim *et al*, 2006).

For other bait proteins, the selections returned a large number of medium/high confidence hits that matched the expected consensus motif, but with no, or very limited, overlap with the benchmarking set (Dataset EV5). This may in part be explained by the lack of curation of the relevant motif literature. For example, we used the fourth WW domain of E3 ubiquitin‐protein ligase NEDD4 (NEDD4 WW4), and the first WW domain of transcriptional coactivator YAP1 (YAP1 WW1) as representative cases of PPxY‐binding WW domains (Sudol *et al*, 1995; Ingham *et al*, 2004). We found 426 unique medium/high confidence peptides. Of these, only five motifs were found in the ProP‐PD benchmarking set (Dataset EV5). To more thoroughly evaluate the quality of the results, we surveyed the WW domain literature and compiled a set of 124 experimentally validated PPxY motif instances (Dataset EV8). The NEDD4 WW4 selections identified 34 of the curated WW domain‐binding instances as medium/high confidence ligands, of which 19 were previously reported as NEDD4 binders (Fig 2I and J). The selections against YAP1 WW1 identified 40 of the reported motif WW‐binding motifs (12 YAP1 ligands). The recall of real binders is thus higher than the conservative estimate provided using the ProP‐PD motif benchmarking dataset.

Finally, for some baits, we found enrichments of peptides that did not match the expected binding preferences (Table 1, Dataset EV3, http://slim.icr.ac.uk/data/proppd_hd2_pilot). For example, calmodulin (CaM) is a ubiquitous calcium sensor that binds SLiMs upon Ca^2+^ activation. CaM‐binding motifs have high helical propensity, net positive charge, and two anchor residues, and are classified into different groups based on the distance of the anchor residues (e.g., 1–5–10 and 1–8–14) (Yap *et al*, 2000; Tidow & Nissen, 2013). Variant motifs such as the 1–4‐(8/9/10) motif have also been described (Patel *et al*, 2017). We performed selections against CaM in the presence and absence of 1 mM Ca^2+^. We found 15 medium/high confidence peptides under both conditions, and an additional set of 141 peptides only under the Ca^2+^ condition, suggesting that Ca^2+^ primed the protein for binding (Dataset EV9). The consensus motifs generated based on peptides selected under the different conditions were similar (no Ca^2+^ WxxL; 1 mM Ca^2+^ [FHW]xx[ILV]) and resembled the less explored 1–4‐(8/9/10) motif (Patel *et al*, 2017). Ligands with the longer classical CaM‐binding motifs were not captured, likely due to the minimum length of the motifs exceeding the designed peptide length of the ProP‐PD library.

### ProP‐PD selections capture interactions with a broad range of affinities

**Figure 3:**
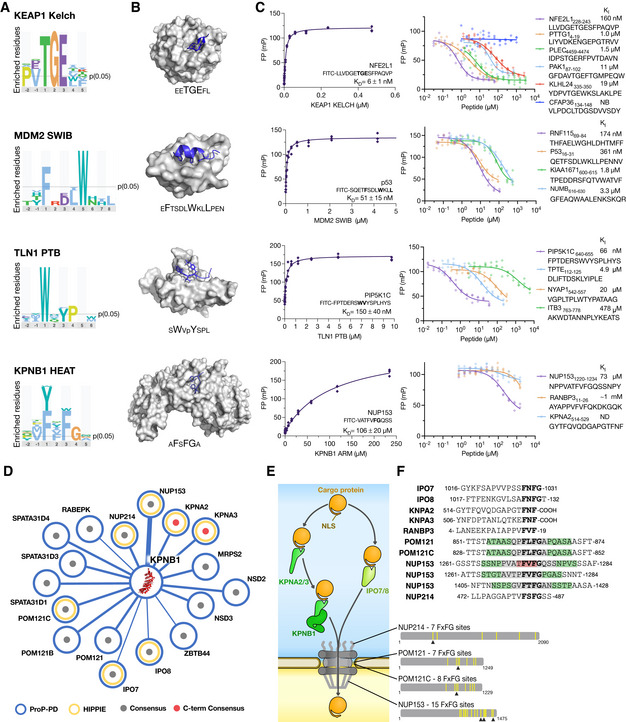
ProP‐PD selections capture interactions with a broad range of affinities Sequence logos for the indicated bait proteins generated by PepTools using the medium/high confidence set of ligands.Structures of KEAP1 Kelch, MDM2 SWIB, TLN1 PTB, and KPNB1 HEAT with the sequences of the bound peptides indicated (PDB codes 2FLU, 1YCR, 2G35, and 1O6O). Larger letters indicate residues that make up the consensus motifs.FP affinity determinations. Affinities were measured by first determining the *K*_D_ value of FITC‐labeled probe peptides, and then determining the affinities for unlabeled peptides through competition experiments. All experiments were performed in triplicates (source data are provided). See Dataset EV6 for more details.Partial network of KPNB1 ligands. Edge thickness reflects the confidence levels. Gray dot indicates that the peptide has a FxFG motif, red dot indicates FxF‐coo^−^ motif. Previously known ligands reported in the HIPPIE database are indicated by yellow circle.Schematic of KPNB1's role in nuclear transport together with identified FxF(G/‐coo^−^) containing ligands. The multitude of FxFG repeats in NUP213, POM121/C, and NUP153 are indicated by yellow bars. Arrowheads indicate the KPNB1 binding sites identified in HD2 selections.Sequence alignment of identified KPNB1‐binding peptides from proteins involved in nuclear transport (gray, two overlapping peptides for the region; green, three overlapping peptides; red, four overlapping peptides).

Next, we set out to understand the affinity range of the interactions captured by ProP‐PD selections. We selected a set of representative peptides expected to bind their baits with a wide range of binding affinities and quantified their binding using FP‐based measurements (Fig 3).

The kelch‐like ECH‐associated protein (KEAP1) is a substrate adaptor of the BTB‐CUL3‐RBX1 E3 ubiquitin ligase complex. The KEAP1 Kelch domain binds to a short negatively charged degradation motif (Table 1, Fig 3A and B, http://slim.icr.ac.uk/data/proppd_hd2_pilot) called a degron that targets substrates, such as the nuclear factor erythroid 2‐related factor 2 (NFE2L2), for ubiquitination and degradation (Cullinan *et al*, 2004). The HD2 P8 selections returned 29 medium/high confidence KEAP1 Kelch ligands from 23 proteins (Dataset EV4) including its known substrate the endoplasmic reticulum membrane sensor NFE2L1 (Cullinan *et al*, 2004). Affinities were determined for the four TGE containing peptides from NFE2L1_228–243_, PAK1_88–103_, KLHL24_335–350_, and PLEC_4459–4474_, which revealed that the NFE2L1_228–243_ peptide displayed higher affinity (*K*_I_ 0.16 µM; Fig 3C, Dataset EV6) as compared to the other peptides (1.5–19 µM *K*_I_ values), which may contribute to the high specificity of the protein for its primary substrate. In addition, we determined the affinity for a peptide from securin (PTTG1; identified in the selection against the nuclear/cytoplasm sublibrary described later) that has NGE variant of the TGE motif, which also matches the KEAP1 degron motif consensus. The NGE containing peptide from PTTG1_4–19_ bound to KEAP1 with similar affinity (1 µM *K* *_I_* value), to the TGE containing peptides (Fig 3C). Thus, peptides with variations in a given motif, may represent high‐affinity ligands. Moreover, we tested binding of a set of peptides lacking the obvious KEAP1‐binding motif and confirmed that they either did not bind at all (CFAP36_134–148_; Fig 3C) or bound with low affinity (Appendix Fig S4, Dataset EV6).

The E3 ubiquitin‐protein ligase MDM2 mediates ubiquitination of cellular tumor suppressor p53 (p53). We identified 14 medium/high confidence MDM2 ligands from 12 proteins, including the known MDM2‐binding peptide of p53 (Kussie *et al*, 1996). We determined the affinities for the p53_16–31_ peptide (*K*_I_ of 0.36 µM), together with three novel peptide ligands from the protein numb homolog (NUMB_615–630_), the E3 ubiquitin‐protein ligase RNF115 (RNF115_69–84_), and the uncharacterized protein KIAA1671 (KIAA1671_600–615_; Fig 3C). NUMB is a known substrate of MDM2, although this interaction site has not been experimentally validated previously (Juven‐Gershon *et al*, 1998; Sczaniecka *et al*, 2012; Colaluca *et al*, 2018). RNF115 ubiquitinates p53 in lung adenocarcinoma (Luo *et al*, 2020), supporting a functional interplay between the two proteins. KIAA1671 is in contrast a poorly studied and largely unstructured protein. The affinities of MDM2 for the newly discovered ligands ranged from 0.17 µM for the RNF115_69–84_ to 3.3 µM for the NUMB_616–631_ peptide, and they are thus in a similar affinity range as the previously known binder p53.

Talin‐1 (TLN1) is a cytoplasmic adapter protein necessary for integrin‐mediated cell adhesion through an interaction between the TLN1 phosphotyrosine binding (PTB)‐like domain and an NPxY motif found in the cytoplasmic tails of integrins (Legate & Fässler, 2009). Contrary to classical PTB domains, the TLN1 PTB domain lacks the basic residues required for recognition of the phosphorylated form of the NPxY motif, and its interaction with integrin is negatively regulated by phosphorylation (Calderwood *et al*, 2002; Anthis *et al*, 2009). We found 28 medium/high confidence TLN1 PTB‐binding peptides from 20 proteins (Dataset EV4), of which only one peptide contained the expected NPxY motif. Instead, the dataset was enriched with ligands with a tryptophan‐containing motif (Fig 3A). We selected three ligands for affinity measurements: the _542‐_VGPLTPL**W**TY**P**ATAAG_‐557_ peptide from the neuronal tyrosine‐phosphorylated phosphoinositide‐3‐kinase adapter 1 (NYAP1_542–557_; Dataset EV9), the _640‐_FPTDERS**W**VY**S**PLHYS_‐655_ peptide from the phosphatidylinositol 4‐phosphate 5‐kinase type‐1 gamma (PIP5K1C_640–655_), and the _94‐_DLIFTDSKLYIPLE_‐107_ peptide from the phosphatidylinositol 3,4,5‐trisphosphate 3‐phosphatase TPTE2 (TPTE2_94–107_). Of these, the interaction between PIP5K1C_647–652_ and TLN1 PTB is important for targeting PIP5K1C to focal adhesions (Di Paolo *et al*, 2002). We added the _763‐_AKWDTAN**NP**L**Y**KEATS_‐778_ peptide from integrin beta‐3 (ITB3_763–778_), which is a known TLN1 ligand (Garcia‐Alvarez *et al*, 2003) but not found among our results. While the phage‐derived peptides bound with affinities in the nano to micromolar range (Fig 3C, Dataset EV6), the ITB3_763–778_ peptide bound weakly (*K*_I_ of 0.5 mM), which provides an explanation for the absence of the peptide in the phage selection result.

**Figure 4:**
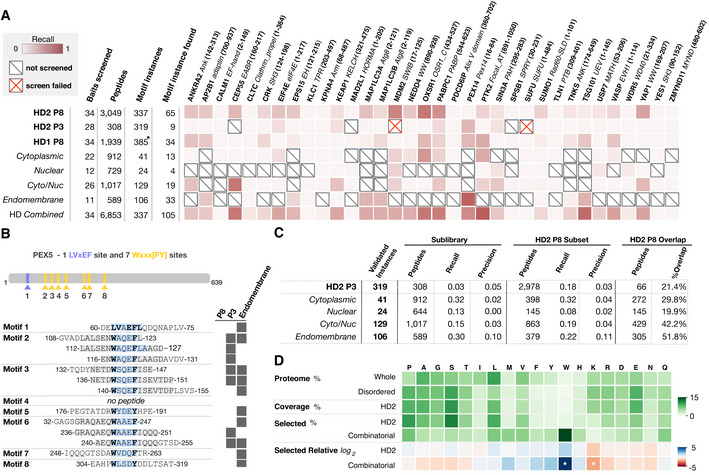
Library design parameters can influence data quality Per bait comparison of the proportion of findable motifs in the ProP‐PD motif benchmarking dataset found by each library.Overview of PEX14‐binding peptides in PEX5 returned from different libraries (motif region highlighted in light blue, motif residues in bold).Summary statistics of the data in panel (A) comparing the recall and precision of the selections against the HD2 P8 library and sublibraries or the HD2 P3 library. HD2 P8 recall is calculated on the subset of motif instances that are present in the compared library.Amino acid frequency (green color) in (i) the human proteome, (ii) the predicted IDRs, (iii) the HD2 library design, (iv) the binding enriched phage pools from selections against the HD2 P8 library, and (v) the combinatorial peptide phage display. The log_2_ of the relative amino acid frequencies of HD2 P8 and combinatorial peptide phage display versus the amino acid frequencies of predicted IDRs are shown in a gradient from blue to red. Note the significant enrichment of tryptophan and the depletion of lysine in the data from combinatorial peptide phage display selections (*z*‐score > 2 indicated by white asterisk) but not the ProP‐PD results. Source data are available online for this figure.

KPNB1 plays a central role in nuclear protein import (Fig 3E), and its HEAT repeat engages in low‐affinity interactions with FG repeats in proteins of the nucleoporin family (Hough *et al*, 2015; Raveh *et al*, 2016). The selections against KPNB1 successfully captured 23 [FWY]x[FW]G containing unique peptides found in 15 proteins (Fig 3D; Dataset EV9), of which 46% are found in known KPNB1 interactors (Fig 3E and F). In addition, there were two peptides containing FxF‐coo^–^ motifs. We selected three peptides for affinity determinations, the FxFG containing nuclear pore NUP153_1120–1134_, the C‐terminal motif of KPNA2_514–529_, and the RANBP3_4–19_ peptide. We found that NUP153_1120–1134_ bound with a relatively high affinity for a KPNB1 interaction (70 µM *K*_I_) and that the C‐terminal peptide of KPNA2 bound with low affinity (1 mM; Dataset EV6) demonstrating the large span of affinities that is recognized by KPNB1 and that can be captured through P8 HD2 selections.

Based on the affinity measurements, we evaluated whether the NGS counts of peptides in a selection correlated with their binding affinities, but found no clear trend (Appendix Fig S6). Factors such as minor biases in library composition and PCR amplification may contribute to confound affinity ranking based on NGS counts. Consequently, the method returns data that are qualitative, discriminating binders from nonbinders by enriching genuine biophysical binders from a library of almost a million peptides; however, it is not quantitative, as it cannot discriminate between small differences in affinity between binders.

### Library design parameters can influence data quality

In order to test the effect of the displaying coat protein and the library size of a ProP‐PD library on selection quality, we compared the results of the selections against different libraries using the ProP‐PD benchmarking set (Fig 4). First, we evaluated how monovalent display on the minor coat protein P3 affected the outcome of selections. The HD2 P3 selections were generally less successful than HD2 P8 selections based on the recall of ligands suggesting that the high avidity offered by the P8 display improves the selection (Fig 4A). However, there were exceptions to the rules, such as the N‐terminal domain of the peroxisomal membrane protein PEX14 for which the HD2 P8 selections failed to return known binders, but the HD2 P3 selections returned 3 out of 8 known motifs (Fig 4B; Dataset EV9). The HD2 P3 selection data can thus be used to complement the HD2 P8 selection results for certain bait proteins.

We further explored whether the use of compartment‐specific sublibraries (e.g., endomembrane, nucleus, cytoplasm; Fig 1B) would lead to a higher recovery of previously validated hits. We reasoned that limiting the search space to proteins that are found in the same cellular compartment as the bait would enrich for biologically relevant interactors. However, both the recall and the precision of HD2 P8 selections were on average at least as good as for the sublibrary selections, thus suggesting that there is no need to use compartment‐specific sublibraries for most bait proteins (Fig 4A and C). There were however some exceptions, in particular related to the results from the endomembrane sublibrary selections. Again, the difference was mainly due to the results of PEX14. Six out of the eight known PEX14‐binding motifs in the peroxisomal targeting signal 1 receptor (PEX5) (Neuhaus *et al*, 2014) were found through selection against the endomembrane sublibrary (Fig 4B). We conclude that the HD2 library in general is the most straightforward choice for most bait proteins but that compartment‐specific sublibraries may provide an advantage for some baits.

We further evaluate the reproducibility of the method (Fig 4C). Of the 3,049 high/medium confidence HD2 P8 peptides identified, 1,008 (33.1%) were confirmed by selection against either a HD2 P8 sublibrary or the HD2 P3 library. In total, 1,050 peptides were reproducibly found as medium/high confidence ligands in two or more datasets. This corresponds to 777 motif‐based PPIs of which 149 have been previously observed by complementary PPI discovery methods (Dataset EV9).

We finally compared the HD2 P8 data to the results of the combinatorial phage display selections. We found as previously reported (Luck & Trave, 2011) that the selection against the combinatorial phage library gave a strong bias for tryptophan‐rich peptides (Fig 4D). The ProP‐PD selection results were more similar to the proteomic frequencies of amino acids apart from minor shifts in the frequencies of tryptophan (enriched) and lysine (depleted). The HD2 library design thus largely circumvents the issue with selection of overly hydrophobic peptide sequences by combinatorial peptide phage display selection.

### From binding to function: identified KPNA4‐binding peptides are functional NLSs

**Figure 5:**
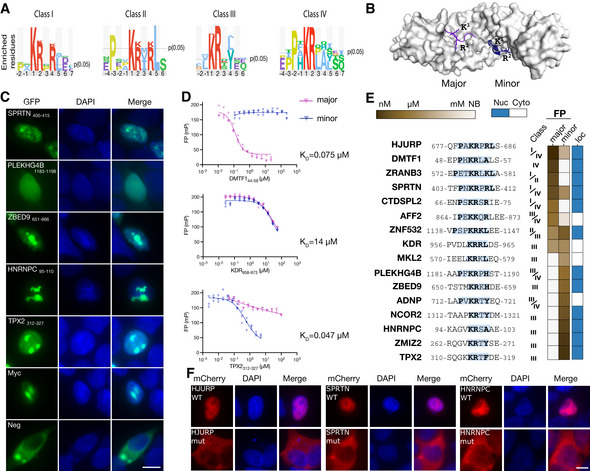
KPNA4‐binding peptides are functional NLSs Sequence logos of four different NLS classes binding to KPNA4 generated using PepTools.Structure of KPNA2 (PDB:1PJN, minor groove peptide PDB:3ZIP) with ligands bound to the major (purple) and minor groove (blue).Representative cellular localization experiment. HEK293 cells were transiently transfected with the NLS sensor and fixed 36 h after transfection, and imaged using epifluorescence microscopy. The nucleus was stained with DAPI. (*n* = 3, independent experiments; the scale bar indicates 10 μm).FP competition experiments using FITC‐Myc_320–328_ as a probe for the major groove (blue) or FITC‐NCOR2_1307–1322_ as a probe for the minor groove and competing with unlabeled DMTF1_44–59_, KDR_958–973_ and TPX2_312–327_ peptides. (*n* = 3, technical replicates, shown are individual data points. Source data are provided).Sequences of tested NLSs together with the outcome of the affinity measurement through FP and localization of the GFP‐tagged peptides (see Appendix Fig S8 for details).Mutational analysis of identified NLSs in the context of full‐length proteins using mCherry‐tagged HJURP, SPRTN, and HNRNPC. The scale bar indicates 10 μm. Source data are available online for this figure.

To take the validation of the results from binding to function we turned to KPNA4, an importin family member that binds to nuclear localization signals (NLSs) and transports cargo through the nuclear pore complex (NPC) by the classical nuclear import pathway (Fig 5). Like other importins, KPNA4 has two distinct NLS‐binding pockets on the surface of an armadillo (ARM) repeat fold (major pocket, ARM 2–4; minor pocket, ARM 6–8; Fig 5B) (Smith *et al*, 2018). Bipartite NLSs are usually 17–19 amino acids long and engage both pockets. Monopartite NLSs are short basic stretches, divided into five classes (Kosugi *et al*, 2009): class I KR[KR]R or K[KR]RK, class II [PR]xxKR[^DE][KR] (where ^ indicates “not”), class III KRx[WFY]xxAF, class IV [RP]xxKR[KR][^DE], and class V LGKR[KR][WFY]. The class I and class II motifs preferentially interact with the major pocket (Kosugi *et al*, 2009). In contrast, class III and class IV NLSs preferentially bind to the minor pocket. Using KPNA4 as a bait against the HD2 P8 library we identified 33 peptides found in 32 proteins. Of the 26 proteins for which information was available, 22 proteins were annotated as having nuclear or nucleolar localization in the UniProt database. To gain additional information we combined the data generated for KPNA4 by screening against the complete P8 HD2 library, with the results generated using two relevant sublibraries (the cytoplasmic and nuclear, and nuclear sublibraries) resulting in a set of 92 peptides containing NLS‐like sequences found in 76 proteins. Using these peptides we could generate consensus motifs that matched four of the previously reported NLS classes (Fig 5A; class I‐IV). We found that seven of these sequences had been previously validated as functional NLSs (Sheren & Kassenbrock, 2013; Lopez‐Mosqueda *et al*, 2016; Cappuyns *et al*, 2018; Scholler *et al*, 2018; Zhou *et al*, 2020), including the bipartite NLS of the activity‐dependent neuroprotector homeobox protein (ADNP; peptides identified: _711‐_SLAPV**KR**T**Y**EQMEFPL_‐726_ and _719‐_YEQMEFPLL**KK**R**K**LDD_‐734_). We determined the affinities for 16 peptides and established their pocket specificity using FP competition experiments using the FITC‐labeled _320‐_ **P**AA**KR**V**K**LD_‐328_ peptide from the Myc proto‐oncogene protein (Myc) (Dang & Lee, 1988) as a probe for the major pocket, and the FITC‐labeled _1307‐_P**KR**T**Y**DMMEGRVGRAI_‐1322_ peptide from the nuclear receptor corepressor 2 (NCOR2) as a probe for the minor pocket (Fig 5D; Appendix Fig S7; Dataset EV6). We found that the 16 tested peptides bound to KPNA4 with a broad range of affinities (nM–mM) and with distinct pocket specificities (Fig 5E). Three peptides bound only to the major pocket. Eight peptides with class III motif and one KRxH‐containing peptide only bound to the minor pocket. Five peptides outcompeted both probe peptides, which may be explained by the amino acid sequence matching both class II and class III motifs (e.g., _1135‐_ **P**SP**KR**K**L**_‐1139_ in ZNF532; Fig 5), and possibly by cross specificity of the two binding pockets.

The function of 16 of the putative NLSs was evaluated by fusing them to a trimer of GFP (Fig 5C; Appendix Fig S8). Using this NLS sensor, 12 out of 16 tested peptides were confirmed as functional NLSs (Fig 5E). The two lowest affinity KPNA4 ligands (KDR_958–973_ and MKL2_572–587_) failed to function as NLSs, suggesting a correlation between affinity and NLS function. The function of three NLSs were further validated in context of their full‐length proteins (heterogeneous nuclear ribonucleoproteins C1/C2 [HNRNPC); DNA‐dependent metalloprotease SPRTN (SPRTN); Holliday junction recognition protein (HJURP)). We expressed mCherry‐tagged wild‐type and NLS mutant proteins (HNRNPC _98‐_ **KR/AA**_‐99_; SPRTN _407‐_ **KR/AA**_‐408_; HJURP _681‐_ **KR/AA**_‐682_) in HEK293 cells and found that wild‐type HJURP, HNRNPC, and SPRTN proteins were efficiently targeted to the nucleus, while mutants were retained in the cytoplasm (Fig 5F). The KPNA4 selections thus successfully identified functional NLSs.

### The amino acid resolution binding site information allows accurate predictions of effects of disease mutations and PTMs

**Figure 6:**
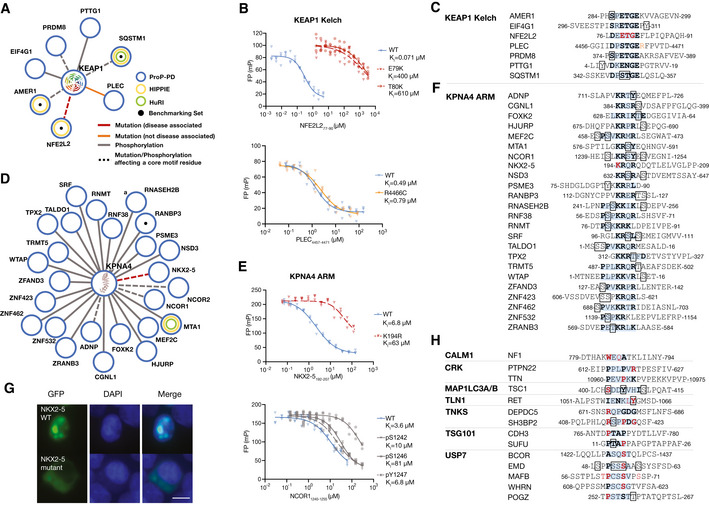
The amino acid resolution binding site information allows accurate predictions of functional effects of disease mutations and PTMs PPI networks of KEAP1 showing reproducibly selected high/medium confidence interactions with mutations or phosphosites overlapping with the binding motif or in the flanking regions (± 2 residues).The disease‐associated mutation is colored in red (orange if not disease associated). Phosphosites are colored in gray. Dashed‐edges represent mutations or phosphosites in motif residues.FP competition experiments of wild‐type and disease mutant peptides binding to KEAP1 Kelch using FITC‐NFE2L1_228–243_ as probe (*n* = 3, technical replicates, shown are individual data points. Source data are provided).Peptide sequences related to the interactions shown in panel (A).PPI networks of KPNA4 showing reproducibly selected high/medium confidence interactions with mutations or phosphosites overlapping with the binding motif or in the flanking regions (± 2 residues).FP competition experiments of wild‐type, disease mutant, and phospho‐peptides binding to KPNA4. The affinities of NXK2‐5 wild‐type and K194R mutant for KPNA4 were determined using FITC‐Myc_320–328_ as a probe; the affinities of unphosphorylated and phosphorylated NCOR2 peptides were determined using FITC‐NCOR2_1307–1322_ as probe (*n* = 3, technical replicates, shown are individual data points. Source data is provided).Peptide sequences related to the interactions shown in panel (D).Representative cellular localization experiments of the GFP‐based NLS sensor fused to wild‐type or K194R mutant NKX2‐5_192–207_ peptide. HEK293 cells were transiently transfected with the NLS sensor and fixed 36 h after transfection, and imaged using epifluorescence microscopy. The nucleus was stained with DAPI. The scale bar indicates 10 μm (*n* = 3, independent experiments).Peptides for additional baits with disease‐associated mutations in the consensus binding motif. Data information: For (C), (F) and (H): Motifs are highlighted with blue background and key residues are indicated in bold letters, phosphosites are indicated by a box, and disease‐associated mutations of SLiMs are indicated in red bold letters. Source data are available online for this figure.

A key advantage of ProP‐PD is the definition of the binding sites for the bait protein at amino acid resolution. To highlight the utility of such data we assayed the effect of mutations and phosphorylation on a representative set of newly discovered interactions. The medium/high confidence data from selections against all HD2 libraries were combined to provide an extensive network of PPIs. The PPI network was annotated with a variety of biologically relevant information using the PepTools server (Dataset EV9). These data corroborated the biological relevance of ligands based on the overlap of the contextual information of the bait and prey (shared complex, localization, and functional terms) as previously described for the GO term enrichment analysis (Dataset EV7). The PPI network annotation was then used to identify binding interfaces that overlap with disease mutations (Dataset EV10) or phosphosites (Dataset EV11). We found 183 peptides with 313 unique mutations, at 253 sites (Appendix Fig S9), including missense mutations of the TNK1‐binding peptide from the SH3‐binding protein 2 (SH3BP2 _408‐_PQLPHLQ **R** SP **PDG** QSF_‐423_; affected residues underlined) that are known to abrogate the interaction with TNKs and are associated with cherubism (Ueki *et al*, 2001; Imai *et al*, 2003; Lo *et al*, 2003). We tested the effects of four disease associated mutations on binding: the E79K and T80K mutations in the KEAP1‐binding motif of NFE2L2 (Fig 6A–C) linked to early onset of multisystem disorder (Huppke *et al*, 2017), a R4466C mutation in the flanking region of the KEAP1‐binding motif in PLEC, which is linked to epidermolysis bullosa simplex but with uncertain significance on pathogenicity (Landrum *et al*, 2014), and a K194R mutation in the KPNA4‐binding peptide from homeobox protein Nkx‐2.5 (NKX2‐5) associated with atrial septal defects (Fig 6D–F) (Schott *et al*, 1998; Benson *et al*, 1999; McElhinney *et al*, 2003). Both NFE2L2 mutants conferred a striking 1,000‐fold loss of KEAP1 affinity. In contrast, PLEC_4457–4471_ R4466C had no effect on binding, consistent with its position outside of the motif. The K194R mutation of NKX2‐5_192–207_ resulted in a 10‐fold loss of affinity of KPNA4 binding (Fig 6E), which led to a cytoplasmic localization of the GFP‐based NLS sensor when fusing it to the K194R mutant NKX2‐5_192–207_ peptide (Fig 6G). In contrast, when the wild‐type NKX2‐5_192–207_ peptide was fused to the NLS sensor it led to a nuclear localization of the GFP fluorescence, with accumulation in nucleoli. The analysis demonstrates how the amino acid resolution footprinting of protein‐binding sites in IDRs combined with the annotations from PepTools can be used to pinpoint effects of disease‐associated mutations (Fig 6). We further found 6,724 PTM sites in 2,755 high/medium confidence unique peptides, including 5,868 phosphorylation sites (Dataset EV11). We tested the effect of three reported phosphosites in the class III NLS of NCOR1 (pS1242, pS1246, and pY1247), and found that while pS1242 and pY1247 phosphorylation had minor effects on the affinity for KPNA4, the pS1246 conferred a marked loss of affinity (Fig 6E). Thus, the results can be used to identify phospho‐switches that tune binding affinities.

## Discussion

We present a powerful experimental and bioinformatics pipeline for the proteome‐scale discovery of motif‐based interactions that generate results of similar quality to approaches such as AP‐MS and Y2H. In addition, ProP‐PD provides amino acid resolution information on the binding sites. An added advantage is that there is no bias for interactions with highly expressed proteins, or cell type or cell state dependence as the selections are performed *in vitro* and bait proteins are challenged with the full IDRs of the proteome. Indeed, 471 of the found interactions are with “poorly studied proteins” as classified in the Pharos database (Nguyen *et al*, 2017). Thus, we shed light on understudied parts of the proteome. We acknowledge that the results are solely based on *in vitro* interactions between isolated domains and short peptides of target proteins, and as for other large‐scale interaction data, detailed validations at the level of full‐length proteins and in a cellular setting are required to validate biologically relevant interactions. Nevertheless, as shown for the KPNA4‐binding NLSs, the SLiMs identified by ProP‐PD do generally function within the whole protein context. This notion is supported by results generated based on the first‐generation human disorderome library, which among other things has uncovered functional docking sites for protein phosphatases (Wu *et al*, 2017; Ueki *et al*, 2019; Wigington *et al*, 2020).

The recall (19.3%) of SLiM‐based interactions for selections against the HD2 P8 library is twice the recall of the reference methods Y2H and AP‐MS, and is thus a high recall for large‐scale interaction analysis. Nevertheless, the approach failed to capture 80% of known interactions. Factors limiting the recall may for example be the competition with one million other peptides during the selection and missing potential binding interfaces outside of the core binding site. The screen‐to‐screen (each performed in triplicate) reproducibility was found to be around 30% between different libraries, which might point in the direction that the recall can be improved by performing additional replicates. This may be particularly relevant for bait proteins that like KPNA4 and the WW domains have thousands of potential ligands in the proteome.

An alternative way to improve the recall and precision could be to reduce the search space and thereby limit competition. We tested the use of smaller compartment‐specific sublibraries, which did not make much of a difference for most cases, with notable exception for the endomembrane library. The observed improvement is related to PEX14, which binds to peptides containing a sequence pattern of acidic and hydrophobic residues (Fig 4B) much like the nine amino acid transactivation domains that interact with transcriptional regulators (Piskacek *et al*, 2007). PEX14 is likely exposed to a large cohort of hydrophobic/acidic motifs during the HD2 P8 selection that the protein would normally not encounter in the cell and that outcompetes the biologically relevant binders. Compartment‐specific sublibraries may thus be beneficial for binding pockets and motif combinations for which the inherent specificity of the peptide is relatively low and spatiotemporal constraints significantly contribute to specific binding. Another factor that is critical for successful outcomes of ProP‐PD selections is the quality of the purified proteins, including how well the proteins tolerate the immobilization method used during screening. Alternative immobilization methods could be tried for proteins that fail to enrich for binding peptides like the domains of MAD2L1, SPSB1, SUFU, and WDR5.

One of the fixed parameters of this study was the 16 amino acid residue length of the displayed peptides, which should be sufficient to cover most of the SLiM instances. However, we note that the peptides are too short to capture certain motif classes, for example bipartite NLSs. For some cases, the peptides may be too short to capture the contribution of motif‐flanking regions. These regions may contribute to increase both affinity and specificity, as recently shown for the EVH1 domain of ENAH (preprint: Hwang *et al*, 2021). A future direction may thus be to create ProP‐PD libraries expressing longer peptides, as previously done using the T7 phage (Larman *et al*, 2011).

Among the challenges not addressed in this study are those related to the identification of SLiM‐based interactions that rely on PTMs, such as phosphorylation. These challenges might be addressed by using PTM‐mimetic mutations (Sundell *et al*, 2018), by treating the phage library with enzymes, or by using an expanded genetic code (Tian *et al*, 2004; Oller‐Salvia & Chin, 2019). Nevertheless, the annotations provided by PepTools suggest potential regulation by PTMs. PepTools also provides information on disease‐associated mutations in identified peptides, which can give clues about the underlying molecular determinants of diseases. Similarly, we uncovered novel interactions for known drug targets thereby improving our understanding of the therapeutically targeted proteome. Indeed, 50 of the discovered motif‐containing proteins have at least one approved drug (Dataset EV9). For two of the screened baits, MDM2 and KEAP1, small molecules have been developed to therapeutically target the motif‐binding pocket (Burgess *et al*, 2016; Colarusso *et al*, 2020) and the newly discovered ligands of these proteins may help explain off‐target effects of these inhibitors.

In conclusion, we present a resource of more than 2,000 human PPIs with amino acid resolution of binding sites. We foresee that ProP‐PD will contribute to mapping of the human interactome over the next decade and provide detailed information on binding motifs and a deeper understanding of genotype‐to‐phenotype relationships. Given that there are more than 200 known families of SLiM‐binding domains and in the range of 100,000 motif‐based interactions to uncover (Tompa *et al*, 2014), there is a sizable task ahead for the scientific community. Through the proteome‐scale amino acid resolution footprinting offered by ProP‐PD we hope to contribute insights into a considerable part of these interactions over the years to come.

## Materials and Methods

### Reagents and Tools table

**Table:** 

Reagent/resource	Reference or source	Identifier or catalog number
HEK293	Sigma	Cat. 85120602
*Escherichia coli* SS320	Lucigen	Cat. 60512‐1
M13KO7 helper‐phage	ThermoFisher	Cat. 18311019
*Escherichia coli* BL21(DE3) gold	Agilent Technology	Cat. 230132
*Escherichia coli* OmiMAX	ThermoFisher	Cat. C854003
**Recombinant DNA**		
pETM33	EMBL	
pETM41	EMBL	
For detailed information on expression constructs see Dataset EV2		
mCherry2‐C1	Addgene	#54563
pEGFP‐C1 vector	Clontech (Leuwen)	
Phagemid p8	Sidhu lab (Chen *et al*, 2015)	
Phagemid p3	Ernst lab (Putyrski *et al*, 2020)	
**Antibodies**		
M13 HRP‐conjugated M13 bacteriophage antibody	Sino Biological Inc	Cat: 11973‐MM05T‐H
**Oligonucleotides and sequence‐based reagents**		
Oligonucleotides	CustomArray	
**Chemicals enzymes and other reagents**		
Phusion polymerase High‐Fidelity polymerase	Thermo Scientific	Cat: F631XL
*ExoI*	Thermo Scientific	Cat: EN0581
Nucleotide removal kit	Qiagen	Cat. No. / ID: 28306
T4 polynucleotide kinase	Thermo Scientific	Cat: EK0031
T7 DNA polymerase	Thermo Scientific	Cat: EP0081
T4 DNA ligase	Thermo Scientific	Cat: EL0014
50‐bp marker	Thermo Scientific	Cat: 10416014
Mag‐bind Total Pure NGS	Omega Bio‐tek	Cat: M1378‐01
QIAquick Gel extraction Kit	Qiagen	Cat: 28706X4
Quant‐iT PicoGreen dsDNA Assay Kit	Molecular probes by Life technologies	Cat: P7589
TMB substrate	Seracare KPL	Cat: 5120‐0047
Gibson Assembly^®^	New England Biolabs	Cat: E5510S
DMEM GlutaMAX™ Supplement	Gibco™	Cat: 61965026
FBS	Gibco™	Cat: A338200
Nonessential Amino Acids Solution	NEAA, Gibco™	Cat: 11140035
FuGENE^®^ HD	Promega	Cat: E2311
Image‐iT™ Fixative Solution	Thermo Fisher	Cat: FB002
ProLong™ Glass Antifade Mountant with NucBlue™ Stain	Invitrogen™	Cat: P36981
cOmplete™ EDTA‐free Protease Inhibitor Cocktail	Roche	Cat: 4693132001
GSH Sepharose 4 Fast Flow Media	Cytiva	Cat: 17513201
Ni Sepharose 6 Fast Flow	Cytiva	Cat: 17531801
**Plates**		
96‐well Flat‐bottom Immunosorp MaxiSorp plates	Nunc, Roskilde, Denmark	#439454
384‐well Flat‐bottom Immunosorp MaxiSorp plates	Nunc, Roskilde, Denmark	#464718
96‐well half area black Flat‐bottom Nonbinding surface plates	Corning, USA	#3993
**Software**		
GraphPad Prism version 9.0.0 for MacOS	GraphPad Software, San Diego, California USA, www.graphpad.com	
Zen software (V3.2, blue edition)	Zeiss, Germany www.zeiss.com	
ImageJ	NIH https://imagej.nih.gov/ij/	
Cytoscape	San Diego, California USA https://cytoscape.org	
PyMOL Version 2.1.1	New York, New York, USA Schrodinger LLC	
Python version 3	Van Rossum and Drake (2009)	
Matplotlib	Hunter (2007)	
Seaborn	Waskom *et al* (2017)	
R version 4	R Core Team (2015)	
ggplot2	Wickham (2016)	
**Other**		
iD5	Molecular Devices	
PCR machine	Biometra TGradient	
Illumina MiSeq v3 run, 1 × 150 bp read setup, 20% PhiX	NGS‐NGI SciLifeLab facility	
Nanodrop ND‐1000	Thermo Fisher	
Zeiss imager Z2 microscope using C11440 camera (Hamamatsu) and 40x oil objective lens (N.A. 1.4)	Zeiss	

### Methods and Protocols

#### Computational ProP‐PD library design

##### Defining the ProP‐PD search space

We defined the ProP‐PD search space as the intrinsically disordered regions (IDRs), including loops in structured regions, of the human proteome accessible to intracellular proteins. A dataset of the 20,206 reviewed human proteins was retrieved from UniProt (release 2018_02) (UniProt Consortium, 2019). Intracellular protein regions were defined by removing: (i) proteins with the keywords “Secreted,” unless they also had the keywords “Cytoplasm” or “Nucleus”; and (ii) transmembrane regions and the extracellular regions of transmembrane proteins based on UniProt annotation.

##### Defining the disordered regions of the human proteome

Intrinsically disordered regions and large loops in structured regions of the human proteome were defined using three sources of data: (i) disorder state predictions, (ii) surface accessibility from solved structures of the protein; and (iii) surface accessibility homology mapped from solved structures.

We used IUPred (Dosztanyi *et al*, 2005) to calculate per residue disorder propensity scores. Scores were calculated on the full‐length sequence of proteins from the UniProt. In the cases where UniProt annotated chain and topology domains were available, the chain and topology domains of the protein were analyzed independently and these data were used. An IUPred disorder propensity score cut‐off of 0.4 was applied to each residue of each protein resulting in binary accessible (disordered) or inaccessible residue classifications.

When a solved structure(s) of a protein was available, surface accessibility (SA) scores were calculated for the structure(s). The SA score for a residue was calculated as the proportion of the amino acid that is accessible to water molecules in the solved structure normalized by the maximum possible accessibility for that amino acid in a peptide chain (as defined for five‐residue peptides with a central query amino acid flanked by two glycine residues [GGXGG]). The SA score for a residue that is unresolved in the structure was set to 100% accessibility. For protein structures containing a multiprotein complex, regions that are < 25 amino acids in length are discarded as they are unlikely to fold in the absence of a binding partner and chains with < 10 intramolecular contacts per residue on average are not retained as this is a hallmark of bound IDRs. When multiple structures are mapped to the same residue the median SA score for the residue is used. A SA cut‐off of 33% is applied resulting in binary accessible or inaccessible residue classifications.

Homology mapped structures were defined by searching the query protein against a database of PDB structure constructs using the BLAST tool and retaining hits with an e‐value cut‐off of 10^−15^ and coverage cut‐off of 85% of the structure are retained as homology mapped structures. The query protein and PDB structure constructs were aligned using local pairwise alignment with a BLOSUM62 matrix. SA scores for the homology‐mapped structures were calculated for the PDB structure as described above for the direct structural information and the SA scores were mapped by pairwise alignment to the query protein. As above, a SA cut‐off of 33% was applied resulting in binary accessible or inaccessible residue classifications.

The accessible/inaccessible categories to define IDRs and loops information were used in a hierarchy: direct or homology‐mapped experimental accessibility data were used when available, otherwise disorder predictions were used (i.e., when experimental information is available it was used in place of predictions). The resulting binary accessible/inaccessible categories were smoothed to remove short regions of length 4 or less that are not consistent with the flanking region category. Regions of order in a disordered region that are < 25 amino acids in length were defined as accessible and retained. Any 16‐mer peptide window where at least 8 of the 16 amino acids were defined as accessible based on the rules above was retained as the ProP‐PD search space.

##### Defining the peptides

The ProP‐PD search space was tiled with peptides of length 16 amino acids overlapping by 12 amino acids. Cytoplasmic loops of length 8 or greater that were predicted as disordered were retained. All cysteines were replaced with alanine to avoid issues with unpaired cysteines in the phage coat proteins.

##### Design of the oligonucleotides

The designed peptide sequences were reverse translated into oligonucleotides by stochastically choosing codons to match the codon usage of *Escherichia coli*. For special cases, that is, when no overlapping peptide exists or the peptide was at a terminus, we created two distinct oligonucleotides for the peptides. The primers required for annealing in the construction of the phagemid library were added: (5′ CAGCCTCTTCATCTGGC and 3′ GGTGGAGGATCCGGAG). Finally, we redesigned oligonucleotides to remove SmaI restriction sites (GGGCCC or CCCGGG) or self‐complementarity of greater than seven contiguous nucleotides.

##### Defining HD2 sublibraries

Peptides from the human proteins were split into five protein pools using GO term and UniProt Keyword annotation:

1. Endomembrane: Mapping to GO terms “endomembrane system” or its descendants, or the UniProt Keywords “Endoplasmic reticulum membrane,” “Endoplasmic reticulum,” “Golgi apparatus membrane,” “Golgi apparatus”, “Golgi cisterna membrane,” “Golgi membrane,” “ER to Golgi transport vesicle membrane,” “Cytoplasmic vesicle membrane,” “Cytoplasmic vesicle,” “Early endosome membrane,” “Early endosome,” “Endosome membrane,” “Late endosome membrane,” “Late endosome,” or “Recycling endosome membrane”.
2. Nuclear: Mapping to GO term “nucleus” or “chromosome,” or their descendants, or the UniProt Keywords “Nucleus” or “Chromosome”.
3. Cytoplasmic: Mapping to GO terms cytoplasm, mitochondrion, cytoskeleton, cilium or plasma membrane or their descendants, or the UniProt Keywords “Cytoplasm,” “Cell membrane,” “Membrane”.
4. Extracellular: No *Cytoplasmic, Nuclear* or *Endomembrane* sublibrary assignment. No transmembrane regions in the protein. Mapping to GO terms “extracellular region” or “extracellular region part” or their descendants, or the UniProt Keywords “extracellular space,” “extracellular exosome,” ”extracellular region,” “extracellular exosome,” ”exocyst,” “extracellular space,” ”endoplasmic reticulum lumen,” or “Endoplasmic reticulum lumen”.
5. Other sub‐library: Proteins without localization and therefore no sublibrary assignment.

Designed oligonucleotides were split into pools of 92,918 to match the size of the pools generated by the commercial provider. From these pools, we created six sublibraries of different sizes:

1. Cytoplasm (Cytoplasm and no Nucleus or Endomembrane System—3 × 92.9 k pools).
2. Nucleus (Nuclear and no Cytoplasm or Endomembrane System—3 × 92.9 k pools).
3. Endomembrane System (Endomembrane—2 × 92.9 k pools).
4. Nucleocytoplasmic shuttling (Nucleus and Cytoplasm or Endomembrane System—4 × 92.9 k pools).
5. Extracellular (Extracellular 1 × 92.9 k pools).
6. Other (1 × 92.9 k pools).

Unused space on the chips for each sublibrary was filled with redundant peptides encoded by distinct synonymous oligonucleotides. An interactive website to explore the full library design is available at http://slim.icr.ac.uk/phage_libraries/human/.

#### Construction of the phage display libraries

We obtained oligonucleotides (CustomArray) encoding the designed peptides flanked by phagemid annealing sites. The oligonucleotides were used to create libraries of genes coding for the designed peptides fused to the N‐terminal part of the P8 or P3 protein flanked by glycine‐serine linker regions following a published protocol (Ali *et al*, 2020).

1. Oligonucleotides from each sublibrary pool were combined and used as template for 15 cycles of PCR amplification (denaturation at 98°C for 10 s, annealing at 56°C for 15 s and amplification at 72°C for 10 s) using Phusion polymerase (Thermo Scientific) and primers complementary to the constant annealing regions flanking the designed library sequences.
2. Remaining oligonucleotides and nucleotides were removed by *ExoI* (Thermo Scientific) treatment (HD2 P8) or using a nucleotide removal kit (Qiagen; HD2 P3).
3. The cleaned PCR products were phosphorylated using T4 polynucleotide kinase (Thermo Scientific) for 1 h at 37°C and annealed to phagemid ssDNA (90°C for 3 min, 50°C for 3 min and 20°C for 5 min).
4. dsDNA was synthesized using T7 DNA polymerase (Thermo Scientific) and T4 DNA ligase (Thermo Scientific) at 20°C for 16 h.
5. The dsDNA generated from each sublibrary pool was purified from a 1% agarose gel, eluted using ultrapure H_2_0, and electroporated to *E. coli* SS320 (Lucigen) electrocompetent cells preinfected with M13KO7 helper‐phage (ThermoFisher) prepared as described elsewhere (Rajan & Sidhu, 2012).
6. The phage was allowed to propagate for 24 h in 2xYT (10 g yeast extract, 16 g tryptone, 5 g NaCl per L) medium.
7. The phage was precipitated from the supernatant by the addition of 1/5^th^ volume of 20% PEG8000/2.5 M NaCl followed by centrifugation at 27,000 *g* for 20 min.
8. The phage pellets were dissolved in phosphate‐buffered saline (PBS, 137 mM NaCl, 2.7 mM KCl, 95 mM Na_2_HPO_4_, 15 mM KH_2_PO_4_ pH 7.5).
9. The sublibrary phage titers were determined before pooling them into the final HD2 library. The resulting HD2 P8 library was reamplified and stored at −80°C.

#### Next‐generation sequencing of the phage datasets

1. Peptide‐coding regions of the naive phage libraries or binding enriched phage pools (5 µl for 50 µl PCR reaction) were amplified and barcoded using a dual barcoding strategy (McLaughlin & Sidhu, 2013) using Phusion High‐Fidelity polymerase (Thermo Scientific) for 22 cycles.
2. The PCR products were confirmed through 2% agarose gel electrophoresis stained with GelRed, using a 50‐bp marker (Thermo Scientific).
3. The amount of the PCR products (25 µl) was normalized using 25 µl Mag‐bind Total Pure NGS (Omega Bio‐tek).
4. The normalized amplicons were pooled (10 μl from each reaction).
5. The resulting amplicon pool was further purified from a 2% agarose gel (QIAquick Gel extraction Kit Qiagen) with GelRed staining and eluted in TE (10 mM Tris‐HCl, 1 mM EDTA. pH 7.5) buffer following the provided protocol, except dissolving the agarose gel by 30 min incubation at room temperature instead of vortexing.
6. The dsDNA concentration of the purified amplicon pool was determined with Quant‐iT PicoGreen dsDNA Assay Kit (Molecular probes by Life technologies) and analyzed by Illumina MiSeq v3 run, 1 × 150 bp read setup, 20% PhiX by the NGS‐NGI SciLifeLab facility, which returned an average of 33,000 reads per set of barcodes (on average 18 million reads per NGS run).

#### Demultiplexing and processing of NGS data

NGS results processing was performed as described in detail in published protocol (Ali *et al*, 2020). In brief:

1. The results of ~ 500 pooled experiments, each one tagged with a unique combination of 5′ and 3′ barcodes, were demultiplexed and cleaned using in house custom Python scripts.
2. Reads with an average quality score of 20 or more were retained, and their adapter regions and barcodes were determined allowing a maximum of one mismatch per adapter and/or barcode. Reads with ambiguous barcodes presenting a mutation that by allowing one mismatch can match them to more than one of the designed barcodes were excluded.
3. The subset of high‐quality, unambiguously identified reads was trimmed by removing their adapter and barcode regions.
4. The coding sequences were grouped into separated FASTA files where one file was produced for each barcode set.
5. Finally, tables were built from each demultiplexed FASTA file where each unique trimmed oligonucleotide was translated to an amino acid sequence and NGS sequencing count.

#### Library coverage and quality check

To assess the quality of synthesized and cloned phage libraries we analyzed the coverage percentage of the physical phage library compared to the designed phage library. Multiple aliquots for each naive phage library were sequenced through NGS and the total number of combined unique sequences matching the library design was used to calculate the “sequenced coverage”. For the estimation of the “maximum coverage”, each sequenced aliquot was picked in a random order and their cumulative contribution of new unique sequences versus the total number of contributed reads was assessed. The process of randomizing the picking order of sequenced aliquots was repeated 10 times and the maximum coverage was estimated by extrapolation to an infinite number of reads by nonlinear regression to the following equation:

$$Y=\frac{Y_{\max1}\;*\;X}{a+X}+\frac{Y_{\max2}\;*\;X}{b+X}.$$

The “predicted maximum coverage” is the sum of *Y*_max1_ and *Y*_max2_, as determined by KaleidaGraph version 4.5.4 for Mac OS (Synergy Software) and the reported error is the propagated fitting error of this sum. As the HD2 P8 library was constructed from the HD2 sublibraries, the contribution of each sublibrary was also taken into account when calculating the sequenced and maximum coverage of the HD2 P8 library. The library sequencing confirmed 91.5% of the sequences were present in the library and the extrapolated maximum library coverage percentage was 96 ± 5% (Appendix Fig S1, Dataset EV1). 73.6% of the incorporated oligonucleotide sequences were of correct length and 89.4% of them encoded for peptides that matched the library design. There was no major overrepresentation of sequences in the constructed library.

#### Expression & purification of bait proteins for phage selections

1. *Escherichia coli* BL21(DE3) gold bacteria (Agilent Technology) containing plasmids encoding 6‐His‐GST fusion proteins (Dataset EV2) were grown in 100 ml 2xYT at 37°C and 200 rpm.
2. For each protein, expression was induced with 1 mM isopropyl β‐d‐1‐thiogalactopyranoside (IPTG) and was allowed to proceed for 4 h at 30°C.
3. Bacteria was harvested for 10 min at 4,500 *g*.
4. The bacterial pellet was resuspended in lysis buffer (7.5 ml PBS supplemented with 1% Triton, 10 µg/ml DNaseI, 5 mM MgCl_2_, lysosome, and cOmplete™ EDTA‐free Protease Inhibitor Cocktail [Roche]) and was incubated for 1 h on ice.
5. The suspension was sonicated to destroy remaining DNA and support the lysis, and the cell debris was pelleted by centrifugation (1 h, 16,000 *g*).
6. Proteins were batch purified from the supernatant using GSH Sepharose 4 Fast Flow Media (Cytiva) following the manufacturer's protocol. The protein concentration was estimated using a Nanodrop ND‐1000 spectrometer and the purity was confirmed through SDS–PAGE.

#### ProP‐PD selections

Phage selections were performed following the previously described protocol (Huang & Sidhu, 2011; Ali *et al*, 2020).

1. In brief, 10 µg GST‐tagged bait protein or GST (total volume 100 µl in PBS) was immobilized in 96‐well Flat‐bottom Immunosorp MaxiSorp plates (Nunc, Roskilde, Denmark) for 18 h at 4°C.
2. Wells were blocked with 200 µl 0.5% (w/v) BSA in PBS for 1 h, at 4°C. GST‐coated wells were washed four times with 200 µl PT (PBS + 0.05% (v/v) Tween20).
3. The phage library (10^11^ phage in 100 µl PBS) was transferred to each of the GST‐coated wells for removal of phage that bound nonspecifically.
4. After 1 h the library was transferred to protein coated wells. After 2 h of incubation unbound phage were removed by washing the wells with 5 × 200 µl PT.
5. Bound phages were eluted with 100 µl log phase *E. coli* OmiMAX for 30 min at 37°C.
6. Bacteria were hyperinfected by the addition of 10^9^ of M13KO7 helper phage per well for 45 min at 37°C.
7. The bacteria (100 µl) were transferred in 1 ml 2xYT supplemented with 100 µg carbenicillin, 30 µg kanamycin, 0.3 mM IPTG, and were incubated at 37°C for 18 h upon shaking.
8. Phages were harvested by centrifugation at 2,000 *g* for 10 min.
9. Phage supernatants were transferred into a fresh 96‐well plate, pH adjusted by the addition of 1/10 volume 10× PBS, and heat inactivated for 10 min at 65°C.
10. The resulting amplified phage pools were used as in‐phage for the next day of selection. To enrich for binding phage, this procedure was repeated in total in four rounds of selections.

To evaluate the enrichment of binding phage, pooled phage ELISA was performed in 384‐well Flat‐bottom Immunosorp MaxiSorp plates (Nunc, Roskilde, Denmark).

1. For each round of selections bait protein and GST control were coated (5 µg in 50 µl /well) for 18 h at 4°C.
2. The wells were blocked with 100 µl 0.5% BSA in PBS, for 1 h, at 4°C.
3. Phage (50 µl) from the different rounds of selections were allowed to bind to the bait protein or GST‐coated wells.
4. After 1 h unbound phage was washed away with 4× 100 µl PT.
5. Bound phage was detected using 50 µl M13 HRP‐conjugated M13 bacteriophage antibody (Sino Biological Inc; 1:5,000 diluted in PT, 0.05% BSA) for 1 h, at 4°C.
6. The wells were washed with 4× 100 µl PT and once with 100 µl 1× PBS. TMB substrate (40 µl, Seracare KPL) was added to detect the bound antibody.
7. The enzymatic reaction was stopped by the addition of 40 µl 0.6 M H_2_SO_4_. The absorbance at 450 nm was determined with an iD5 (Molecular Devices).

#### ProP‐PD peptide analysis

##### Peptide processing

Peptide data were created for each ProP‐PD selection as described in “Demultiplexing and processing of NGS data,” where the DNA sequences were translated to amino acid sequences, resulting in peptides associated with sequencing read counts. After that:

1. Peptides observed only a single time in the NGS sequencing data were discarded.
2. The NGS sequencing counts for each selection day were normalized by dividing the peptide sequencing counts by the sum of the sequencing counts for all peptides in the selection to create the *normalized peptide sequencing count*. Normalization was performed to allow the comparison between different screens and sequencing batches.
3. Peptides for each selection day for a replicate were pooled and the mean *normalized peptide sequencing count* for each replicate was calculated per peptide.
4. A merged list of peptides found through replicate screens against a given bait protein was created. For each peptide, the number of replicate screens it occurred in and the identification of other overlapping peptide sequences were annotated.

##### PepTools peptide mapping and annotation web server

We developed PepTools, a novel peptide annotation tool, to analyze peptide data from experimental motif discovery methods such as ProP‐PD. PepTool expands on the SLiMSearch and PSSMSearch motif discovery framework (Krystkowiak & Davey, 2017; Krystkowiak *et al*, 2018) to map peptides to a query proteome and annotate them with structural, evolutionary, functional, genomic, and proteomic data. The PepTools framework is available as a web server that is freely accessible at http://slim.icr.ac.uk/tools/peptools/. PepTools has a range of functions and features to quickly pinpoint putative biologically relevant motif instances in a list of peptides:

1. Previously validated instances: overlap with previously validated motif instances retrieved from the ELM database.
2. Previously validated interactions with bait protein/domain: evidence of an interaction of the peptide‐containing protein with a bait protein or a protein containing a bait domain.
3. Shared annotation with bait protein/domain: shared functional annotations or localization of the peptide‐containing protein with a bait protein or a protein containing a bait domain (calculated as described previously (Krystkowiak *et al*, 2018)).
4. Accessibility information: accessibility information from annotations (topology, domains), predictions (intrinsic disorder, transmembrane regions), and experimental sources (structure‐derived surface accessibility).
5. Consensus/PSSM annotation and filtering: peptides can be scanned with motif consensuses to highlight peptides with motif matches and peptides can be scored and ranked with a user‐defined PSSM. Peptides can also be filtered using the motif consensus or peptide PSSM scores.
6. Key specificity determinant residue annotation: residue‐specific annotation such as SNPs, PTMs and mutagenesis are highlighted if they overlap “key” residues based on a user‐defined motif consensus.
7. Enriched motif specificity determinants: Peptides can be analyzed for enriched motifs using the SLiMFinder motif discovery tool (Davey *et al*, 2010). The motif specificity determinants of the enriched motifs can be visualized with a heatmap or sequence logo.
8. User annotation: External peptide related data can be added to the input resulting in sortable extra columns on the results page.
9. Extensive peptide filtering functionality: ontology, interaction, and localization information allow the use of *a priori* knowledge of a motif's biological context for peptide prioritization.
10. Masked amino acid: specific amino acids can be masked to allow peptides with experimentally required substitutions to be mapped correctly (e.g., cysteine to alanine substitutions in a ProP‐PD library).
11. A detailed description of PepTools functionality is available on the PepTools help page at http://slim.icr.ac.uk/tools/peptools/help
12. The filtering, enrichment, and annotation functionality is as described for the SLiMSearch and PSSMSearch (Krystkowiak & Davey, 2017; Krystkowiak *et al*, 2018).

Key sources of data used by PepTools for the ProP‐PD analysis include (i) protein data (UniProt (UniProt Consortium, 2019)); (ii) localization data (UniProt and GO (The Gene Ontology, 2019)); (iii) accessibility data (structure data retrieved from PDB processed using DSSP and IUPred calculated disorder scores (Dosztanyi *et al*, 2005; Touw *et al*, 2015)); (iv) domain data (retrieved from Pfam (Finn *et al*, 2014)); (v) interaction data (retrieved from IntAct (Orchard *et al*, 2014), HIPPIE (Alanis‐Lobato *et al*, 2017), BioPlex (Huttlin *et al*, 2021), and HuRI (Luck *et al*, 2020)); (vi) post‐translational modifications (retrieved from the UniProt, phospho.ELM (Dinkel *et al*, 2011) and PhosphoSitePlus (Hornbeck *et al*, 2012) database and a large phosphoproteomics dataset from Ochoa *et al* (2020)); and (vii) disease‐relevant SNPs (data from gnomAD, dbSNP, COSMIC curated, TOPMed, NCI‐TCGA COSMIC, NCI‐TCGA, ExAC, Ensembl, ESP, ClinVar, 1000 Genomes retrieved from the EBI API (Nightingale *et al*, 2017), and UniProt Human polymorphisms and disease mutations (UniProt, 2019)).

##### PepTools peptide mapping and annotation

Selected peptides matching the designed peptide length (16 amino acids) were mapped to the human proteome using PepTools. Alanine residues were permitted to be variable in the PepTools peptide mapping to allow the remapping of cysteine residues converted to alanine in the ProP‐PD library design. Peptides were annotated with structural, evolutionary, functional, genomic, and proteomic data. For each bait, the peptides for each replicate were compared to define replicated peptides and overlapping peptides. Overlapping peptides were mapped using their protein mapping (rather than sequence) and the peptides defined as overlapping can be present in different replicates for the same bait. For the benchmarking analyses, to remove biases caused by peptides that mapped to two or more closely related paralogues, peptides were mapped to a single primary paralogue as defined by the UniProt Reference Clusters (UniRef) clusters.

##### Motif enrichment, specificity determinant score, and motif‐containing peptides

The replicated peptides and overlapping regions of overlapping peptides for each bait were analyzed for enriched motifs using the SLiMFinder motif discovery tool (Krystkowiak & Davey, 2017). SLiMFinder was run using *minic* (Minimum information content for returned motifs) of 1.1, *equiv* (list of groupings of physicochemically similar residues to use for ambiguous positions) of AGS, ILMV, IVMLFAP, IVMLFWHYA, FYWH, KRH, ST, STNQ, DEST, and *maxwild* (the maximum number of consecutive wildcard positions to allow) of 5. The amino acid frequencies of the input peptides were used as background amino acid frequencies. The top‐ranked SLiMFinder motif from the motif enrichment step was used to align the motif‐containing peptides and a PSI‐BLAST PSSM was built from the aligned peptides using the PSSMSearch motif discovery tool (Krystkowiak *et al*, 2018). The PSSM was used to calculate a specificity determinant score, a metric to define the similarity of a peptide to the enriched specificity determinants, for the selected peptides for a bait. The PSSM specificity determinant score was calculated using the probabilistic scoring method of the PSSMSearch motif discovery tool on a background model obtained from scanning the human proteome with the reversed variant of the PSSM.

##### Specificity determinant visualization

Specificity determinants were visualized as sequence logos created using the PSSM visualization software of the PSSMSearch tool. The logos display relative binomial amino acid frequencies calculated as log^−10^ of the binomial probability (*prob^aa^* *= binomial (k, n, p)* where *k* is the observed residue count at each position for a residue, *n* is the number of the instances of motifs, and *p* is the background frequency of the residue in the disordered regions of the human proteome). In the case of the multiple motifs generated for the KPNA4‐binding peptides, peptides were first separated into different cohorts following the previously established classification system (Kosugi *et al*, 2009).

#### Selection amino acid bias analysis

Amino acid frequencies were calculated for the human proteome (UniProt reviewed human proteins [release 2018_02]); the predicted disordered regions of the human proteome (IUPred score > 0.4 for the UniProt reviewed human proteins); the HD2 library design; the binding enriched phage pools from selections against HD2 P8 library; and the binding enriched phage pools from selections against combinatorial P8 library. The relative amino acid frequencies were calculated for the HD2 P8 and the combinatorial peptide phage display against the amino acid frequencies of predicted disordered regions of the human proteome and z‐scores were calculated to quantify the deviation of the amino acid frequencies.

#### Screen quality checks

##### Selection replicates benchmarking

All ProP‐PD selections were compared in a pairwise manner and the proportion of selected peptides (i) shared between the selections, or (ii) overlapping between the selections were calculated. Each ProP‐PD screen pairwise comparison was classified as replicate selections for the same bait, the same control bait and different bait proteins. The proportion of replicated and overlapping peptides in replicate selections was then compared to control and nonreplicate selections.

##### Enriched consensus benchmarking

Three consensus‐based metrics were calculated for each screened bait: (i) the enrichment of the expected ELM consensus in the peptides selected for the bait, (ii) the enrichment of a *de novo* consensus defined by SLiMFinder in the peptides selected for the bait, and (iii) the similarity of the *de novo* SLiMFinder consensus to the expected ELM consensus.

The ELM defined class(es) were curated for each bait. The enrichment of each ELM class consensus(es) was calculated in the set of peptides returned from each bait using the binomial probability (*prob^aa^* *= binomial(k,n,p)* where *k* is the number of selected peptides for the bait that match the consensus(es), *n* is the number of the peptides for the bait, and *p* is the frequency of peptides matching the consensus(es) in the whole HD2 library). The consensus enrichment for the correct consensus‐bait pairs was then compared to all other consensus‐bait pairs.

For each bait, the replicated peptides and overlapping regions of overlapping peptides were analyzed for enriched motifs using the SLiMFinder motif discovery tool (Davey *et al*, 2010) as defined in “Motif enrichment, specificity determinant score and motif‐containing peptides”. The most significant returned consensus above a *P*‐value cut‐off of 0.001 was defined as *de novo* SLiMFinder‐defined enriched motifs for the bait. The *de novo* SLiMFinder‐defined enriched motif was then compared with the correct ELM‐defined consensus(es) for the bait and against the consensus for all other ELM classes using the CompariMotif software (Edwards *et al*, 2008).

##### Enriched interactor benchmarking

The enrichment of previously validated interactors in the proteins containing peptides selected in a given bait screen was calculated based on the randomized sampling. A set of peptides corresponding to the number of peptides returned for the bait selection under investigation was randomly chosen from the pool of peptides returned across all bait selection in the study. The random selection process was repeated 10,000 times to calculate a distribution of expect values for the number of real interactors of a bait protein being returned by chance. This distribution was then compared to the observed number of validated interactors for the bait and a corresponding *P*‐value was assigned.

#### ProP‐PD motif benchmarking

##### ProP‐PD motif benchmarking datasets

We defined the *ProP‐PD motif benchmarking dataset* to test the ability of the ProP‐PD method to discover motifs. The dataset was created from 466 motif instances that were previously validated as binding to the 40 bait proteins tested in the study (Dataset EV5). Of these, 337 were covered by one or more peptides in the HD2 library and bound to one of the 35 noncontrol baits. The motifs were compiled from the ELM database (Kumar *et al*, 2020) and structurally solved peptide‐bait complexes from the PDB (Berman *et al*, 2000). For the ELM instances, each bait was annotated to an ELM class or classes and all motif instances for that class were defined as validated binders for the bait. The PDB instances were collected by retrieving structures of protein complexes that contain the bait from PDB and computationally parsing peptides bound to the domain used in the ProP‐PD screens. A further manually curated WW domain‐binding motif dataset was also collected. A list of 124 PPxY motif instances experimentally validated to bind to WW domains was collected from the literature and used to create a benchmarking dataset to evaluate ProP‐PD‐selected peptides for the NEDD4 and YAP1 WW domain screens (Dataset EV8).

##### Validated motif benchmarking

The ProP‐PD selection data was benchmarked on the *ProP‐PD motif benchmarking datasets*. For each bait, the ProP‐PD selected peptides that overlap with the validated motif instances for that bait in the *ProP‐PD motif benchmarking datasets* were compared to all other selected peptides. Four peptide metrics were compared: (i) replicated peptides (the number of replicates that the peptides are observed in), (ii) overlapping peptides (the number of distinct peptides overlapping the peptide across all replicates), (iii) specificity determinant match (the SLiMFinder‐derived PSSM match *P*‐value), and (iv) normalized peptide count (the mean normalized peptide count for the peptide across the NGS counts of the replicates). The predictive power was calculated for each metric, defined by the area under the ROC curve (AUC) and Mann–Whitney–Wilcoxon two‐sided test with Bonferroni correction *P*‐value.

#### Peptide consensus confidence assignment

Optimal cut‐offs for each of the peptide metrics were calculated using Youden's *J* statistic to maximize the true positive rate and minimize the false positive rate: (i) replicated peptide (the peptide is observed in two or more replicates); (ii) overlapped peptide (the peptide has one or more overlapping peptides in any replicate); (iii) specificity determinant match (the peptide has a SLiMFinder‐derived PSSM match with a *P*‐value < 0.0001); and (iv) high normalized peptide count (the peptide has a mean normalized peptide count > 0.0005). The four binary confidence criteria were combined for each peptide to create a single metric “Confidence level” which has four categories (“High,” “Medium,” “Low,” and “Filtered”). The “Confidence level” of “High” is assigned to instances matching all four confidence criteria, “Medium” is assigned to instances matching two or three of the criteria and “Low” is assigned to instances matching one metric criterion. Peptides that do not match any of the metric criteria are defined as “Filtered” and discarded from the results.

#### ProP‐PD interaction benchmarking

We defined the ProP‐PD interaction benchmarking dataset from the 302 interactions annotated for the 337 motif instances in the ProP‐PD motif benchmarking dataset (Dataset EV5). The 302 motif‐mediated interactions were cross‐referenced against high confidence ProP‐PD interactions, the high/medium confidence ProP‐PD interactions, the AP‐MS‐derived BioPlex 3.0 interaction dataset (Huttlin *et al*, 2021), and the Y2H‐derived HuRI interaction dataset (Luck *et al*, 2020). Next, the overlap of the interactions from the high/medium confidence ProP‐PD interactions, the BioPlex 3.0 interaction dataset, and the Y2H‐derived HuRI interaction dataset for interaction in the ProP‐PD interaction benchmarking dataset was calculated. Finally, the overlap of the high/medium confidence ProP‐PD interactions with the integrated human PPI dataset from HIPPIE was calculated (Alanis‐Lobato *et al*, 2017).

#### Classical Gene Ontology term enrichment

Classical GO term enrichment was performed using a hypergeometric analysis to identify enriched functional annotations. Protein counts were normalized using UniRef50 clusters. *P*‐values were corrected for multiple hypothesis testing using Benjamini–Hochberg correction. The *P*‐value was calculated as:

$$\text{Enrichment}=E=\frac{\left(m/n\right)}{\left(M/n\right)}$$

$$P\left(x>m\right)=f\left(m,N,n,M\right)=\sum_{i=m+1}^{\min(M,\;n)}\frac{\left(\begin{array}{c} M\\ i \end{array}\right)\left(\begin{array}{c} N‐M\\ n‐i \end{array}\right)}{\left(\begin{array}{c} N\\ n \end{array}\right)}$$

where *m* is the number of peptide‐containing proteins annotated with the GO term, *n* is the number of proteins annotated with the GO term in the human proteome, *M* peptide‐containing proteins, and *N* is the number of proteins in the human proteome.

#### Interactome enrichment

The interactome enrichment analysis was performed as a classical GO term enrichment. The enrichment was analyzed using sets of proteins known to interact with a given protein in place of sets of proteins annotated to a GO term. Interaction data were retrieved from the HIPPIE database (Alanis‐Lobato *et al*, 2017).

##### Shared GO terms analysis

The shared annotation was performed as defined in Krystkowiak *et al* (2018). The Shared GO terms analysis calculates the likelihood of any two proteins in the proteome sharing a given GO term chance.

$$p=\frac{N_{g}\;*\;\left(N_{g}‐1\right)}{N\;*\;N‐1}$$

where *N_g_* is the number of proteins with a given term in the proteome, and *N* is the number of proteins in the human proteome.

#### Comparison of results from HD2 P8 selections to selections against the sublibraries and HD2 P3

Pairwise comparison between the high/medium confidence peptides generated from selections against the HD2 P8 library and the rest of the libraries (HD2 P3 and HD2 P8 sublibraries) was made to evaluate how the results of selections against each distinct library compares to the results from selections against the HD2 P8 library. The comparison measured the extent to which selections against each library discovered known instances belonging to the *ProP‐PD motif benchmarking dataset* (Dataset EV5; http://slim.icr.ac.uk/data/proppd_hd2_pilot). The performance of each library was defined by two values, Recall and Precision. The recall was defined by the number of previously validated instances from the benchmarking set rediscovered in the high/medium set of ligands generated by selections against each library. The same benchmarking set was used to measure recall for HD2 P8 and the compared library. The precision was defined by the number of previously validated instances from the *ProP‐PD benchmarking dataset* found in the medium/high confidence set of ligands in comparison to the total number of medium/high confidence ligands.

#### Purification of KEAP1 Kelch, KPNB1 HEAT, and MDM2 SWIB for affinity measurements

The protein coding regions (Dataset EV2) were subcloned using the *EcoRI* and *NcoRI* restriction site into in pETM41 (EMBL) for the expression of 6‐His‐MBP‐tagged proteins or in the pETM33 (EMBL) vector.

1. Protein expression was induced at OD_600_ 0.8 with 1 mM IPTG, and allowed to proceed at 18°C for 20 h.
2. The bacteria were harvested and lysed under the same conditions as described above.
3. The supernatant was batch purified using the IMAC technique with Ni Sepharose 6 Fast Flow (Cytiva).
4. After 1 h incubation with the gel slurry, the gel was transferred into a column.
5. The beads were washed with wash buffer (20 mM NaPO_4_, 0.5 M NaCl, 30 mM imidazole pH 7.4) and eluted with elution buffer (20 mM NaPO_4_ 0.5 M, NaCl 500 mM imidazole, pH 7.4). The proteins were further purified as follows:
  - 14‐3‐3 SFN: The protein was not eluted from the IMAC column. Instead, after washing unbound protein away, His‐tagged HRV3C protease was added and incubated for 16 h in 20 mM NaPO_4_, 0.5 M NaCl, pH 7.4. The cleaved protein was eluted with the same buffer. The cleaved protein was dialyzed into 50 mM KPO_4_ pH7.5 for 16 h.
  - TLN1 PTB was cleaved with His‐tagged HRV3C protease while dialyzing into 50 mM KPO_4_ pH 7.5 for 16 h 4°C. The cleaved protein was applied on a Ni^2+^ IMAC gel and incubated for 1 h at 4°C to remove the tag. The protein was once more dialyzed in 50 mM KPO_4_ pH 7.5 for 16 h.
  - MDM2 SWIB: The protein was cleaved from the His‐GST tag using His‐tagged HRV3C protease and then further purified with a S100 HiPrep 16/60 Sephacryl gel filtration using 150 mM NaCl, 20 mM NaPO_4_ pH 7.4. After gel filtration the protein was dialyzed into 50 mM NaPO_4_ pH 7.5 for 16 h.
  - KPNB1 HEAT: The eluted protein was cleaved with His‐tagged TEV protease while dialyzing in 150 mM NaCl, 50 mM Tris, 0. 5 mM EDTA, 1 mM 1,4‐Dithiothreitol (DTT) pH 8.0 for 16 h. The cleaved tag and the protease were removed through a reverse Ni^2+^ IMAC. The protein was dialyzed in 50 mM KPO_4_ pH 7.5, 1 mM DTT for 16 h.
  - KEAP1 Kelch: The MBP‐tagged domain was dialyzed into 50 mM KPO_4_ pH 7.5 for 16 h.
  - KPNA4 ARM: The MBP‐tagged domain was further purified through size exclusion chromatography using a Sephacryl S300 high‐resolution 26/600 and 50 mM KPO_4_ pH 7.5 as running buffer.

#### Fluorescence polarization

Fluorescence polarization affinity measurements were carried out with an iD5 multidetection plate reader (Molecular Devices) using Corning assay 96‐well half area black Flat‐bottom Nonbinding surface plates (Corning, USA #3993). The settings were 485 nm excitation and 535 nm for emission at a reading height of 1.76 mm and total volume of 50 µl. Peptides were obtained from GeneCust (France) at > 95% purity. Unlabeled peptides were dissolved in 50 mM KPO_4_ or 50 mM NaPO_4,_ pH 7.5. FITC‐labeled peptides were dissolved in dimethyl sulfoxide (DMSO). Protein for saturation experiments, or peptides for the displacement experiments, were arrayed in serial dilution in 50 mM KPO_4_ pH 7.5 in 25 µl, followed by addition of 25 µl of a master mix. For saturation binding experiments, the master mix contained 2 mM DTT and 10 nM FITC‐labeled peptide in 50 mM KPO_4_ pH 7.5 or 50 mM NaPO_4_ pH 7.5. For competition experiments, the master mix was supplemented with the protein of interest at a concentration of four times the *K*_D_ value. Data were analyzed with GraphPad Prism version 9.0.0 for MacOS (GraphPad Software, San Diego, California USA, www.graphpad.com). For direct binding, we used a quadratic equation with *pept* indicating the fixed probe peptide concentrations, *X* indicating the protein concentration, the constant *A* being the signal amplitude divided by probe peptide concentration, and *B* is the plateau value:

$$Y=A*\frac{\text{pept}+X+K_{D}‐\sqrt{{\left(\text{pept}+X+K_{D}\right)}^{2}‐4\;*\;\text{pept}\;*\;X}}{2}+B.$$

For the FP competition experiments data were fitted to a sigmoidal dose–response (variable response; GraphPad Prism).

#### Cloning and mutagenesis for analysis of NLSs

For the cell‐based NLS experiment, a pEGFP‐C1 vector was modified to contain two additional eGFP genes spaced by a linker region on the 3′ end of the vector's multiple cloning site (Appendix Fig S8). The modified vector is here called a “tri‐GFP vector”. DNA strands for tandem peptides to be tested for NLS function were constructed by PCR using overlapping primers that coded for the peptide sequences and also contained overlapping regions for the MCS in the tri‐GFP vector. The PCR products were then cloned into the tri‐GFP vector using Gibson Assembly® (New England Biolabs) following the manufacturer's recommendations. Obtained clones were verified by Sanger sequencing.

NLS experiments using full‐length target proteins were carried out using mCherry (Addgene plasmid #54563)‐tagged proteins. Briefly, DNA strands of full‐length proteins were amplified using primers containing 5′ overhangs matching MCS of the target vector. The PCR products were later cloned into the mCherry2‐C1 vector using Gibson Assembly® (New England Biolabs) following the manufacturer's recommendations. The putative NLS site in each construct was mutated by site‐directed mutagenesis to confirm the functional NLS sequence. All obtained clones were verified by Sanger sequencing.

#### Cell culture and samples preparation

HEK293 cells were obtained from Sigma (Cat. 85120602) and cultured using DMEM with GlutaMAX™ Supplement (Gibco™) supplemented with 10% FBS (Gibco™) and Non‐Essential Amino Acids Solution (NEAA, Gibco™). Cultures were maintained at 37°C with 5% CO_2_ in humidified chambers and were routinely checked for mycoplasma contamination. For NLS experiments, cells were transfected using FuGENE® HD (Promega) according to manufacturer's instructions and using highly purified DNA samples.

1. Cells were then grown in eight‐well chamber slides. Following fused GFP or mCherry expression for 36 h post‐transfection, cells were washed with ice‐cold PBS and were fixed using Image‐iT™ Fixative Solution containing 4% formaldehyde (Invitrogen™) for 15 min on ice.
2. Cells were washed three times with PBS each for 5 min at room temperature. Slides were dried and mounted with ProLong™ Glass Antifade Mountant with NucBlue™ Stain (Invitrogen™).

#### Microscope image acquisition and processing

Images were acquired by Zeiss imager Z2 microscope using C11440 camera (Hamamatsu) and 40× oil objective lens (N.A. 1.4) using Zen software (V3.2, blue edition). HXP 120 V light source unit was used for sample excitations using fixed light intensities across all samples and images collected using appropriate filter sets. Merged images of 8‐bit depth were exported and processed in ImageJ, where color brightness was adjusted for each channel homogeneously for all the images.

#### Disease‐relevant SNPs analysis

PepTools SNP annotation of the high/medium confidence peptides was analyzed. Data were filtered to create a “disease‐relevant” SNP dataset based on clinical significance annotation (“Pathogenic,” “Likely Pathogenic,” “Disease,” ”Risk factor,” “Association,” “Protective,” “Drug response,” “Affects”). These “disease‐relevant” SNPs were mapped to key specificity determinant residues (based on the defined positions in the ELM consensus for the given bait) and the two flanking residues. The output was then used to build a PPI network for each bait (Dataset EV10). In total, we constructed 16 networks, where each bait is binding at least to one peptide with a mutation. A network composed of only peptides with disease‐associated mutations affecting motif‐encoding residues was also built and visualized using Cytoscape (Appendix Fig S9).

#### Phosphorylation analysis

PepTools phosphorylation site annotation of the high/medium confidence peptides was analyzed (Dataset EV11). Phosphorylation sites were mapped to key specificity determinant residues (based on the defined positions in the ELM consensus for the given bait) and the two flanking residues. Phosphosite information was added to the disease‐associated mutations PPI networks (Appendix Fig S9).

#### Plots and visualization

Graphs were created using the Matplotlib (Hunter, 2007) and Seaborn (Waskom *et al*, 2017) libraries in Python 3 (Van Rossum & Drake, 2009), or with ggplot2 library (Wickham, 2016) in the R scripting language (R Core Team, 2015). Structure figures were created with PyMOL. All networks were visualized using Cytoscape (Shannon *et al*, 2003).

## Author contributions

**Caroline Benz:** Validation; Investigation; Writing – original draft; Writing – review & editing. **Muhammad Ali:** Investigation; Writing – review & editing; Methodology. **Izabella Krystkowiak:** Software; Methodology; Writing – review & editing. **Leandro Simonetti:** Investigation; Visualization; Writing – original draft. **Ahmed Sayadi:** Investigation; Visualization. **Filip Mihalic:** Investigation. **Johanna Kliche:** Investigation. **Eva Andersson:** Investigation. **Per Jemth:** Formal analysis; Supervision; Funding acquisition. **Norman Davey:** Conceptualization; Data curation; Software; Supervision; Funding acquisition; Investigation; Visualization; Methodology; Writing – original draft; Writing – review & editing. **Ylva Ivarsson:** Conceptualization; Data curation; Supervision; Writing – original draft; Project administration; Writing – review & editing.

In addition to the CRediT author contributions listed above, the contributions in detail are:

CB, MA, FM, EA, and JK purified proteins. CB and MA constructed phage libraries. CB and JK performed phage selections. CB, FM, and JK performed affinity measurements and analyzed the data. MA performed cell‐based validations. LS built an NGS data analysis pipeline. IK constructed the PepTool website. ND and LS processed ProP‐PD data with support of IK. ND and AS benchmarked the ProP‐PD data. ND designed the custom oligonucleotide libraries with support of LS. CB, MA, JK, FM, PJ, ND, and YI designed experiments. ND and YI conceived the study. CB, AM, IK, LS, AS, PJ, ND, and YI wrote the paper with input from all authors.

## Supporting information



Appendix

Dataset EV1

Dataset EV2

Dataset EV3

Dataset EV4

Dataset EV5

Dataset EV6

Dataset EV7

Dataset EV8

Dataset EV9

Dataset EV10

Dataset EV11

Source Data for Appendix

Source Data for Figure 4

Source Data for Figure 5

Source Data for Figure 6

## Data Availability

The ProP‐PD interaction data are available in the Datasets EV provided with this manuscript. Results are also available online at http://slim.icr.ac.uk/data/proppd_hd2_pilot. An interactive website to explore the full library design is available at http://slim.icr.ac.uk/phage_libraries/human/. The PepTools analysis tool is available at http://slim.icr.ac.uk/tools/peptools/. The datasets and computer code produced in this study are/will be available in the following databases:
‐ Computer scripts for demultiplexing and analysis of the sequences: https://bitbucket.org/daveylab/phage_display_pipeline/. ‐ PPI data: The protein interactions from this publication have been submitted to the IMEx (http://www.imexconsortium.org) consortium through IntAct (Orchard *et al*, 2014) and assigned the identifier IM‐29361.
