# Supplementary material for: Proteome‐scale mapping of binding sites in the unstructured regions of the human proteome
Source: Mol Syst Biol. 2022 Jan 19;18(1):e10584. doi: 10.15252/msb.202110584 (PMC8769072; doi:10.15252/msb.202110584)
Supplement: Supplementary file 1 — Appendix [file MSB-18-e10584-s011.pdf]

Appendix

Proteome-scale mapping of binding sites in the unstructured regions of the human proteome

Caroline Benz[1,*], Muhammad Ali[1,*], Izabella Krystkowiak[2,*] , Leandro Simonetti[1], Ahmed Sayadi[1], Filip Mihalic[3], Johanna Kliche[1], Eva Andersson[3], Per Jemth[3], Norman E. Davey[2,#], Ylva Ivarsson[1, #]

Affiliations:

1. Department of Chemistry - BMC, Uppsala University, Box 576, Husargatan 3, 751 23 Uppsala, Sweden
2. Division of Cancer Biology, The Institute of Cancer Research, 237 Fulham Road, London SW3 6JB, UK.
3. Department of Medical Biochemistry and Microbiology, Uppsala University, Box 582, Husargatan 3, 751 23 Uppsala Sweden

Table of content

Figure S1. Non-design components and count distribution of the libraries.

Figure S2. Representative structures of the bait proteins domains with bound ligands.

Figure S3. Distribution of NGS reads for binding enriched phage pools.

Figure S4. Analysis of the proportion of motif-containing peptides and validation of low affinity interactions of peptides lacking consensus motifs.

Figure S5. Overview of GO term enrichment analysis.

Figure S6. Analysis of correlation between affinity and ProP-PD results.

Figure S7. FP affinity measurements of KPNA4.

Figure S8. Validation of KPNA4 binding sequences as NLSs.

Figure S9. Additional PPI networks based on peptides with reported disease-associated mutations or phosphosites.

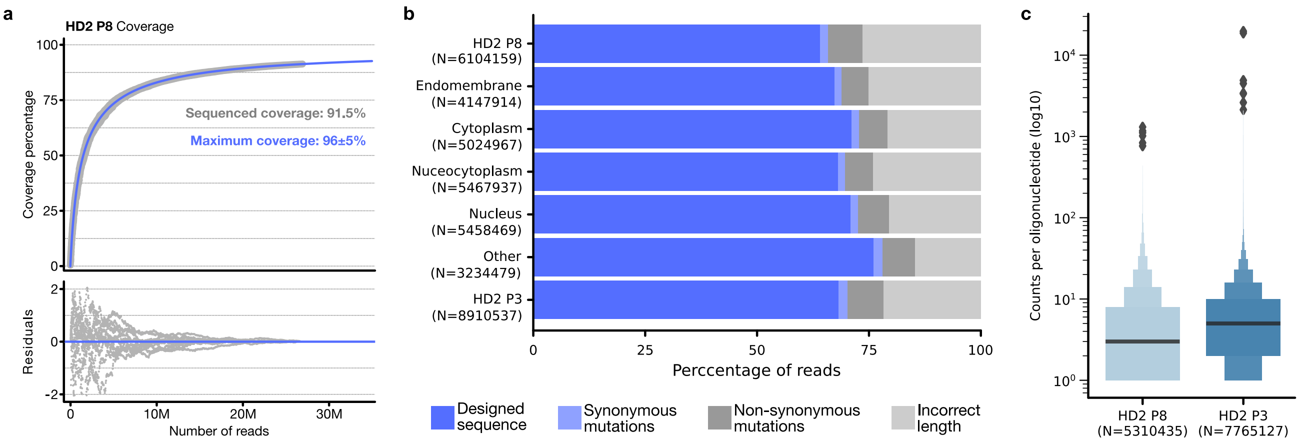

Figure S1. Non-design components and count distribution of the libraries. (a) Coverage of the HD2 P8 library as a function of sequencing depth, fitting of data to a double hyperbolic model (in blue) and residuals are shown. The contribution of reads of all sub-libraries were considered for calculating the coverage of the HD2 P8 library, as it is the result of the balanced combination of all compartment-specific sub-libraries. (b) Percentages of reads obtained by NGS of the libraries generated in this work associated with sequences that matched the designed sequences, those that presented mutations either silent or non-silent, and those that due to frame-shifting, insertion or deletion mutations did not match the expected oligonucleotide length. (c) Distribution of NGS counts for the designed oligonucleotides in the HD2 P8 and HD2 P3 libraries.

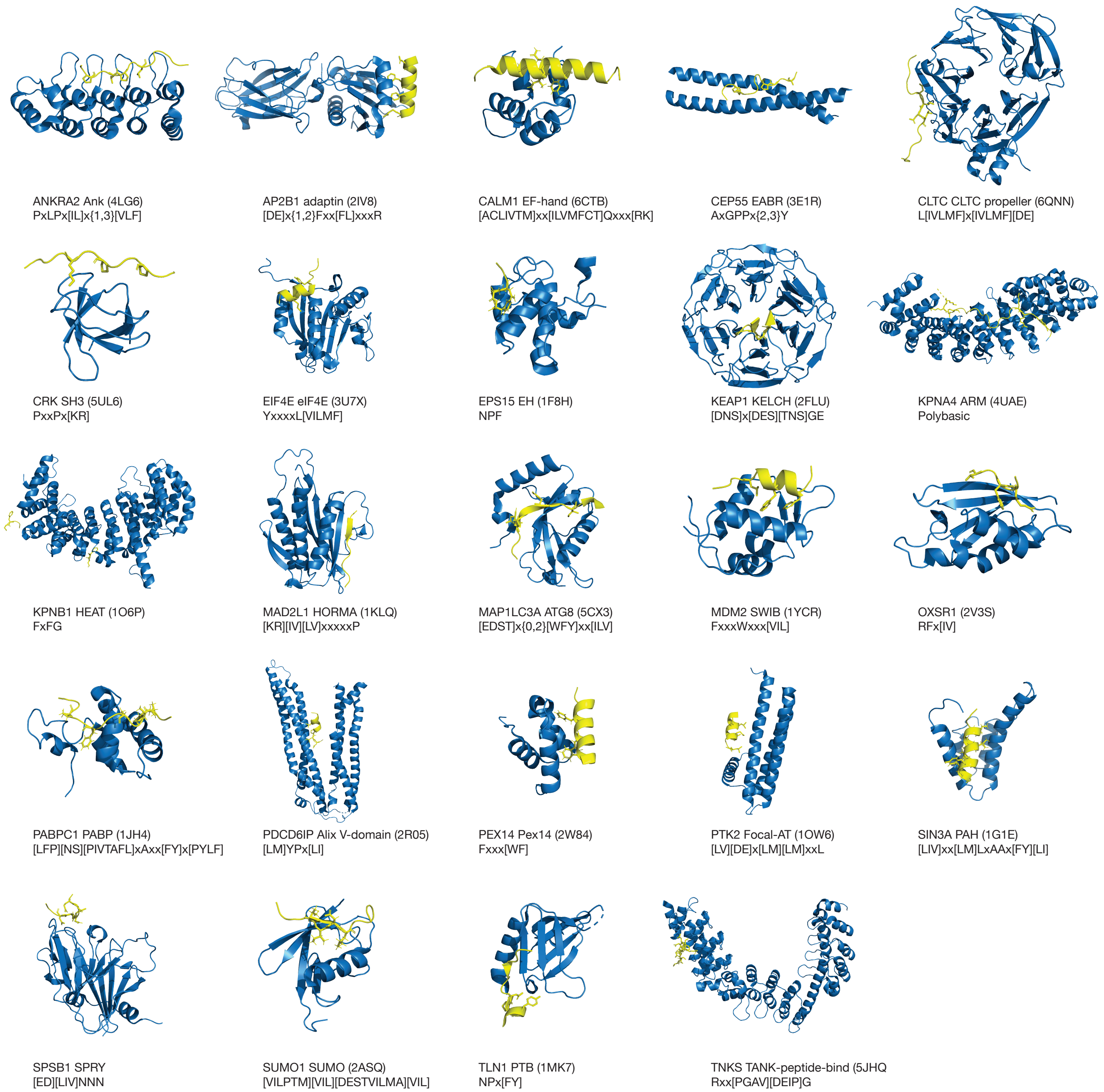

Figure S2. Representative structures of the bait proteins domains (blue) with bound ligands (yellow) and indicated binding motifs. PDB codes are indicated in the figure.

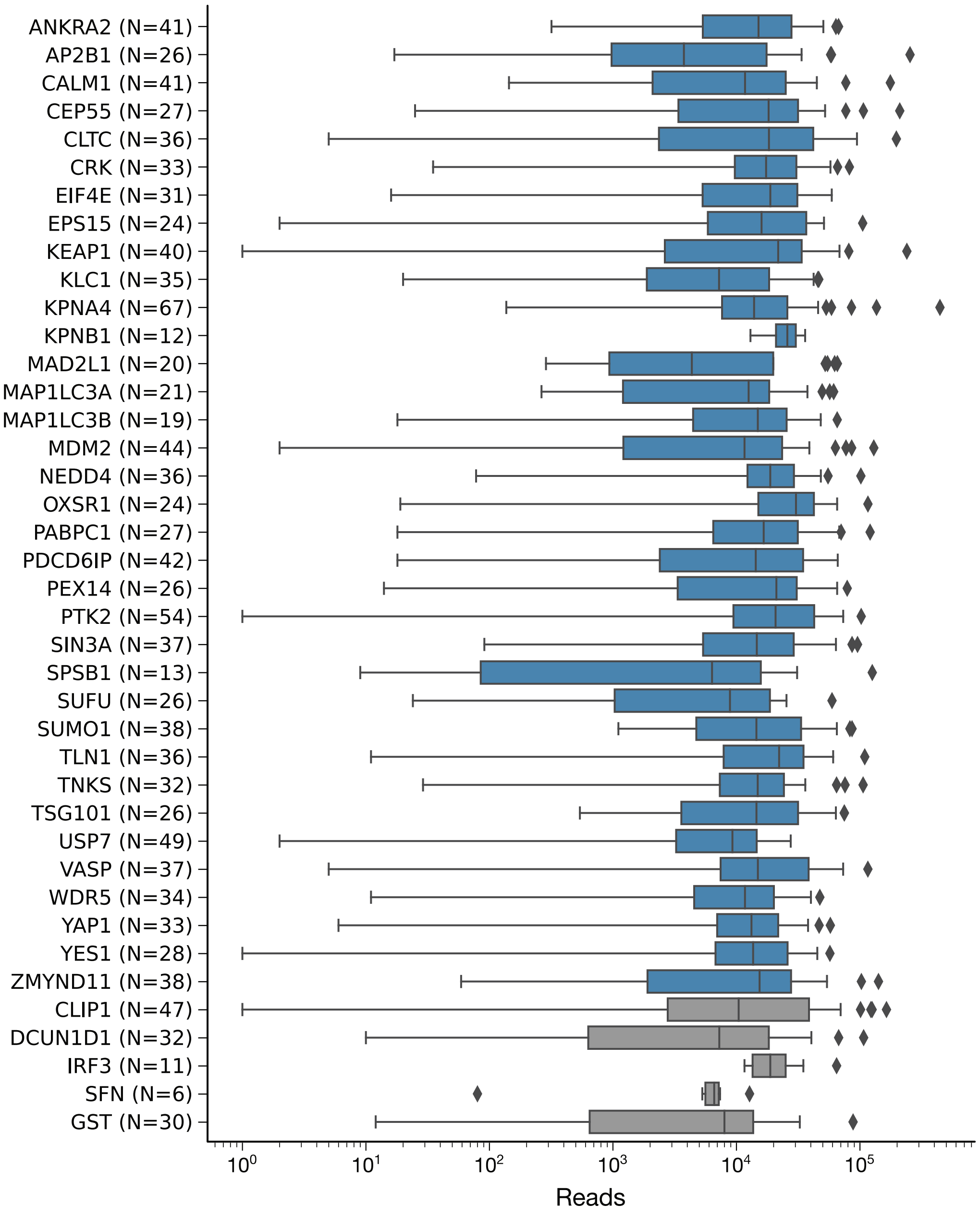

Figure S3. Distribution of NGS reads for binding enriched phage pools. Distribution of the NGS reads that matched the peptides encoded by the library are shown grouped by bait. Control baits are shown in gray. N = number of barcoded phage pools included in the analysis per bait.

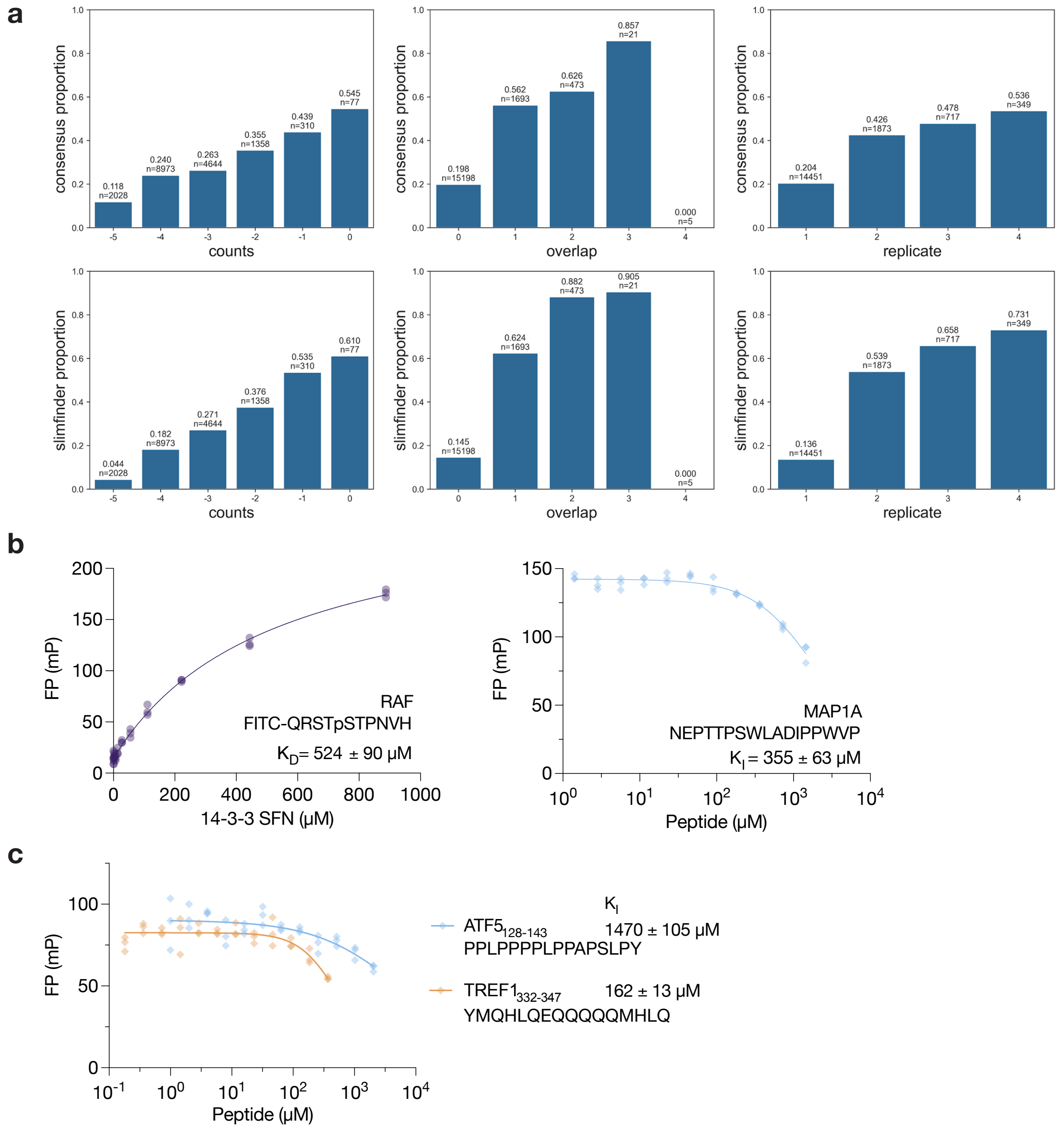

Figure S4. Analysis of the proportion of motif-containing peptides (a) and validation of low affinity interactions of peptides lacking consensus motifs (b-c).
(a) Proportion of peptides returned for a bait containing the expected ELM consensus for the bait (consensus proportion) or the SLiMFinder consensus enriched in the peptides in the screen of that bait (slimfinder proportion). The plots show the data grouped by three peptide metric statistics: count - $\log^{10}$ of the normalized counts, overlap - the number of overlapping peptides returned in the screen for that bait, replicates - the number of replicates that the peptide was returned. (b) FP affinity measurements of the interactions between 14-3-3 SFN and the probe peptide phospho-RAF1255-264 (FITC-QRSTpSTPNVH; left) and the competition with unlabeled MAP1A$_{1836-1851}$ (right). (c) FP competition experiment for the Keap1 KELCH domain between the FITC-labeled probe peptide NFE1L1$_{226-243}$ and the unlabeled peptides ATF$_{128-143}$ and TREF$_{223-347}$ that lack Keap1 KELCH binding motif.

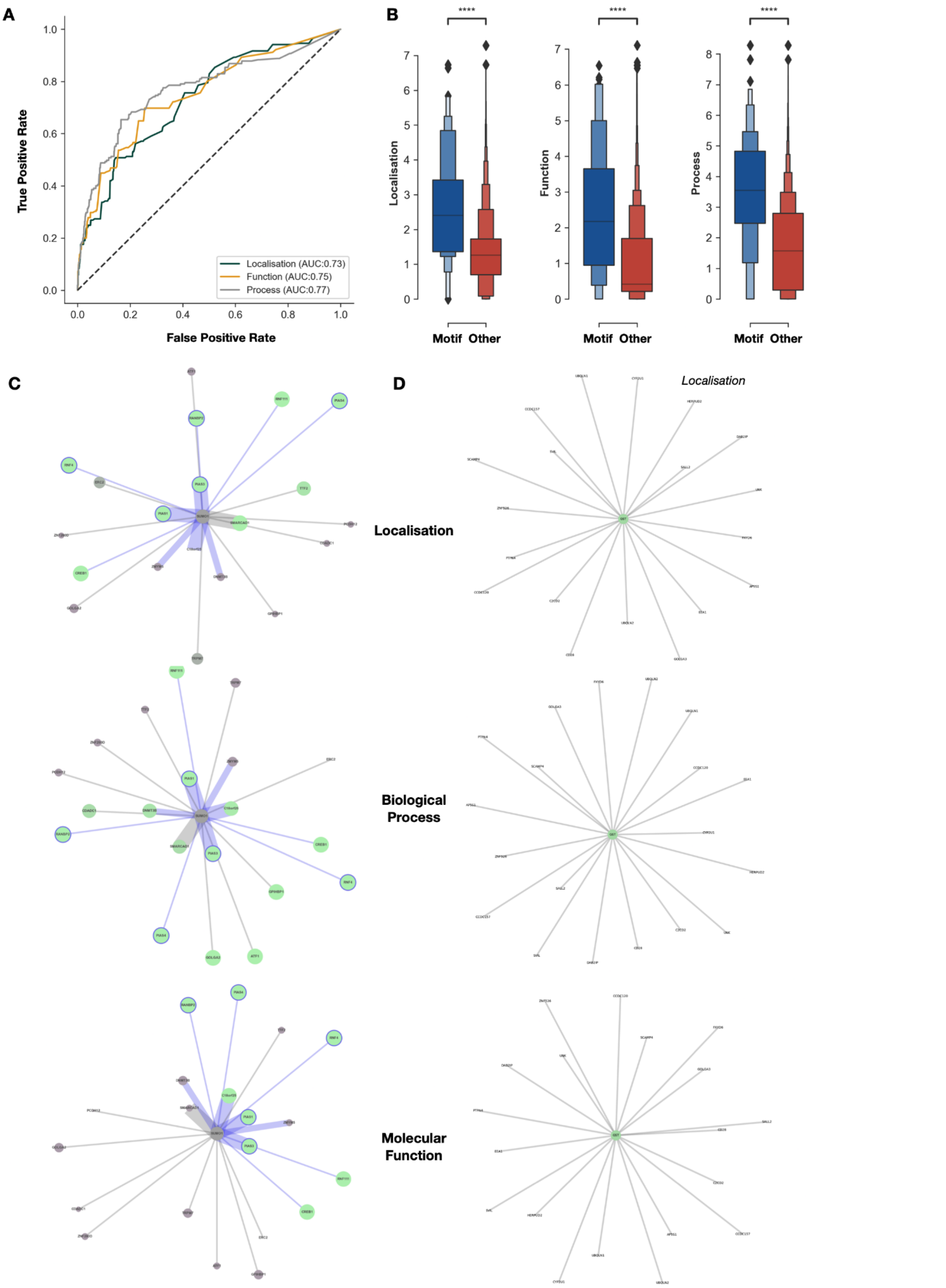

Figure S5. Overview of GO term enrichment analysis. (A) ROC curve showing the discriminator power of the shared GO terms analysis metrics for validated motifs (B) Box plots of Localisation, Biological Process and Molecular Function GO term enrichment in validated motif containing peptides and other returned peptides. (C-D) Network of representation of the SUMO (C) and GST (D, control) bait interactions with high/medium confidence peptide-containing proteins. Network is overlaid with data from a shared GO terms analysis. Three networks are displayed for each bait: Localisation, Biological Process and Molecular Function. Node colour and size is the p-value of the best shared ontology term (greener and larger nodes are more enriched). Edge weights and lengths represent the best peptide confidence for the protein (more confident peptides are closer to the bait with thicker edges). The edges of known interactors are shown in blue. Nodes representing validated motif-containing proteins are ringed in blue.

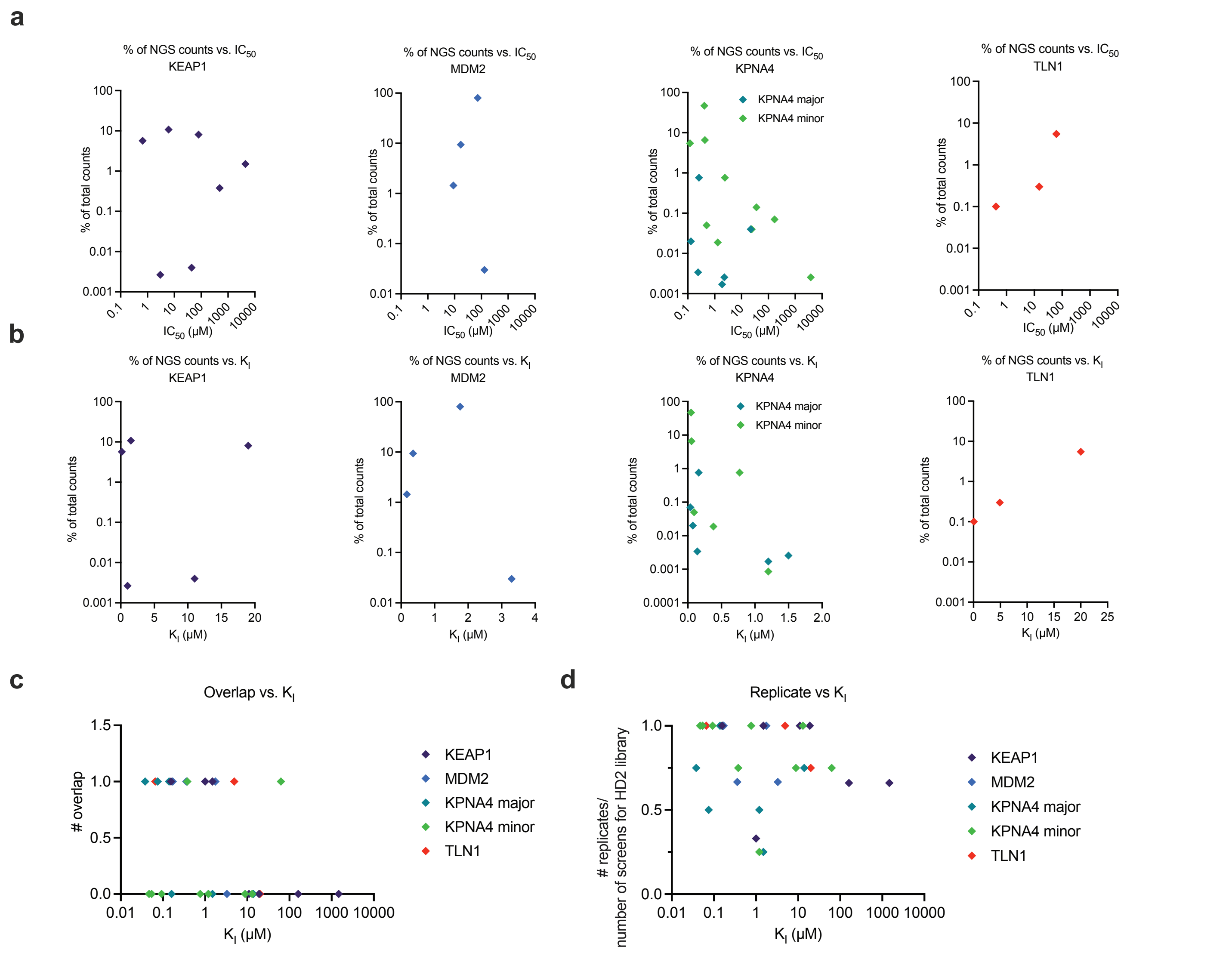

Fig S6. Analysis of correlation between affinity and ProP-PD results. The % NGS counts per were plotted against (a) IC_{50} values, and (b) K_I values. The affinities (K_I values) were further plotted against (c) the observed overlaps of the peptides in selections, and (d) the occurrence of peptides in replicate selections. A KPNA4 outlier was removed for better visualization.

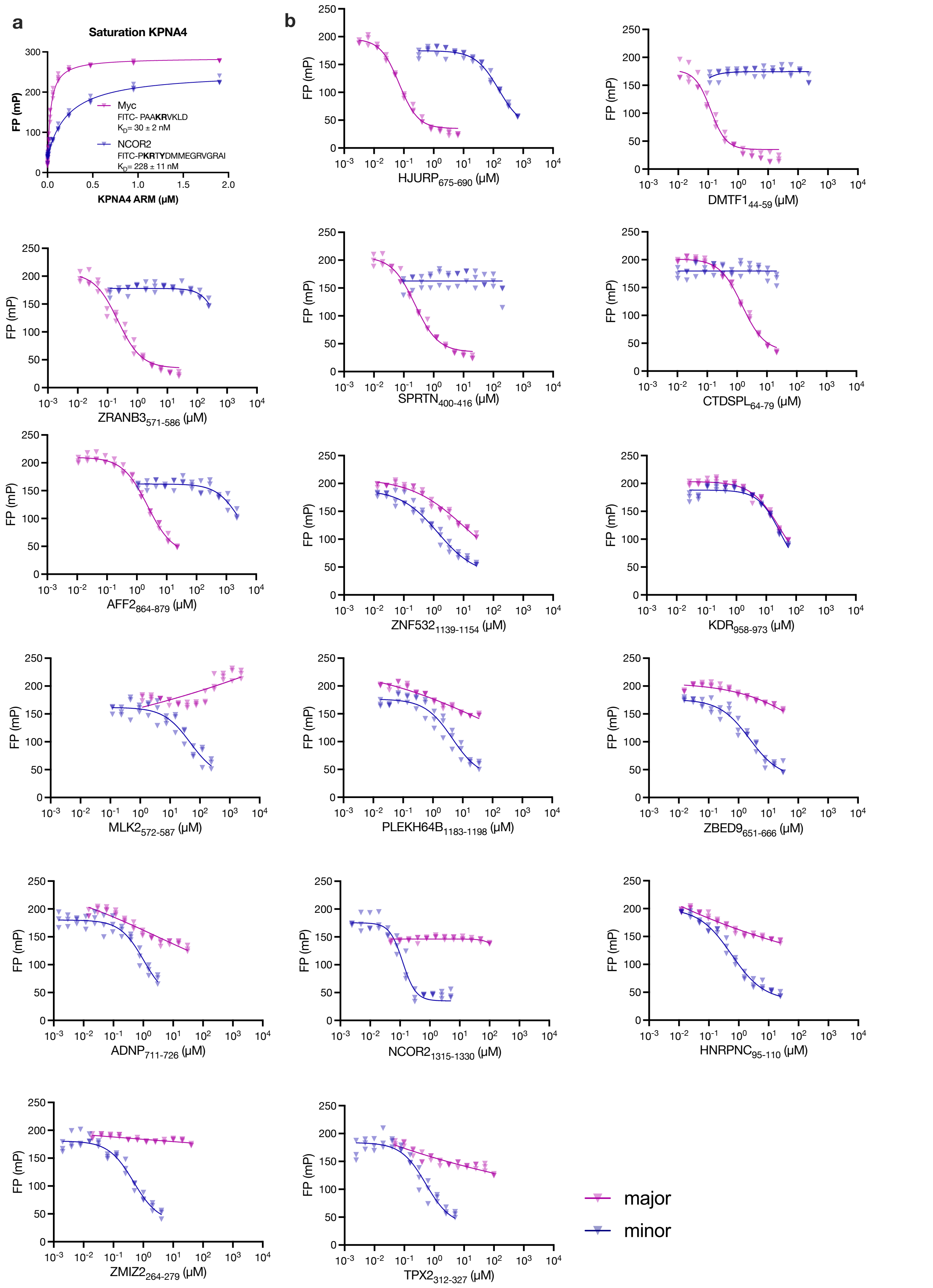

Fig S7. FP affinity measurements of KPNA4. (a) Saturation curves with probes for the for the major groove (Myc$_{320-328}$ FITC-PAAKRVKLD; purple) and minor groove (NCOR2$_{1307-1322}$; FITC-PKRTYDMMEGRVGRAI (blue)). (b) FP competition binding experiment of 18 peptides against the major groove probe FITC-Myc$_{320-328}$ (purple) and the minor groove FITC-NCOR2$_{1307-1322}$ (blue). Unlabeled peptides used for competition: HJURP$_{675-690}$, DMTF1$_{44-59}$, ZRANB3$_{571-586}$, SPRTN$_{400-416}$, CTDSPL$_{64-79}$, AFF2$_{864-879}$, ZNF532$_{1139-1154}$, KDR$_{958-973}$, MLK2$_{572-587}$, PLEKH64B$_{1183-1198}$ ZBED9$_{651-666}$, ADNP$_{711-726}$, NCOR2$_{1307-1322}$, HNRPNC$_{95-110}$, ZMIZ2$_{264-279}$, TPX2$_{312-327}$.

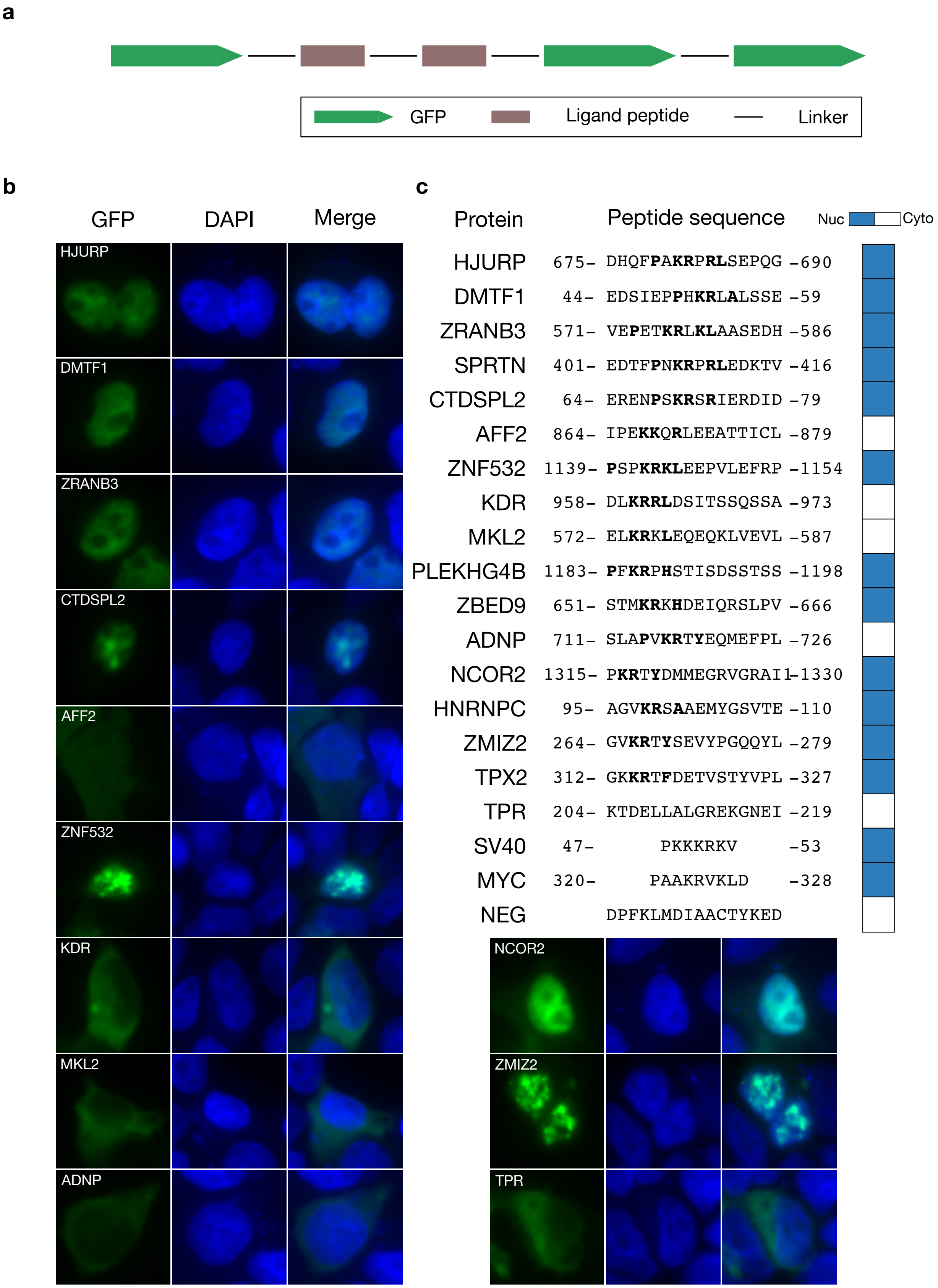

Fig S8. Validation of KPNA4 binding sequences as NLSs. (a) Schematic of the construct used as NLS sensor. (b) Cellular localization of NLS sensor with different peptides inserted. HEK293 cells were transiently transfected with the NLS sensor and fixed 36 h after transfection, and imaged using epifluorescence microscopy. The nucleus was stained with DAPI. (c) Overview of the peptide sequences fused to the NLS sensor, with the resulting cellular localization indicated.

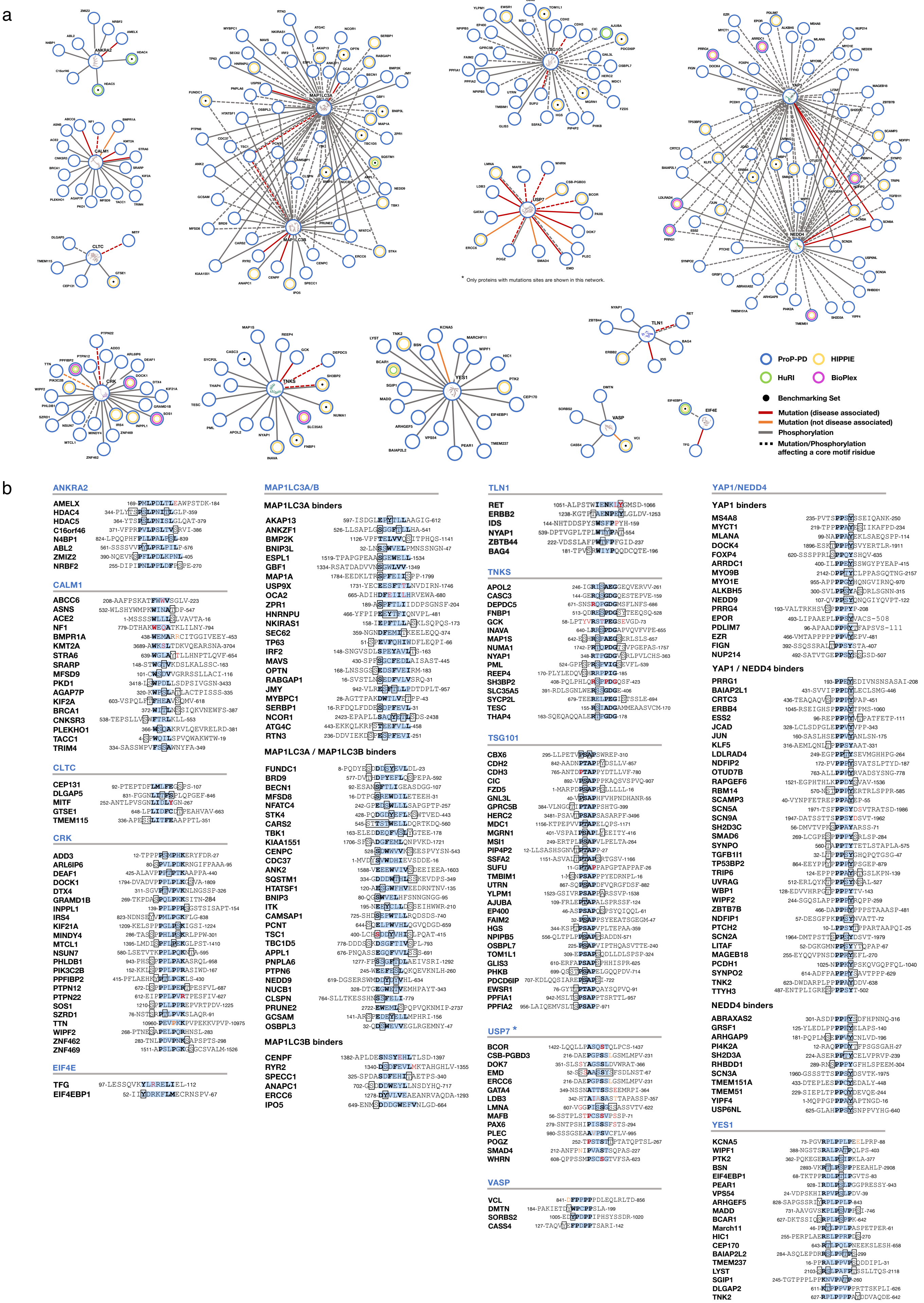

Fig S9. Additional PPI networks based on peptides with reported disease-associated mutations or phosphosites. (**a**) Networks showing interactions of reproducibly selected high/medium confidence peptides with overlapping disease-associated mutations or phosphorylation. Peptides with mutations or phosphorylation residues occurring in the core motif or in the flanking regions (+/- 2 residues) are shown. Mutations associated with diseases are colored in red (orange if they are not disease associated). Phosphorylated sites are colored in gray. Dashed-edge lines represent core motif residues holding a mutation or a phosphorylation site. (**b**) Sequences of high/medium confidence peptides with disease-associated mutations in the binding motif. Motif-containing regions are highlighted with blue background, core motifs are indicated in bold letters, phosphorylation sites within or in vicinity of the motifs are indicated by a box, and disease associated mutations are indicated in red bold letters (mutation of core residue).
