# Supplementary material for: Proteome‐scale mapping of binding sites in the unstructured regions of the human proteome
Source: Mol Syst Biol. 2022 Jan 19;18(1):e10584. doi: 10.15252/msb.202110584 (PMC8769072; doi:10.15252/msb.202110584)
Supplement: Supplementary file 17 [file MSB-18-e10584-s017.pdf]

Proteome-scale mapping of binding sites in the unstructured regions of the human proteome

Caroline Benz, Muhammad Ali, Izabella Krystkowiak, Leandro Simonetti, Ahmed Sayadi, Filip Mihalic, Johanna Kliche, Eva Andersson, Per Jemth, Norman Davey, and Ylva Ivarsson
DOI: 10.15252/msb.202110584

Corresponding author(s): Ylva Ivarsson (ylva.ivarsson@kemi.uu.se) , Norman Davey (norman.davey@icr.ac.uk)

Review Timeline:

Submission Date:	18th Jul 21
Editorial Decision:	10th Aug 21
Revision Received:	11th Nov 21
Editorial Decision:	13th Dec 21
Revision Received:	21st Dec 21
Accepted:	22nd Dec 21

Editor: Maria Polychronidou

Transaction Report:

(Note: With the exception of the correction of typographical or spelling errors that could be a source of ambiguity, letters and reports are not edited. Depending on transfer agreements, referee reports obtained elsewhere may or may not be included in this compilation. Referee reports are anonymous unless the Referee chooses to sign their reports.)

Thank you again for submitting your work to Molecular Systems Biology. We have now heard back from the three referees who agreed to evaluate your study. Overall, the reviewers acknowledge that the study is a relevant contribution to the field. They raise however a series of concerns, which we would ask you to address in a revision.

I think that the reviewers' recommendations are clear and therefore it is not required to repeat the points listed below. During our cross-commenting process, in which the reviewers get the chance to make additional remarks after reading all other reports, reviewer #3 has made some additional (still supportive) comments, which I have pasted below for your reference. All issues raised by the reviewers need to be satisfactorily addressed. Please contact me in case you would like to discuss in further detail any of the issues raised.

On a more editorial level, we would ask you to address the following points:

REFEREE REPORTS

Reviewer #1:

Review of Benz et al "Proteome-scale footprinting of binding sites in the unstructured regions of the human proteome"
The manuscript describes a phage display based protein interaction screen / method that involves as bait 34 proteins defined by domains that are known to recognize linear amino acid epitopes. The peptide library (HD2) is designed from disordered human protein stretches in a tiling fashion with 4 amino acids overlap each. The library thus covers a difficult to study subset of potential binding interfaces, not displaying free N-terminal or C-terminal residues, and no PTMs modifications. A substantial H2D-P8screen is performed and read out through second gen sequencing. Four criterial for scoring binding peptides are established, reproducibility, peptide overlap, motif overlap, and read count. 396 / 2653 high/ medium peptides are defined as mediators of PPIs, which is about 2000 human protein-protein interactions. The data are benchmarked against a 337 literature motif data set, de novo motifs and known motifs are compared. Furthermore benchmarking against two major Y2H, AP-MS data sets and the literature meta data set HIPPIE are described, including the variation between different baits, exemplary for NEDD4 and PABPOC1. For a set of 4x4 interactions pairs KDs are determined showing a broad range of affinities up to 100 µM. The possibility to capture these low affinity interactions is a clear advantage of the approach. After a short methodological account
(mostly negative results) KPN4A motif binding is studied in much detail, revealing that a large fraction of the peptides with motif matches represent NLS. Accordingly, R/K to AA prevent nuclear import in selected cases. A final very interesting analysis is performed: Amino acid mutations and PTMs are mapped on to the peptide binding sequences to predict interaction perturbation events, e.g. through phosphorylation or amino acid substitutions. Exemplary four substitutions and one phosphorylation is shown to substantially decrease the affinity for KPNA4 or KEAP1 respectively.
This is a prime study on motif-based protein interactions. The assay is addressing linear epitope (::motif) binding of specific protein domains and the authors do a very thorough and innovative approach on defining the motifs that contribute to the interaction.

However, a maximum of 6.8% (27/396 in the high confidence and lower 74/2653 in the medium confidence or 65/3049) it the fraction of the binding that is explained through the motifs. In other words, the majority of the binding events is not explained by motifs (even if the final number because of de novo motifs etc. is not clear). All analysis focuses on this minor subset and the majority of the binding events is ignored. As far as I can grasp there is the one case, the DLIFTDSKLYIPLE peptide of TPTE2 which does not contain a motif and is assayed in figure 3 and it binds (of course) with intermediate affinity! What is the contribution of the motif binding peptides to the overall data, and how to think about the non-motif linear epitopes. With reference to the wording in the text about the hidden interactome, it feels as if the authors are creating another hidden layer through ignoring most of the data in their analyses.
Along the same line, the authors as the state "present a resource of more than 2,000 human PPIs with amino acid resolution of binding sites" but do not analyze the data in biological terms at all. In this respect the data set does not compare well to other analysis that perform screens at this scope.

Points for consideration:
*) The whole part about dividing the library into subcellular localization bins is not clear to me. Attempts to reduce the complexity of the peptides display make sense in principle, however were shown to compare similar to the full H2D-P8 screen. This is an in vitro system therefore it did not become very clear what the particular subsets according to subcellular localization could possibly bring.
*) Figure 1 g, what about displaying the number of partner peptides found for each domain. The percentages and more so -log p values are sometimes misleading. What about percent peptides bound with motif vs other epitope binders. Some counts according to domains would allow for a better data overview. How do PPIs distribute across the 35 baits? A PPI summary is somehow missing/ would be useful for a better understanding of the screening results.
*) Peptide scoring: two remarks: i) the specificity determinant score (similarity of the peptides to SLiMFinder motifs) performs better than the final score (all four metrices score) against the benchmark. ii) Affinities do not correlate with read count, so why does read count as metric #4 perform well?
*) "The medium/high confidence HD2 P8 data has twice the recall (the proportion of PPIs that have been rediscovered) of BioPlex and HuRI on the motif-based interactions set, but with lower precision (the proportion of re-discovered PPIs among all PPIs found), particularly when compared to the HuRI data. " This sentence is confusing and makes only sense if the comparison refers to HuRI vs BioPlex. Hence the precision is twice as high for HuRI in this comparison. I thought it is well established that Y2H data better resemble liner epitope mediated PPIs (c.f. ELM) and I think that is what the authors want to say.
*) Figure 2l: N for the three data sets are missing / confusing. Based on Protein overlap, how many PPIs can be found / were found.
*) It would be much easier to present the four peptide sequences assayed in Figure 3C.

*) It would also be a better validation to sample peptide sequences from the set of interators including non-motif binders for validation.

*) Even a basic analysis of the PPI data as such is missing.

*) The focus on KEAP1 misses out NRF2.

Reviewer #2:

In the manuscript by Benz et al, Proteome-scale footprinting of binding sites in the unstructured regions of the human proteome, the authors present an impressively thorough description of second generation peptide phage display libraries constructed from intrinsically disordered regions of the human proteome, i.e. ProPD which they screen with 35 known SLiM-binding domains. They compare the performance of this library with other phage display libraries and with other unbiased methods (Yeast 2 hybrid, Affinity Purification coupled to mass spectrometry, AP-MS) and convincingly demonstrate the power of this approach to identify human SLiM-containing partners and discover new binding partners and new SLiMs. They also establish the affinity of some of the peptides discovered using flourescence polarization. In all this is a clearly written study and a technical tour de force that establishes the power of ProPD to systematically identify and characterize short linear motifs. The significance of the work is further demonstrated by in depth analysis of several new nuclear localization sequences (NLS) from ProPD analysis with the importin KPNA4 which also shows that these NLS sequences function within the context of the entire protein. Finally, several disease associated mutations that lie within SLiMs and alter SLiM function/affinity are identified. Overall this paper represents the 'bible' on identifying SLiMs using ProPD and adds significantly to our current knowledge of SLiMs and SLiM instances in the human proteome. As such information about this method and the resulting comprehensive information contained in this manuscript will be of interest to a broad audience. My relatively minor comments/suggestions are as follows:

1) Title. I find the word 'footprinting' in the title misleading as it implies a specific biochemical technique. I think the main point is that binding sites are revealed at the amino acid level. "Proteome-level elucidation of binding sites with amino acid resolutionin unstructured regions of the human proteome " ?

2) Table 1 presents known motifs that were identified by ProPD. However there are many differences between the ProPD motifs and the expected motifs. Authors should comment on this. First, ProPD has the power to identify a broader range of amino acids in motif positions compared to the low through put regular expression matching that is often used to define a motif. Several examples of this are seen. Second, ProPD can reveal additional contributions from 'flanking' residues again not identifiable by RegEx matching. Finally, some flanking residues in 'expected' motifs are not included in the ProPD motif. How might this occur? Authors might point out that methods which systematically assess the contribution of different amino acids to motif affinity are complementary to ProPD and can further reveal aspects of motif specificity.

3)Minor comment. On page 9 "bold residue denote residues
matching the bait consensus" should be 'matching the expected motif consensus for this bait'

4)P. 11: I found the discussion of peptides that bind to the 'phosphotyrosine binding domain' in Talin confusing: The peptides discussed contain tyrosine but it is not phosphorylated, i.e. this particular motif does not require phosphorylation?

5) on P 13 authors state that the P3 library reinforces findings from P8 library. In fact these results seem distinct and are COMPLEMENTARY. Any explanation for why the peptides discovered in P3 are not found in the P8 library? Is there some particular aspect of P8 fusions that prevents some domains from binding? Or are these peptides under represented in the P8 library relative to the P3 library?

6) P.14: how many peptides found with KPNA4 occur in proteins that are known to localize to the nucleus?

7) Finally in the discussion the authors give a strong 'disclaimer' to their results saying they are based on invitro interactions and must be tested in the context of the whole protein. While this is true, the fact that all the sequences are in disordered regions makes it likely they are functional. Also didnt they also show us a strong correlation between the PPIs identified by ProPD and using whole protein methods such as Yeast 2 hybrid and AP-MS? I think the statement should be more balanced and indicate evidence including that provided for NLS's that the SLiMs identified by ProPD DO generally function w/in the whole protein context although there might be some false positives. Anyway to estimate the occurence of false positives?

Overall this is an impressive accomplishment and a timely piece of work.

Reviewer #3:

Summary

- Describe your understanding of the story
The authors present an improved screen for domain-motif interactions and its results for a much larger set of proteins (34) than they have produced previously (7 in HD1), as well as a web tool (PepTools) that allows exploration of the results, as well as offering analysis of data from similar experiments that a user might provide. This website in itself is a highly valuable resource. The authors' ProP-PD method currently produces the most finely-grained data on domain-motif interactions, on which the authors are leaders in the field. It allows screening of a protein or domain of interest against a library of almost a million

peptides, which is very impressive. In addition, the authors present a large number of more detailed follow-up experiments that zoom in on individual cases.
This article and the data it presents are therefore extremely important and likely transformative for the field of short linear protein motifs and their role in domain- motif-based protein-protein interactions.

- What are the key conclusions: specific findings and concepts
ProP-PD is able to identify domain-motif interactions over a broad range of affinities, down to millimolar (low) affinity. It is therefore highly sensitive. The recall of known motif-mediated interactions is fairly disappointing at 19.3% (65 out of 337), but the authors state that this is similar to what is observed in other high-throughput screens. In fact, it might be substantially (two-fold) better, as the authors modestly show in Figure 2k.

- What were the methodology and model system used in this study
M13 phage display.

General remarks

- Are you convinced of the key conclusions?
Yes.

- Place the work in its context.
As mentioned above, the authors' ProP-PD method currently produces the most finely-grained data on domain-motif interactions, on which the authors are leaders in the field.

- What is the nature of the advance (conceptual, technical, clinical)?
Conceptual (aspects of screen design and analysis) and technical (phage coat protein P8 or P3 used for display, etc.).

- How significant is the advance compared to previous knowledge?
Highly significant. There is a 5-fold increase in the number of proteins studied and an increase in resolution from a sequence sliding window step size of 7 to one of 4, which begins to allow pinpointing of short linear motifs and which makes this a transformative dataset.

- What audience will be interested in this study?
Researchers interested in protein-protein interactions, their detection, signaling roles and evolution.

Major points

-Specific criticisms related to key conclusions
None.

-Specify experiments or analyses required to demonstrate the conclusions
n/a

-Motivate your critique with relevant citations and argumentation
n/a

Minor points

-Easily addressable points

Introduction:

1) The BioGRID website (https://wiki.thebiogrid.org/doku.php/statistics) says that it contains 612,648 non-redundant physical human protein-protein interactions, so "tens of thousands" in the abstract appears far too low. Perhaps it could be clarified that this refers to individual experimental datasets.
2) In the introduction, the authors state that "a hidden interactome of low-affinity, transient, and conditional interactions remains undiscovered". Could the authors please comment on why a yeast two-hybrid experiment would be unable to find these interactions? Likewise, the authors later state in the introduction that SLiM-based interactions are "difficult to capture experimentally by classical large-scale PPI discovery methods". This is clearly true for AP-MS studies, but much less so for Y2H, I think.
3) The authors then state that "a significant portion of these [unknown] interactions are [likely to be] mediated by short linear motifs in the intrinsically disordered regions of the human proteome". This would already be a good place to cite PMID 25038412, which is currently cited later in the introduction. Currently there is no reference to support this broad and speculative statement.
4) A reference for "IDRs cover one third of the human proteome" would be great, please, especially since disorder predictors

tend to under-predict disorder (PMID 30914747), so prediction-based numbers such as "one third" come with uncertainty.
5) Currently, the statement "Many SLiM-based PPIs rely on additional binding sites" is only backed up by references talking about very specific cases (WW domain proteins, of which there are 53 in humans, and the specific case of Keap1 and Neh2), which doesn't support the statement that "many" SLiM- based interactions require multiple binding sites (implying that this is the general mode of interaction). A reference that truly supports the statement would be very important since it makes a major point about how SLiM-based interactions are considered to work.
6) I think "at amino acid resolution (Fig.1)" and "defined the binding sites at amino acid resolution" (and throughout the text) is mildly overstated since the authors have not yet demonstrated that this resolution can be reached based on the data resulting from an experiment, not even for a single example motif as far as I can see (the sliding window step size in this current "HD2" library is 4 aa). "Potentially at amino acid resolution" would be more accurate and ensure that readers do not assume amino acid-resolution binding motifs can be directly derived from the data in this article (instead, the authors use an enrichment- based method, SLIMFinder). I think this should be changed or at least explained better as it seems to me the method as used here has a resolution of 4 aa, not 1 aa. I do agree that using SLIMFinder appears to result in promisingly accurate identification of the exact residues that make up some binding motifs, however.
7) It is stated that "the HD1 library suffers from limitations", but these remain unspecified. It would be very useful to briefly mention what they are. The only point I could find in the paper was that it performed worse in benchmarking (page 8), but this is not explained further.
8) When the authors state that "these [20% recall] results are similar to other large- scale approaches for protein-protein interaction screening", I think it would specifically be interesting and important to make a comparison to Y2H screens, which should in my mind be capable of detecting SLiM-based interactions and are therefore more relevant than other types of screen.
9) Overall, I was surprised to see that the recall of known motif-mediated interactions is fairly disappointing at 19.3% (65 out of 337), but the authors state that this is similar to what is observed in other high-throughput screens. The dataset of 337 known interactions used for this is based on high-quality curation which I would trust, therefore this low number remains very surprising to me. As sensitivity is not an issue (low nanomolar interactions are captured), I wonder if the authors could speculate more on the factors intrinsic to their (in vitro) screen that could explain such a low recall, please.

Results and discussion:

10) In terms of library design, perhaps it could be mentioned that the point of subdividing the library into pools based on subcellular localization is to reduce the number of molecules competing, if I understand correctly.
11) There is currently no reference or brief description for the M13 phage system. Knowledge of it is assumed, but since it is the workhorse of this screen, I think it would be great to have at least a sentence of detail on it, please. A 1996 reference is given for the coat proteins, but presumably there is a good introduction to the system somewhere that is a little more up-to-date. The figures shown to explain it (1a and 1b) are excellent, however.
12) Under "phage selections and initial evaluation of selection results", the collection of 34 domain instances and 30 domains is described as a "benchmarking set". It could be helpful for the reader to present them as such earlier (i.e. that these have been very consciously chosen to be useful as a benchmarking set). It also was not clear to me up to this point that only the binding domains were used from the 34 proteins (rather than full proteins). It would be great if this could be clarified, please.
13) When describing the HEAT repeat of KPNB1 as a "challenging test case", it would be useful to mention that this refers to its typically low ligand affinity, as done earlier. Similarly, it would be great to mention for clarity why GST in particular was chosen as a negative control. Otherwise, the selection of negative controls and the explanation are excellent.
14) From Fig. 1c, it appears that the replicate-to-replicate overlap of peptide hits is quite low. This seems to me to indicate that the experimental conditions could still be optimized. Perhaps the authors could comment on this in the discussion, if not already done.
15) In Fig. 1e, I find it difficult to understand the plot used (there is almost no segmentation in the blue bait plot, so it only seems to show that the range is somewhere between ~1e-3 and ~1e-16). I have not seen this type of plot before and I don't see a clear advantage over more standard types of plot such as a box or violin plot, which would be easier to parse, I think. If I understand the plot correctly the median is at ~1e-14, which would be very impressive. Perhaps the plots can be explained better.
16) It would be great if the authors could speculate in the discussion why the four proteins with "statistics that were similar to the negative controls", MAD2L1, SPSB1, SUFU and WDR5, might have had these results. Were they not expected to bind specific peptides? Similarly, some baits show very low replication (e.g. KLC1, KPNA4, etc.). Especially for KPNA4, this is surprising since this protein was singled out by the authors for validation. The authors do not seem to comment on KPNA4's low replication in the section were detailed follow-up experiments on it are described. As in my point about the low replicate overlap in Fig. 1c, this appears to point to issues in the experimental process that could hopefully be improved.
17) In "Benchmarking of metrics for ranking of ProP-PD results", the authors consider "unspecific promiscuous peptides" uninteresting and to be ignored. I think in order to make this judgment, the authors would need to be able to argue that these are unphysiological interactions and that they would therefore not occur biologically. Otherwise, I do not see why interactions should be discarded for their "promiscuity". The authors then do not actually appear to incorporate "promiscuity" as a negative in their four metrics for "high confidence ligands", so perhaps it should not be mentioned in the preceding sentence.
18) The p-values given are very impressive, but the test used (Mann-Whitney U) is not specified except in Fig. 2g, and the unspecified effect size appears low in Fig. 2b and 2c. In Figures 2b and 2c, I actually find it impossible to tell whether "motif" or "other" has a higher value in the non-standard box plots that were used. A different representation, or at least indicating the averages, would help.
19) In Fig. 2d, it would be helpful to plot "-log" rather than "log" so that a high score is desirable, as in the preceding plots.

20) What I am missing in Fig. 2 (ideally after 2d) is a plot comparing the peptides to the ELM motif, rather than the "de novo consensus established for the ProP-PD derived peptides using SLIMFinder".
21) Figure 2e still needs to be referenced in the text after the relevant p-value, and no explanation of the "normalised peptide count" is given in the text or legend. I assume a "normalised peptide count" of 1e-3 means that 1 in 1000 peptides is of the type of interest, but it would be great if this were explained (what the normalization is relative to).
22) In terms of presentation, when discussing the SFN 14-3-3 negative control, perhaps the authors might also suggest that since SFN binds a very large number of partners (according to UniProt: https://www.uniprot.org/uniprot/P31947), it might be unlikely that the interaction with the MAP1A peptide would occur much in vivo (since SFN might be "titrated out").
23) Such an argument could be made elsewhere in the manuscript (its Discussion section) as well: perhaps the low recall (19.3%) of the 337 ELM interactions is due to the absence of other factors that would change the effective local concentrations in vivo, including other interfaces in the full-length bait proteins that are absent in the screen. I think this low recall of a very high-confidence set of interactions is so surprising that any additional ideas to explain it would be very welcome, especially ideas that can extend to HuRI and BioPlex as well (Fig. 2l).
24) Figure 2k is extremely convincing, however. Perhaps it would be beneficial early on in the manuscript or abstract to state that the screen still achieves twice the recall of yeast two-hybrid and mass spectrometry-based screens. Recall appears to be the more relevant metric since even the HuRI authors state that they expect to have only sampled 2-11% of the human interactome (http:// www.interactome-atlas.org/faq/), making precision less reliable as a metric. This would be a very convincing argument that should be made more prominent, I think.
25) The section headline "ProP-PD selections rediscovery one fifth of known motifs..." is missing the word "allows" or similar.
26) In Fig. 2k, I assume incorporating BioGRID, to my knowledge the most comprehensive interaction database, would show a recall of 100% since it contains all ELM interactions, correct?
27) BioGRID is curiously absent from the paper. It would have been very helpful to see a direct comparison of BioGRID and ProP-PD, I think, since BioGRID is the most comprehensive database of protein-protein interactions. Including it would give the reader a better sense of the precision of the method (and it would probably be the best estimate of its precision). Perhaps this could be done at the confidence levels shown in Fig. 2h.
28) In Fig. 2m, it would have been helpful to see HuRI as well. Without this, I find the statement about "different parts of the interactome" less convincing. Did HuRI contain none of these interactions?
29) CaM might be better written as "calmodulin" for clarity, at least when first introduced.
30) The text refers to "Fig. 4e" as well as "Fig. 4c-e", but there is no panel e. There is also a reference to "tryptophan rich peptides (Fig. 4d)" which seems to be for the wrong figure (4b?). All of these figure references (as well as those to supplementary items) should be checked, please.
31) When describing the two NLS binding pockets of KPNA4, the authors mention "ARM 2-4 and ARM 6-8". For clarity, it could be mentioned that these are ARM repeats.
32) The speculation on NCOR2's very high-affinity interaction with KPNA4 pointing to NCOR2 being a general inhibitor of KPNA4 would require more evidence, I think. As far as I understand NCOR2 is simply a nuclear protein and I am not aware of any evidence that it broadly interferes with nuclear import. I also cannot see the pY1311 phosphosite (residue 1311 in https://www.uniprot.org/ uniprot/Q9Y618 is not a tyrosine, nor in its pre-2018 version), so I have not been able to find a reference on this phosphorylation event, nor is one provided in the text.
33) The motifs expressed as "regular expressions" in Table 1 would be better placed in a supplementary table, and instead, motif logos should be shown, I think. Currently it is very difficult for a reader to accurately compare the observed and expected motifs.

-Presentation and style
The presentation and figures are stellar throughout. My only criticism is that a few important points are unsourced, mildly overstated or not explained, and there are some minor mistakes (see list above).

--Trivial mistakes

-Page 3, typo: "interac[t]ome"_

-Page 8, typo: "showed it bind<s>"

-Page 13, typo: (Fig. 1)) has an extra parenthesis

-Page 14, typo: "33 peptides found in 32 protein<s>"

-Page 17: "human interactome proteome" should read "human interactome"

-I would like to thank all the authors for their excellent and extremely substantial work. I hope these comments will be helpful and I apologize in advance if they are too detailed. I thought that the quality and promise of the study allows it to become truly outstanding.

Reviewer #3: additional comments during cross-commenting

I fully agree with Reviewer #2's comment that the reviews are complementary, and that minor revisions should be recommended.

I also agree with Reviewer #1's review regarding the absence of a biological analysis "at scale" that could be expected for a screen of this size. The screen is rather "targeted" due to the use of specific bait domains, so it would be very interesting and reasonably easy to see how well the interactors match the biologically expected set of targets, and how coherent the set is. I especially think this would be interesting since I share Reviewer #1's concern regarding the low number of interactions explained by motifs (~7%) in some cases. However, since the authors have produced a first-of-its-kind screen for these domains, I think publication could also go ahead as-is if the authors argue that too much additional work would be needed. I am confident there will be high-quality follow-up studies by the authors, given their track record.

As I mentioned in my review, I wish the authors had talked more explicitly about the low explanatory value of the "known motifs" (their low hit rate) and the high apparent level of noise and low reproducibility across replicates, and speculated more about possible reasons for this. My hope is that the experiments themselves can be optimized to arrive at higher reproducibility across replicates, leading to better results. There is currently very little discussion of this, and since it's a first-of-its-kind screen that obviously can't be perfect just yet, I think it would be valuable to have.

Finally, I would definitely echo Reviewer #2's question 6: "P.14: how many peptides found with KPNA4 occur in proteins that are known to localize to the nucleus?"

I agree somewhat less with point 7 by Reviewer #2, since the authors here are trying to make the point of potentially missing avidity from other interfaces in the full-length protein, as well as other factors affecting local concentration (condensates, etc.).

I'd also like to strengthen points 22 and 23 that I made in my review - in future studies, it could be very useful for the authors to use data on average protein abundance when interpreting the raw screen data, I think. This could perhaps allow the authors to calculate an "effective affinity" that might help sift out spurious interactions that are unlikely to happen in vivo. The subcellular localization could be incorporated as a filtering factor as well.

Regarding my point 32 on inhibition by NCOR2, a simple test would be whether NCOR2 has a higher affinity for importin beta than RanGTP (published values should be available), which I would think is unlikely.

Most of all, I would like to thank all the authors and reviewers for their excellent and highly useful contributions here.

Reply to reviewers

We thank the reviewers for the helpful suggestions that have improved the quality of our manuscript. A point by point reply is provided below, with our responses in bold.

Reviewer #1 (Remarks to the Author):
Review of Benz et al "Proteome-scale footprinting of binding sites in the unstructured regions of the human proteome"

The manuscript describes a phage display based protein interaction screen / method that involves as bait 34 proteins defined by domains that are known to recognize linear amino acid epitopes. The peptide library (HD2) is designed from disordered human protein stretches in a tiling fashion with 4 amino acids overlap each. The library thus covers a difficult to study subset of potential binding interfaces, not displaying free N-terminal or C-terminal residues, and no PTMs modifications. A substantial H2D-P8screen is performed and read out through second gen sequencing. Four criterial for scoring binding peptides are established, reproducibility, peptide overlap, motif overlap, and read count. 396 / 2653 high/ medium peptides are defined as mediators of PPIs, which is about 2000 human protein-protein interactions. The data are benchmarked against a 337 literature motif data set, de novo motifs and known motifs are compared. Furthermore benchmarking against two major Y2H, AP-MS data sets and the literature meta data set HIPPIE are described, including the variation between different baits, exemplary for NEDD4 and PABPOC1. For a set of 4x4 interactions pairs KDs are determined showing a broad range of affinities up to 100 µM. The possibility to capture these low affinity interactions is a clear advantage of the approach. After a short methodological account (mostly negative results) KPN4A motif binding is studied in much detail, revealing that a large fraction of the peptides with motif matches represent NLS. Accordingly, R/K to AA prevent nuclear import in selected cases. A final very interesting analysis is performed: Amino acid mutations and PTMs are mapped on to the peptide binding sequences to predict interaction perturbation events, e.g. through phosphorylation or amino acid substitutions. Exemplary four substitutions and one phosphorylation is shown to substantially decrease the affinity for KPNA4 or KEAP1 respectively.

This is a prime study on motif-based protein interactions. The assay is addressing linear epitope (::motif) binding of specific protein domains and the authors do a very thorough and innovative approach on defining the motifs that contribute to the interaction.

Our response:
We thank the reviewer for the positive comments on our work.

However, a maximum of 6.8% (27/396 in the high confidence and lower 74/2653 in the medium confidence or 65/3049) it the fraction of the binding that is explained through the motifs. In other words, the majority of the binding events is not explained by motifs (even if the final number because of de novo motifs etc. is not clear). All analysis focuses on this minor subset and the majority of the binding events is ignored.

Our response:
This is a misunderstanding. We thank the reviewer for showing that we needed to explain this better. The numbers refer to the number of previously reported motifs found in each category. All peptides in the high confidence bin have a consensus motif (matching the ELM consensus and/or the SLiMFinder consensus) as the presence of consensus motif is one of the four criteria used for assigning high confidence. In the medium confidence bin of peptides that fulfilling 2 or 3 criteria, the majority have a consensus motif (matching ELM consensus 1420; matching SLiMFinder motif 2112). We have now clarified this in the text (section "ProP-PD

selections rediscover one fifth of known motifs as medium/high confidence ligands"). We have also added an overview of the proportion of motif-containing peptide in Figure S4a.

As far as I can grasp there is the one case, the DLIFTDSKLYIPLE peptide of TPTE2 which does not contain a motif and is assayed in figure 3 and it binds (of course) with intermediate affinity! What is the contribution of the motif binding peptides to the overall data, and how to think about the non-motif linear epitopes.

Our response:
As indicated above, most of the high ranking peptides do have consensus motifs. However, as shown for the TLN1 binding TPTE2 peptide, the medium confidence dataset might contain ligands with alternative motifs. This is particularly true for protein domains that, like the TLN PTB-like domain, have more than one binding site. However, ,ost protein domains tested have only one binding site, and for them it is less likely to find peptides with distinct motifs. In order to test this, we used KEAP1 Kelch domain as a model protein. We obtained an additional set of peptides having a variant motif, or no motif, for binding the KEAP1 Kelch domain. We found that while the variant motif containing peptide bound tightly, the peptides without apparent motifs did not bind, or bound with low affinity, as expected. We have added the results to the section "ProP-PD selections capture interactions with a broad range of affinities", to Figure 3a, and to Figure S4c.

With reference to the wording in the text about the hidden interactome, it feels as if the authors are creating another hidden layer through ignoring most of the data in their analyses.

Our response:
As explained above, we do not ignore data but confidence rank the data based on different quality metrics.

Along the same line, the authors as the state "present a resource of more than 2,000 human PPIs with amino acid resolution of binding sites" but do not analyze the data in biological terms at all. In this respect the data set does not compare well to other analysis that perform screens at this scope.

Our response:
Thank you for pointing this out. We have added a section titled "Gene Ontology (GO) enrichment analysis of the ProP-PD based interactome" where we have performed two GO term analysis, a significant shared terms GO term analysis between bait and prey and a classical GO term enrichment analysis for all bait interactors, to investigate the biological aspects of the discovered peptides. The results of the analysis are also provided in EV dataset 7.

Points for consideration:
*) The whole part about dividing the library into subcellular localization bins is not clear to me. Attempts to reduce the complexity of the peptides display make sense in principle, however were shown to compare similar to the full H2D-P8 screen. This is an in vitro system therefore it did not become very clear what the particular subsets according to subcellular localization could possibly bring.

Our response:
Contrasting with classical combinatorial peptide-phage display the aim of ProP-PD is not only to find biophysically binding peptides, but also to find interactions of potential biological relevance. The point of subdividing the library into pools based on

cellular localization is to reduce the number of competing peptides that the bait protein would not meet in a cellular setting. The hypothesis was that by limiting the search space to peptides from proteins that share localization with the bait in the cell we would enrich for more peptides from potentially biologically relevant interactors. That it turned out not to be necessary for most cases is a relief from an experimental point of view. We have clarified this in the text (section "Library design parameters can influence data quality").

*) Figure 1 g, what about displaying the number of partner peptides found for each domain. The percentages and more so -log p values are sometimes misleading. What about percent peptides bound with motif vs other epitope binders.

Our response:
The role of Figure 1 g is to emphasize that all screens have not been equally successful in enriching for high quality peptides. The figure allows a reader to visually compare the difference between the bait and controls and see the set of baits that have properties more similar to a control screen than the other baits. We have attempted to emphasize this point in the discussion of the Figure in the main text (section "Phage selections and initial evaluation of selection results"). We have also added an overview of the results used to generate the figure in EV Dataset 2.

Some counts according to domains would allow for a better data overview. How do PPIs distribute across the 35 baits? A PPI summary is somehow missing/ would be useful for a better understanding of the screening results.

Our response:
We agree that a PPI summary is missing, and have added a brief overview of the data to the text (section "HD2 P8 selections generate large-scale data with similar quality to other interactomics studies".

PPIs for several individual baits are discussed in detail in the text.

*) Peptide scoring: two remarks: i) the specificity determinant score (similarity of the peptides to SLiMFinder motifs) performs better than the final score (all four metrices score) against the benchmark. ii) Affinities do not correlate with read count, so why does read count as metric #4 perform well?

Our response:
i) When available, the specificity determinant score is the strongest metric. For this study, we could have largely relied on that score. However, only relying on the specificity determinant score would mean excluding all peptides that might have alternative motifs. In addition, it is not always possible to generate consensus motifs (depending on for example few enriched peptides). To establish a generally applicable robust protocol we included more metrics in the analysis.

ii) During the selections we start with a phage library that contains about 1 million peptides. From the selection we typically end with around 1000 peptide sequences associated with different NGS counts. This set contains both real binders and spurious peptides identified due to the depth of the sequencing. The NGS counts do well in separating binders from non-binders where there are major affinity differences, and it is thus a valid metric for peptide scoring (Figure 2d). However, the resolution is not high enough to discriminate between relatively minor affinity differences within the binding enriched cohort.

*) "The medium/high confidence HD2 P8 data has twice the recall (the proportion of PPIs that have been rediscovered) of BioPlex and HuRI on the motif-based interactions set, but with lower precision (the proportion of re-discovered PPIs among all PPIs found), particularly when compared to the HuRI data. " This sentence is confusing and makes only sense if the comparison refers to HuRI vs BioPlex. Hence the precision is twice as high for HuRI in this comparison. I thought it is well established that Y2H data better resemble liner epitope mediated PPIs (c.f. ELM) and I think that is what the authors want to say.

Our response:
We have modified the text to avoid confusion (see section "HD2 P8 selections generate large-scale data with similar quality to other interac-tomics studies").

*) Figure 2I: N for the three data sets are missing / confusing. Based on Protein overlap, how many PPIs can be found / were found.

Our response:
The N in figure 2I related to the number of PPIs in the curated ProP-PD benchmarking set. To avoid confusion, we have removed the number from the figure and provide the information in the figure legend instead.

*) It would be much easier to present the four peptide sequences assayed in Figure 3C.

Our response:
We thank the reviewer for the constructive suggestion. We have added the peptide sequences to Figure 3C.

*) It would also be a better validation to sample peptide sequences from the set of iterators including non-motif binders for validation.

Our response:
As most peptides in the medium/high confidence bins contain the expected motifs it seemed logical to focus on this main cohort to understand the affinity ranges we cover for different bait proteins. Nevertheless, following the suggestion we determined the affinities of an additional set of peptides for KEAP1 kelch and included a peptide with a variant motif (NGE instead of TGE), as well as peptides lacking the consensus motif. As expected, we found that the variant motif containing peptide bound with high affinity and the peptides without motifs are bound with low affinity or not at all. See Figure 3 and Figure S4.

*) Even a basic analysis of the PPI data as such is missing.

Our response:
In addition to the interaction benchmarking against HIPPIE, HuRi and BioPlex already present later in the paper we have added additional analyses of the PPI data. Firstly, we have added an analysis of the raw peptides looking for an enrichment of interactions that have previously been validated for peptides-containing proteins returned for a bait protein over chance based on peptide randomisation. Secondly, we have added a section titled "Gene Ontology (GO) enrichment analysis of the ProP-PD based interactome" where we have performed two GO term analyses, a significant shared terms GO term between bait and prey and a classical GO term enrichment analysis for all bait interactors, and an interactome overlap enrichment analysis for the medium/high confidence peptides.

*) The focus on KEAP1 misses out NRF2.

Our response:
We agree. We have added the missing information.

Reviewer #2:

In the manuscript by Benz et al, Proteome-scale footprinting of binding sites in the unstructured regions of the human proteome, the authors present an impressively thorough description of second generation peptide phage display libraries constructed from intrinsically disordered regions of the human proteome, i.e. ProPD which they screen with 35 known SLiM-binding domains. They compare the performance of this library with other phage display libraries and with other unbiased methods (Yeast 2 hybrid, Affinity Purification coupled to mass spectrometry, AP-MS) and convincingly demonstrate the power of this approach to identify human SLiM-containing partners and discover new binding partners and new SLiMs. They also establish the affinity of some of the peptides discovered using flourescence polarization. In all this is a clearly written study and a technical tour de force that establishes the power of ProPD to systematically identify and characterize short linear motifs. The significance of the work is further demonstrated by in depth analysis of several new nuclear localization sequences (NLS) from ProPD analysis with the importin KPNA4 which also shows that these NLS sequences function within the context of the entire protein. Finally, several disease associated mutations that lie within SLiMs and alter SLiM function/affinity are identified. Overall this paper represents the 'bible' on identifying SLiMs using ProPD and adds significantly to our current knowledge of SLiMs and SLiM instances in the human proteome. As such information about this method and the resulting comprehensive information contained in this manuscript will be of interest to a broad audience. My relatively minor comments/suggestions are as follows:

We thank the reviewer for the positive comments on our work.

1) Title. I find the word 'footprinting' in the title misleading as it implies a specific biochemical technique. I think the main point is that binding sites are revealed at the amino acid level. "Proteome-level elucidation of binding sites with amino acid resolutionin unstructured regions of the human proteome " ?

Our response:
We have replaced the word "footprinting" in the title with "mapping". The full title now reads "Proteome-scale mapping of binding sites in the unstructured regions of the human proteome

2) Table 1 presents known motifs that were identified by ProPD. However there are many differences between the ProPD motifs and the expected motifs. Authors should comment on this. First, ProPD has the power to identify a broader range of amino acids in motif positions compared to the low through put regular expression matching that is often used to define a motif. Several examples of this are seen. Second, ProPD can reveal additional contributions from 'flanking' residues again not identifiable by RegEx matching. Finally, some flanking residues in 'expected' motifs are not included in the ProPD motif. How might this occur? Authors might point out that methods which systematically assess the contribution of different amino acids to motif affinity are complementary to ProPD and can further reveal aspects of motif specificity.

Our response:
It is not trivial to directly compare the motifs generated by SLiMFinder based on ProP-PD and the literature curated ELM regular expressions. To facilitate a comparison we have added a new table (EV dataset 3; http://slim.icr.ac.uk/data/proppd_hd2_pilot tab ProP-PD consensus similarities). From that, it can be seen that the main specificity determinants generally agree between the peptides found through ProP-PD data and peptides listed in ELM listed. Of course, the unbiased ProP-PD screening is, as pointed out by the reviewer, not limited to the comparison with the previous literature, which may reveal variant motifs, or additional specificity determinants. That some of

the flanking regions suggested by the ELM motifs are not present in the ProP-PD may in part be explained by over-defined regular expressions due to the limited number of examples curated by the ELM resource. We are commenting on this in the detail for selected cases (e.g. calmodulin, Talin PTB domain etc).

Other methods, such as peptide SPOT arrays or deep mutational scanning may of course be used to further refine the motifs. However, we did not find a good way to add the information without breaking the flow of the discussion, so with all respect for it being a valid point we decided not to point this out in the text.

3) Minor comment. On page 9 "bold residue denote residues matching the bait consensus" should be 'matching the expected motif consensus for this bait'

Our response:
We have corrected this, thank you for pointing it out.

4)P. 11: I found the discussion of peptides that bind to the 'phosphotyrosine binding domain' in Talin confusing: The peptides discussed contain tyrosine but it is not phosphorylated, i.e. this particular motif does not require phosphorylation?

Our response:
The phosphotyrosine binding domain (PTB) was initially named after its observed ability to bind phophotyrosines. Unfortunately, this became rather confusing once PTB domains that do not not require phosphophorylated tyrosines for binding were discovered. The PTB domain of talin is one such example as it lacks the required basic residues for recognition of phosphorylated NPxY peptides. We have added this information to the manuscript (section "ProP-PD selections capture interactions with a broad range of affinities").

5) on P 13 authors state that the P3 library reinforces findings from P8 library. In fact these results seem distinct and are COMPLEMENTARY. Any explanation for why the peptides discovered in P3 are not found in the P8 library? Is there some particular aspect of P8 fusions that prevents some domains from binding? Or are these peptides under represented in the P8 library relative to the P3 library?

Our response:
The distribution of peptides in the P8 and P3 displayed are similar (see Appendix Figure S1). The peptides are fused to the phage proteins using the same flanking linker region. There is thus no obvious technical explanation for the observation. The explanation likely lies in the monovalent display enrichment for higher affinity ligands, as the affinity is not reinforced by avidity effects, althouh we did not experimentally test this hypothesis.

6) P.14: how many peptides found with KPNA4 occur in proteins that are known to localize to the nucleus?

Our response:
Using the HD2 P8 library we found peptides from 32 different proteins. Of the 26 proteins for which localization information was available in the UniProt database, 22 proteins were annotated as having nuclear or nucleolar localization. We have added the information to the manuscript (section "From binding to function: identified KPNA4 binding peptides are functional NLSs").

The rest of the KPNA4 binding peptides were generated using sub-libraries designed to contain proteins that are localized to the nucleus or are found in the nucleus and cytoplasm. Consequently all these ligands are from proteins reported to be in the nucleus.

7) Finally in the discussion the authors give a strong 'disclaimer' to their results saying they are based on in vitro interactions and must be tested in the context of the whole protein. While this is true, the fact that all the sequences are in disordered regions makes it likely they are functional. Also didnt they also show us a strong correlation between the PPIs identified by ProPD and using whole protein methods such as Yeast 2 hybrid and AP-MS? I think the statement should be more balanced and indicate evidence including that provided for NLS's that the SLiMs identified by ProPD DO generally function w/in the whole protein context although there might be some false positives. Anyway to estimate the occurence of false positives?

Our response:
As suggested, we have balanced the statement.

Like all large scale datasets, the ProP-PD data will include false positives, but there is no straightforward way to estimate the rate of false positives that we could thnk of.

Overall this is an impressive accomplishment and a timely piece of work.

Our response:
Thank you for the kind words and the appreciation of our study.

Reviewer #3:

Summary

- Describe your understanding of the story
The authors present an improved screen for domain-motif interactions and its results for a much larger set of proteins (34) than they have produced previously (7 in HD1), as well as a web tool (PepTools) that allows exploration of the results, as well as offering analysis of data from similar experiments that a user might provide. This website in itself is a highly valuable resource.
The authors' ProP-PD method currently produces the most finely-grained data on domain-motif interactions, on which the authors are leaders in the field. It allows screening of a protein or domain of interest against a library of almost a million peptides, which is very impressive. In addition, the authors present a large number of more detailed follow-up experiments that zoom in on individual cases.
This article and the data it presents are therefore extremely important and likely transformative for the field of short linear protein motifs and their role in domain- motif-based protein-protein interactions.

- What are the key conclusions: specific findings and concepts
ProP-PD is able to identify domain-motif interactions over a broad range of affinities, down to millimolar (low) affinity. It is therefore highly sensitive. The recall of known motif-mediated interactions is fairly disappointing at 19.3% (65 out of 337), but the authors state that this is similar to what is observed in other high-throughput screens. In fact, it might be substantially (two-fold) better, as the authors modestly show in Figure 2k.

- What were the methodology and model system used in this study
M13 phage display.

General remarks

- Are you convinced of the key conclusions?
Yes.

- Place the work in its context.
As mentioned above, the authors' ProP-PD method currently produces the most finely-grained data on domain-motif interactions, on which the authors are leaders in the field.

- What is the nature of the advance (conceptual, technical, clinical)?
Conceptual (aspects of screen design and analysis) and technical (phage coat protein P8 or P3 used for display, etc.).

- How significant is the advance compared to previous knowledge?
Highly significant. There is a 5-fold increase in the number of proteins studied and an increase in resolution from a sequence sliding window step size of 7 to one of 4, which begins to allow pinpointing of short linear motifs and which makes this a transformative dataset.

- What audience will be interested in this study?
Researchers interested in protein-protein interactions, their detection, signaling roles and evolution.

-Easily addressable points

Introduction:

1) The BioGRID website (https://wiki.thebiogrid.org/doku.php/statistics) says that it contains 612,648 non-redundant physical human protein-protein interactions, so "tens of thousands" in the abstract appears far too low. Perhaps it could be clarified that this refers to individual experimental datasets.

Our response:
Correct. We have updated the abstract to better reflect this.

2) In the introduction, the authors state that "a hidden interactome of low-affinity, transient, and conditional interactions remains undiscovered". Could the authors please comment on why a yeast two-hybrid experiment would be unable to find these interactions? Likewise, the authors later state in the introduction that SLiM-based interactions are "difficult to capture experimentally by classical large-scale PPI discovery methods". This is clearly true for AP-MS studies, but much less so for Y2H, I think.

Our response:
We agree. AP-MS is generally not well suited for finding motif-based interactions. As correctly stated by reviewer #2 Y2H can be used to find and characterize these interactions. We have modified the introduction to better reflect this.

3) The authors then state that "a significant portion of these [unknown] interactions are [likely to be] mediated by short linear motifs in the intrinsically disordered regions of the human proteome". This would already be a good place to cite PMID 25038412, which is currently cited later in the introduction. Currently there is no reference to support this broad and speculative statement.

Our response:
We agree. We have updated the text and added a citation.

4) A reference for "IDRs cover one third of the human proteome" would be great, please, especially since disorder predictors tend to under-predict disorder (PMID 30914747), so prediction-based numbers such as "one third" come with uncertainty.

Our response:
True. We have modified the sentence and provided references (see "Introduction").

5) Currently, the statement "Many SLiM-based PPIs rely on additional binding sites" is only backed up by references talking about very specific cases (WW domain proteins, of which there are 53 in humans, and the specific case of Keap1 and Neh2), which doesn't support the statement that "many" SLiM- based interactions require multiple binding sites (implying that this is the general mode of interaction). A reference that truly supports the statement would be very important since it makes a major point about how SLiM-based interactions are considered to work.

Our response:
We agree. The statement is certainly true, and there is a large body of literature on this. We looked for a comprehensive review but could not find it (maybe a good topic for a future review). We have replaced the references that pointed to examples, with references to two reviews that discuss the topic on a more conceptual level (see "Introduction").

6) I think "at amino acid resolution (Fig.1)" and "defined the binding sites at amino acid resolution" (and throughout the text) is mildly overstated since the authors have not yet

demonstrated that this resolution can be reached based on the data resulting from an experiment, not even for a single example motif as far as I can see (the sliding window step size in this current "HD2" library is 4 aa). "Potentially at amino acid resolution" would be more accurate and ensure that readers do not assume amino acid-resolution binding motifs can be directly derived from the data in this article (instead, the authors use an enrichment- based method, SLIMFinder). I think this should be changed or at least explained better as it seems to me the method as used here has a resolution of 4 aa, not 1 aa. I do agree that using SLIMFinder appears to result in promisingly accurate identification of the exact residues that make up some binding motifs, however.

Our response:
We have modified the text in the introduction as it relates back to previous studies where we did not tile the IDRs as densely as in the current manuscript (see "Introduction").

When discussing the results of the current study we keep the "amino acid resolution" as we combine different sources of information to obtain the resolution. We had omitted a description of how the amino acid resolution is extracted due to length restrictions. The ability of ProPD to define the binding site at amino acid resolution is based on the method returning information on both the binding 16-mer peptides (sometimes with overlapping peptides reducing the possible range) and the specificity determinants of the binding motifs (by finding the enriched motif consensus in the returned peptide in the same experiment). By combining these two sources of information it is possible to define the exact residues in each peptide required for binding hence in most cases we can derive the binding site at the amino acid resolution.

7) It is stated that "the HD1 library suffers from limitations", but these remain unspecified. It would be very useful to briefly mention what they are. The only point I could find in the paper was that it performed worse in benchmarking (page 8), but this is not explained further.

Our response:
We agree. We have described the limitations better (see "Introduction").

8) When the authors state that "these [20% recall] results are similar to other large- scale approaches for protein-protein interaction screening", I think it would specifically be interesting and important to make a comparison to Y2H screens, which should in my mind be capable of detecting SLiM-based interactions and are therefore more relevant than other types of screen.

Our response:
The recall of true positives is twice as high for HD2 P8 phage display as compared to Y2H. The recall of Y2H and AP-MS are maybe surprisingly similar (9.9% vs 8.6%), but the precision is twice as high for Y2H, probably due to a combination of a stringent analysis of the data, and the fact that AP-MS pulls report on complexes rather than binary interactions.

9) Overall, I was surprised to see that the recall of known motif-mediated interactions is fairly disappointing at 19.3% (65 out of 337), but the authors state that this is similar to what is observed in other high-throughput screens. The dataset of 337 known interactions used for this is based on high-quality curation which I would trust, therefore this low number remains very surprising to me. As sensitivity is not an issue (low nanomolar interactions are captured), I wonder if the authors could speculate more on the factors intrinsic to their (in

vitro) screen that could explain such a low recall, please.

Our response:
In contrast to the reviewer we find the recall for ProP-PD surprisingly high given that we start from 1 million of competing peptides, and pull out 20% of known ligands plus a large number of novel ligands. It is really quite amazing that it works as well as it does given the complexity of the experiment. We have clarified this and added some potential explanations to why not all true positive interactions are found to the discussion.

10) In terms of library design, perhaps it could be mentioned that the point of subdividing the library into pools based on subcellular localization is to reduce the number of molecules competing, if I understand correctly.

Our response:
We agree. We have added the information to the text.

11) There is currently no reference or brief description for the M13 phage system. Knowledge of it is assumed, but since it is the workhorse of this screen, I think it would be great to have at least a sentence of detail on it, please. A 1996 reference is given for the coat proteins, but presumably there is a good introduction to the system somewhere that is a little more up-to-date. The figures shown to explain it (1a and 1b) are excellent, however.

Our response:
We agree. We have added a brief description to the Introduction, as well as a reference to a more recent review.

12) Under "phage selections and initial evaluation of selection results", the collection of 34 domain instances and 30 domains is described as a "benchmarking set". It could be helpful for the reader to present them as such earlier (i.e. that these have been very consciously chosen to be useful as a benchmarking set). It also was not clear to me up to this point that only the binding domains were used from the 34 proteins (rather than full proteins). It would be great if this could be clarified, please.

Our response:
We agree. We have provided the information in the introduction.

13) When describing the HEAT repeat of KPNB1 as a "challenging test case", it would be useful to mention that this refers to its typically low ligand affinity, as done earlier. Similarly, it would be great to mention for clarity why GST in particular was chosen as a negative control. Otherwise, the selection of negative controls and the explanation are excellent.

Our response:
Thank you for the suggestion. We have clarified this in the text (see section “Phage selections and initial evaluation of selection results”).

14) From Fig. 1c, it appears that the replicate-to-replicate overlap of peptide hits is quite low. This seems to me to indicate that the experimental conditions could still be optimized. Perhaps the authors could comment on this in the discussion, if not already done.

Our response:
True. The conditions can be further optimized, and in particular the number of replicates. We now comment on it in the discussion.

15) In Fig. 1e, I find it difficult to understand the plot used (there is almost no segmentation in the blue bait plot, so it only seems to show that the range is somewhere between ~1e-3 and ~1e-16). I have not seen this type of plot before and I don't see a clear advantage over more standard types of plot such as a box or violin plot, which would be easier to parse, I think. If I understand the plot correctly the median is at ~1e-14, which would be very impressive. Perhaps the plots can be explained better.

Our response:
Boxenplots (or letter-value plots) are increasingly common alternatives to boxplots that are designed to visualize distributions more accurately, in particular in the tails of the distribution. It achieves this by visualizing quantiles beyond the quartiles commonly used in a boxplot. This is also a distinct advantage over a violin plot as the exact range of each quantile is observable. We agree that the median line in the plots was almost impossible to see. We have updated the plots with a bold black line at the median to improve the readability. We have also added an improved description of the boxenplot to the figure description.

16) It would be great if the authors could speculate in the discussion why the four proteins with "statistics that were similar to the negative controls", MAD2L1, SPSB1, SUFU and WDR5, might have had these results. Were they not expected to bind specific peptides? Similarly, some baits show very low replication (e.g. KLC1, KPNA4, etc.). Especially for KPNA4, this is surprising since this protein was singled out by the authors for validation. The authors do not seem to comment on KPNA4's low replication in the section were detailed follow-up experiments on it are described. As in my point about the low replicate overlap in Fig. 1c, this appears to point to issues in the experimental process that could hopefully be improved.

Our response:
KPNA4 has a lot more binding partners as compared to most of the domains in the study, which may explain the lower reproducibility between replicates. We now comment on it in the discussion.

The low enrichment of ligands observed for MAD2L1, SPSB1, SUFU and WDR5 might relate to protein quality issues (including for example incompatibility with immobilization method), as they are all four well-known peptide binding domains. We have added the remark to the manuscript (see section "Phage selections and initial evaluation of selection results").

17) In "Benchmarking of metrics for ranking of ProP-PD results", the authors consider "unspecific promiscuous peptides" uninteresting and to be ignored. I think in order to make this judgment, the authors would need to be able to argue that these are unphysiological interactions and that they would therefore not occur biologically. Otherwise, I do not see why interactions should be discarded for their "promiscuity". The authors then do not actually appear to incorporate "promiscuity" as a negative in their four metrics for "high confidence ligands", so perhaps it should not be mentioned in the preceding sentence.

Our response:
We agree, and have removed "unspecific promiscuous peptides" from the sentence.

18) The p-values given are very impressive, but the test used (Mann-Whitney U) is not specified except in Fig. 2g, and the unspecified effect size appears low in Fig. 2b and 2c. In Figures 2b and 2c, I actually find it impossible to tell whether "motif" or "other" has a higher value in the non-standard box plots that were used. A different representation, or at least indicating the averages, would help.

Our response:
We have updated the plots with a bold black line at the median to improve the readability. In both panel b and c, over half of the dataset for both motif and other have the minimum score possible for these metrics (observed in 1 replicate and no overlapping peptide). The boxenplot in these case allows for a better understanding of the differences when compared to boxplot as they show additional quantiles of data. Each quantile is half the size of the previous and the width of each additional quantile is smaller.

19) In Fig. 2d, it would be helpful to plot "-log" rather than "log" so that a high score is desirable, as in the preceding plots.

Our response:
We have updated the plot to add this improvement

20) What I am missing in Fig. 2 (ideally after 2d) is a plot comparing the peptides to the ELM motif, rather than the "de novo consensus established for the ProP-PD derived peptides using SLIMFinder".

Our response:
As the majority of the peptides in the benchmarking set are derived from ELM, and the ELM consensus is based on these peptides, the comparison based on the ELM motif would be highly biased. In addition, this section of the manuscript was looking at defining generalized guidelines and metrics for discriminating biologically relevant binder from non-binder. We wished to derive these metrics directly from the returned peptides and not require *a priori* external knowledge. Consequently, we used the de novo consensus established for the ProP-PD derived peptides using SLIMFinder. This will allow us to use these metrics in future work where we will screen baits that have no characterised motifs. As we show the de novo consensus established for the ProP-PD derived peptides using SLIMFinder has high discriminatory power.

21) Figure 2e still needs to be referenced in the text after the relevant p-value, and no explanation of the "normalised peptide count" is given in the text or legend. I assume a "normalised peptide count" of 1e-3 means that 1 in 1000 peptides is of the type of interest, but it would be great if this were explained (what the normalization is relative to).

Our response:
We have added a reference to Fig. 2e and an explanation of the normalization (see section "Benchmarking of metrics for ranking of ProP-PD results").

22) In terms of presentation, when discussing the SFN 14-3-3 negative control, perhaps the authors might also suggest that since SFN binds a very large number of partners (according to UniProt: https://www.uniprot.org/uniprot/P31947), it might be unlikely that the interaction with the MAP1A peptide would occur much in vivo (since SFN might be "titrated out").

Our response:
We have added a comment on this at the end of the section "Benchmarking of metrics for ranking of ProP-PD results".

23) Such an argument could be made elsewhere in the manuscript (its Discussion section) as well: perhaps the low recall (19.3%) of the 337 ELM interactions is due to the absence of other factors that would change the effective local concentrations in vivo, including other interfaces in the full-length bait proteins that are absent in the screen. I think this low recall of a very high-confidence set of interactions is so surprising that any additional ideas to explain

it would be very welcome, especially ideas that can extend to HuRI and BioPlex as well (Fig. 2l).

Our response:
We have added the information to the discussion (see point 9).

24) Figure 2k is extremely convincing, however. Perhaps it would be beneficial early on in the manuscript or abstract to state that the screen still achieves twice the recall of yeast two-hybrid and mass spectrometry-based screens. Recall appears to be the more relevant metric since even the HuRI authors state that they expect to have only sampled 2-11% of the human interactome (http:// www.interactome-atlas.org/faq/), making precision less reliable as a metric. This would be a very convincing argument that should be made more prominent, I think.

Our response:
Thank you for pointing this out. We have added the information to the introduction.

25) The section headline "ProP-PD selections rediscovery one fifth of known motifs..." is missing the word "allows" or similar.

Our response:
Corrected. Thank you.

26) In Fig. 2k, I assume incorporating BioGRID, to my knowledge the most comprehensive interaction database, would show a recall of 100% since it contains all ELM interactions, correct?

Our response:
The manually curated motif mediated interaction dataset used for the benchmarking PPIs is sourced from ELM and PDB. The overlap between these two resources with classical PPI datasets is not complete. This is even the case with the HIPPIE database that integrates human interaction data from 10 source databases including BioGRID, MINT, HPRD and IntAct. As we show in Fig. 2n the recall in this comparison (that includes all human BioGrid) interactions is only 10.8%. The major reason behind this is that all dataset have only curated a subset of the PPI literature and each database has a distinct focus. The ELM datasets focus on SLiM mediated PPIs results in superior coverage of this class of interactions. Interestingly, the PPI PDB complex structures are not automatically added to IMEX consortia PPI database even though this is possible as a result of a requirement for complete curation of papers so data presented in these papers that is not structural is collected. As a result the ELM and PDB datasets are not fully overlapping with the data from any PPI databases.

27) BioGRID is curiously absent from the paper. It would have been very helpful to see a direct comparison of BioGRID and ProP-PD, I think, since BioGRID is the most comprehensive database of protein-protein interactions. Including it would give the reader a better sense of the precision of the method (and it would probably be the best estimate of its precision). Perhaps this could be done at the confidence levels shown in Fig. 2h.

Our response:
In our PPI analyses, we use HIPPIE as a comprehensive source of human PPIs. HIPPIE integrates human interaction data from 10 source databases including BioGRID, MINT, HPRD and IntAct. We have clarified the reason for the use of HIPPIE and the source of the HIPPIE data in the text.

28) In Fig. 2m, it would have been helpful to see HuRI as well. Without this, I find the statement about "different parts of the interactome" less convincing. Did HuRI contain none of these interactions?

Our response:
HuRI did not return any of these interactions. We have added a clarification to the figure legend.

29) CaM might be better written as "calmodulin" for clarity, at least when first introduced.

Our response:
True. We have corrected this.

30) The text refers to "Fig. 4e" as well as "Fig. 4c-e", but there is no panel e. There is also a reference to "tryptophan rich peptides (Fig. 4d)" which seems to be for the wrong figure (4b?). All of these figure references (as well as those to supplementary items) should be checked, please.

Our response:
Done. The reference to Fig. 4d is correct.

31) When describing the two NLS binding pockets of KPNA4, the authors mention "ARM 2-4 and ARM 6-8". For clarity, it could be mentioned that these are ARM repeats.

Our response:
Done.

32) The speculation on NCOR2's very high-affinity interaction with KPNA4 pointing to NCOR2 being a general inhibitor of KPNA4 would require more evidence, I think. As far as I understand NCOR2 is simply a nuclear protein and I am not aware of any evidence that it broadly interferes with nuclear import. I also cannot see the pY1311 phosphosite (residue 1311 in https://www.uniprot.org/ uniprot/Q9Y618 is not a tyrosine, nor in its pre-2018 version), so I have not been able to find a reference on this phosphorylation event, nor is one provided in the text.

Our response:
The tested putative phosphosite in NCOR2 was designed based on information from the homologous protein NCOR1. As correctly noted by the reviewer there is no evidence for this site being phosphorylated in NCOR2. To avoid confusion we performed the same analysis on the NCOR1 peptide instead, for which several phosphosites have been reported. The results have been added to figure 6.

33) The motifs expressed as "regular expressions" in Table 1 would be better placed in a supplementary table, and instead, motif logos should be shown, I think. Currently it is very difficult for a reader to accurately compare the observed and expected motifs.

Our response:
This is an excellent idea. We have not been able to add the logos to the table in a readable way so we have added an interactive version of what you have described to the online material at http://slim.icr.ac.uk/data/proppd_hd2_pilot.

-Presentation and style
The presentation and figures are stellar throughout. My only criticism is that a few important points are unsourced, mildly overstated or not explained, and there are some minor mistakes

(see list above).

-Trivial mistakes
Page 3, typo: "interac[t]ome"
Page 8, typo: "showed it bind<s>"
Page 13, typo: (Fig. 1)) has an extra parenthesis
Page 14, typo: "33 peptides found in 32 protein<s>"
Page 17: "human interactome proteome" should read "human interactome"

Our response:
Thank you for pointing out the typos. We have corrected them.

I would like to thank all the authors for their excellent and extremely substantial work. I hope these comments will be helpful and I apologize in advance if they are too detailed. I thought that the quality and promise of the study allows it to become truly outstanding.

Our response:
Thank you very much for taking the time and effort to carefully read and comment on our manuscript. It has contributed to improve the quality of the paper.

Reviewer #3: additional comments during cross-commenting

I fully agree with Reviewer #2's comment that the reviews are complementary, and that minor revisions should be recommended.

I also agree with Reviewer #1's review regarding the absence of a biological analysis "at scale" that could be expected for a screen of this size. The screen is rather "targeted" due to the use of specific bait domains, so it would be very interesting and reasonably easy to see how well the interactors match the biologically expected set of targets, and how coherent the set is. I especially think this would be interesting since I share Reviewer #1's concern regarding the low number of interactions explained by motifs (~7%) in some cases. However, since the authors have produced a first-of-its-kind screen for these domains, I think publication could also go ahead as-is if the authors argue that too much additional work would be needed. I am confident there will be high-quality follow-up studies by the authors, given their track record.

Our response:
In response to reviewer #1 we have added a section "Gene Ontology (GO) enrichment analysis of the ProP-PD based interactome". We agree that additional validations would be too much work on top of the current results.

As I mentioned in my review, I wish the authors had talked more explicitly about the low explanatory value of the "known motifs" (their low hit rate) and the high apparent level of noise and low reproducibility across replicates, and speculated more about possible reasons for this. My hope is that the experiments themselves can be optimized to arrive at higher reproducibility across replicates, leading to better results. There is currently very little discussion of this, and since it's a first-of-its-kind screen that obviously can't be perfect just yet, I think it would be valuable to have.

Our response:
We have added this to the discussion.

Finally, I would definitely echo Reviewer #2's question 6: "P.14: how many peptides found with KPNA4 occur in proteins that are known to localize to the nucleus?"

Our response:
We have added the information for the HD2 P8 data to the results.

I agree somewhat less with point 7 by Reviewer #2, since the authors here are trying to make the point of potentially missing avidity from other interfaces in the full-length protein, as well as other factors affecting local concentration (condensates, etc.).

Our response:
Thank you for the support.

I'd also like to strengthen points 22 and 23 that I made in my review - in future studies, it could be very useful for the authors to use data on average protein abundance when interpreting the raw screen data, I think. This could perhaps allow the authors to calculate an "effective affinity" that might help sift out spurious interactions that are unlikely to happen in vivo. The subcellular localization could be incorporated as a filtering factor as well.

Our response:
This is an interesting suggestion that we will try to incorporate in our future studies.

Regarding my point 32 on inhibition by NCOR2, a simple test would be whether NCOR2 has a higher affinity for importin beta than RanGTP (published values should be available), which I would think is unlikely.

Our response:
We have removed the comment on the NCOR2 inhibition.

Most of all, I would like to thank all the authors and reviewers for their excellent and highly useful contributions here.

Our response:
Thank you!

Thank you for sending us your revised manuscript. We have now heard back from the reviewer who was asked to evaluate your revised study. As you will see below, they are satisfied with the performed revisions and support publication. As such, I am glad to inform you that we can soon formally accept the study, pending some minor editorial issues listed below. We would ask you to address these issues in a minor revision.

REFEREE REPORTS

Reviewer #1:

I thank the authors for addressing the points raised and some major clarifications on how to interpret the data. I understand now that motif matches constitute the major part of the binding peptides. Still, a critical discussion about the explanatory value of the motifs, in particular at a proteome scale, is somewhat missing. I guess next time. Congratulations to the authors for their work and I suggest to go ahead.

The authors have made all requested editorial changes.

Thank you again for sending us your revised manuscript. We are now satisfied with the modifications made and I am pleased to inform you that your paper has been accepted for publication.

EMBO PRESS

YOU MUST COMPLETE ALL CELLS WITH A PINK BACKGROUND ↓

PLEASE NOTE THAT THIS CHECKLIST WILL BE PUBLISHED ALONGSIDE YOUR PAPER

Corresponding Author Name: Ylva Ivarsson
Journal Submitted to: Molecular Systems Biology
Manuscript Number: MSB-2021-10584

Reporting Checklist For Life Sciences Articles (Rev. June 2017)

This checklist is used to ensure good reporting standards and to improve the reproducibility of published results. These guidelines are consistent with the Principles and Guidelines for Reporting Preclinical Research issued by the NIH in 2014. Please follow the journal's authorship guidelines in preparing your manuscript.

A- Figures
1. Data
The data shown in figures should satisfy the following conditions:

- the data were obtained and processed according to the field's best practice and are presented to reflect the results of the experiments in an accurate and unbiased manner.
- figure panels include only data points, measurements or observations that can be compared to each other in a scientifically meaningful way.
- graphs include clearly labeled error bars for independent experiments and sample sizes. Unless justified, error bars should not be shown for technical replicates.
- if n< 5, the individual data points from each experiment should be plotted and any statistical test employed should be justified
- Source Data should be included to report the data underlying graphs. Please follow the guidelines set out in the author ship guidelines on Data Presentation.

2. Captions

Each figure caption should contain the following information, for each panel where they are relevant:

- a specification of the experimental system investigated (eg cell line, species name).
- the assay(s) and method(s) used to carry out the reported observations and measurements
- an explicit mention of the biological and chemical entity(ies) that are being measured.
- an explicit mention of the biological and chemical entity(ies) that are altered/varied/perturbed in a controlled manner.

- the exact sample size (n) for each experimental group/condition, given as a number, not a range;
- a description of the sample collection allowing the reader to understand whether the samples represent technical or biological replicates (including how many animals, litters, cultures, etc.).
- a statement of how many times the experiment shown was independently replicated in the laboratory.
- definitions of statistical methods and measures:
  - common tests, such as t-test (please specify whether paired vs. unpaired), simple χ2 tests, Wilcoxon and Mann-Whitney tests, can be unambiguously identified by name only, but more complex techniques should be described in the methods section;
  - are tests one-sided or two-sided?
  - are there adjustments for multiple comparisons?
  - exact statistical test results, e.g., P values = x but not P values < x;
  - definition of 'center values' as median or average;
  - definition of error bars as s.d. or s.e.m.

Any descriptions too long for the figure legend should be included in the methods section and/or with the source data.

In the pink boxes below, please ensure that the answers to the following questions are reported in the manuscript itself. Every question should be answered. If the question is not relevant to your research, please write NA (non applicable). We encourage you to include a specific subsection in the methods section for statistics, reagents, animal models and human subjects.

USEFUL LINKS FOR COMPLETING THIS FORM

http://www.antibodypedia.com
http://1degreebio.org
http://www.equator-network.org/reporting-guidelines/improving-bioscience-research-repor
http://grants.nih.gov/grants/olaw/olaw.htm
http://www.mrc.ac.uk/Ourresearch/Ethicsresearchguidance/Useofanimals/index.htm
http://ClinicalTrials.gov
http://www.consort-statement.org
http://www.consort-statement.org/checklists/view/32-consort/66-title
http://www.equator-network.org/reporting-guidelines/reporting-recommendations-for-tumo
http://datadryad.org
http://figshare.com
http://www.ncbi.nlm.nih.gov/gap
http://www.ebi.ac.uk/ega
http://biomodels.net/
http://biomodels.net/miriam/
http://jjj.biochem.sun.ac.za
https://osp.od.nih.gov/biosafety-biosecurity-and-emerging-biotechnology/
http://www.selectagents.gov/

B- Statistics and general methods

Please fill out these boxes ↓ (Do not worry if you cannot see all your text once you press return)

1.a. How was the sample size chosen to ensure adequate power to detect a pre-specified effect size?	N/A
1.b. For animal studies, include a statement about sample size estimate even if no statistical methods were used.	N/A
2. Describe inclusion/exclusion criteria if samples or animals were excluded from the analysis. Were the criteria pre-established?	N/A
3. Were any steps taken to minimize the effects of subjective bias when allocating animals/samples to treatment (e.g. randomization procedure)? If yes, please describe.	N/A
For animal studies, include a statement about randomization even if no randomization was used.	N/A
4.a. Were any steps taken to minimize the effects of subjective bias during group allocation or/and when assessing results (e.g. blinding of the investigator)? If yes please describe.	N/A
4.b. For animal studies, include a statement about blinding even if no blinding was done	N/A
5. For every figure, are statistical tests justified as appropriate?	N/A
Do the data meet the assumptions of the tests (e.g., normal distribution)? Describe any methods used to assess it.	NA. There is no assumption.
Is there an estimate of variation within each group of data?	N/A

Is the variance similar between the groups that are being statistically compared?	N/A

C- Reagents

6. To show that antibodies were profiled for use in the system under study (assay and species), provide a citation, catalog number and/or clone number, supplementary information or reference to an antibody validation profile. e.g., Antibodypedia (see link list at top right), 1DegreeBio (see link list at top right).	Done
7. Identify the source of cell lines and report if they were recently authenticated (e.g., by STR profiling) and tested for mycoplasma contamination.	Done. Cells were tested for mycoplasma but not recently authenticated.

* for all hyperlinks, please see the table at the top right of the document

D- Animal Models

8. Report species, strain, gender, age of animals and genetic modification status where applicable. Please detail housing and husbandry conditions and the source of animals.	N/A
9. For experiments involving live vertebrates, include a statement of compliance with ethical regulations and identify the committee(s) approving the experiments.	N/A
10. We recommend consulting the ARRIVE guidelines (see link list at top right) (PLoS Biol. 8(6), e1000412, 2010) to ensure that other relevant aspects of animal studies are adequately reported. See author guidelines, under 'Reporting Guidelines'. See also: NIH (see link list at top right) and MRC (see link list at top right) recommendations. Please confirm compliance.	N/A

E- Human Subjects

11. Identify the committee(s) approving the study protocol.	N/A
12. Include a statement confirming that informed consent was obtained from all subjects and that the experiments conformed to the principles set out in the WMA Declaration of Helsinki and the Department of Health and Human Services Belmont Report.	N/A
13. For publication of patient photos, include a statement confirming that consent to publish was obtained.	N/A
14. Report any restrictions on the availability (and/or on the use) of human data or samples.	N/A
15. Report the clinical trial registration number (at ClinicalTrials.gov or equivalent), where applicable.	N/A
16. For phase II and III randomized controlled trials, please refer to the CONSORT flow diagram (see link list at top right) and submit the CONSORT checklist (see link list at top right) with your submission. See author guidelines, under 'Reporting Guidelines'. Please confirm you have submitted this list.	N/A
17. For tumor marker prognostic studies, we recommend that you follow the REMARK reporting guidelines (see link list at top right). See author guidelines, under 'Reporting Guidelines'. Please confirm you have followed these guidelines.	N/A

F- Data Accessibility

18: Provide a "Data Availability" section at the end of the Materials & Methods, listing the accession codes for data generated in this study and deposited in a public database (e.g. RNA-Seq data: Gene Expression Omnibus GSE39462, Proteomics data: PRIDE PXD000208 etc.) Please refer to our author guidelines for 'Data Deposition'.<br><br>Data deposition in a public repository is mandatory for:<br>a. Protein, DNA and RNA sequences<br>b. Macromolecular structures<br>c. Crystallographic data for small molecules<br>d. Functional genomics data<br>e. Proteomics and molecular interactions	Provided. We are finalizing the rich protein-protein interaction dataset for IntAct submission. This will be finished shortly.
19. Deposition is strongly recommended for any datasets that are central and integral to the study; please consider the journal's data policy. If no structured public repository exists for a given data type, we encourage the provision of datasets in the manuscript as a Supplementary Document (see author guidelines under 'Expanded View' or in unstructured repositories such as Dryad (see link list at top right) or Figshare (see link list at top right).	ok
20. Access to human clinical and genomic datasets should be provided with as few restrictions as possible while respecting ethical obligations to the patients and relevant medical and legal issues. If practically possible and compatible with the individual consent agreement used in the study, such data should be deposited in one of the major public access-controlled repositories such as dbGAP (see link list at top right) or EGA (see link list at top right).	N/A
21. Computational models that are central and integral to a study should be shared without restrictions and provided in a machine-readable form. The relevant accession numbers or links should be provided. When possible, standardized format (SBML, CellML) should be used instead of scripts (e.g. MATLAB). Authors are strongly encouraged to follow the MIRIAM guidelines (see link list at top right) and deposit their model in a public database such as Biomodels (see link list at top right) or JWS Online (see link list at top right). If computer source code is provided with the paper, it should be deposited in a public repository or included in supplementary information.	N/A

G- Dual use research of concern

22. Could your study fall under dual use research restrictions? Please check biosecurity documents (see link list at top right) and list of select agents and toxins (APHIS/CDC) (see link list at top right). According to our biosecurity guidelines, provide a statement only if it could.	No
